# Characterization of Gonadotropin-Releasing Hormone (GnRH) Genes From Cartilaginous Fish: Evolutionary Perspectives

**DOI:** 10.3389/fnins.2018.00607

**Published:** 2018-09-06

**Authors:** Anne-Laure Gaillard, Boon-Hui Tay, Daniela I. Pérez Sirkin, Anne-Gaëlle Lafont, Céline De Flori, Paula G. Vissio, Sylvie Mazan, Sylvie Dufour, Byrappa Venkatesh, Hervé Tostivint

**Affiliations:** ^1^Evolution des Régulations Endocriniennes UMR 7221 CNRS, Muséum National d’Histoire Naturelle, Paris, France; ^2^Institute of Molecular and Cell Biology, A^∗^STAR, Biopolis, Singapore, Singapore; ^3^Laboratorio de Neuroendocrinología del Crecimiento y la Reproducción, Facultad de Ciencias Exactas y Naturales, DBBE/IBBEA-CONICET, Universidad de Buenos Aires, Buenos Aires, Argentina; ^4^Biologie des Organismes et Ecosystèmes Aquatiques, CNRS, Muséum National d’Histoire Naturelle, Sorbonne Université, Paris, France; ^5^Biologie Intégrative des Organismes Marins, UMR 7232 CNRS, Observatoire Océanologique, Sorbonne Université, Banyuls-sur-Mer, France

**Keywords:** gonadotropin-releasing hormone, neuropeptides, evolution, multigenic family, cartilaginous fish, elasmobranchii, holocephali, vertebrates

## Abstract

The neuropeptide gonadotropin-releasing hormone (GnRH) plays an important role in the control of reproductive functions. Vertebrates possess multiple GnRH forms that are classified into three main groups, namely GnRH1, GnRH2, and GnRH3. In order to gain more insights into the *GnRH* gene family in vertebrates, we sought to identify which paralogs of this family are present in cartilaginous fish. For this purpose, we searched the genomes and/or transcriptomes of three representative species of this group, the small-spotted catshark, *Scyliorhinus canicula*, the whale shark, *Rhincodon typus* and the elephant shark *Callorhinchus milii*. In each species, we report the identification of three *GnRH* genes. In catshark and whale shark, phylogenetic and synteny analysis showed that these three genes correspond to *GnRH1, GnRH2*, and *GnRH3*. In both species, *GnRH1* was found to encode a novel form of GnRH whose primary structure was determined as follows: QHWSFDLRPG. In elephant shark, the three genes correspond to GnRH1a and GnRH1b, two copies of the *GnRH1* gene, plus *GnRH2*. 3D structure prediction of the chondrichthyan GnRH-associated peptides (GAPs) revealed that catshark GAP1, GAP2, and elephant shark GAP2 peptides exhibit a helix-loop-helix (HLH) structure. This structure observed for many osteichthyan GAP1 and GAP2, may convey GAP biological activity. This HLH structure could not be observed for elephant shark GAP1a and GAP1b. As for all other GAP3 described so far, no typical 3D HLH structure was observed for catshark nor whale shark GAP3. RT-PCR analysis revealed that *GnRH1, GnRH2*, and *GnRH3* genes are differentially expressed in the catshark brain. *GnRH1* mRNA appeared predominant in the diencephalon while *GnRH2* and *GnRH3* mRNAs seemed to be most abundant in the mesencephalon and telencephalon, respectively. Taken together, our results show that the *GnRH* gene repertoire of the vertebrate ancestor was entirely conserved in the chondrichthyan lineage but that the *GnRH3* gene was probably lost in holocephali. They also suggest that the three *GnRH* neuronal systems previously described in the brain of bony vertebrates are also present in cartilaginous fish.

## Introduction

Gonadotropin-releasing hormone (GnRH) is the major hypothalamic neurohormone regulating reproduction in vertebrates (Kah et al., 2007; Okubo and Nagahama, 2008). To date, a number of variants of GnRH have been identified that are classified into three distinct paralogous lineages, namely *GnRH1, GnRH2*, and *GnRH3* (Okubo and Nagahama, 2008; Roch et al., 2014a). *GnRH1* gene is known in most species of jawed vertebrates excluding a few species such as zebrafish (*Danio rerio*) (Okubo and Nagahama, 2008). GnRH1 is seen as the authentic GnRH that stimulates gonadotropin release (Kah et al., 2007; Okubo and Nagahama, 2008). *GnRH2* gene has been found in almost all vertebrate species investigated so far. GnRH2 is the only GnRH form whose structure is completely conserved in jawed vertebrates (Millar, 2003; Kah et al., 2007; Okubo and Nagahama, 2008). GnRH3 has long been believed to only exist in teleosts (Kah et al., 2007; Okubo and Nagahama, 2008; Kim et al., 2011; Tostivint, 2011) but recent studies have reported its occurrence in lamprey (Decatur et al., 2013; Smith et al., 2013) and coelacanth (Roch et al., 2014a; Yun et al., 2015). In contrast to GnRH1 which primarily acts as a neurohormone, GnRH2 and GnRH3 are generally viewed as neuromodulatory factors (Okubo and Nagahama, 2008). They have both been implicated in the control of reproductive behavior, however, their functions are far from being fully understood (Okubo and Nagahama, 2008).

It has been proposed that the three *GnRH* genes arose from the two whole-genome duplication events (2R) that took place early during vertebrate evolution (Kim et al., 2011; Tostivint, 2011; Roch et al., 2014a). According to this hypothesis, the common ancestor of all extant jawed vertebrates (gnathostomes) already possessed these three *GnRH* genes. Synteny analysis suggests that 2R also generated a fourth *GnRH* gene, but since this gene has never been found in extent vertebrate species, it is assumed to have been lost very early after 2R (Decatur et al., 2013).

Cartilaginous fish are of particular interest in evolutionary studies because their key phylogenetic position makes them ideal subjects to reveal the molecular bases of the important morphological and physiological innovations that characterize jawed vertebrates (Coolen et al., 2008; Venkatesh et al., 2014). Cartilaginous fish (forming class chondrichthyans) are the sister group of bony vertebrates (osteichthyans). They consist of two major groups: elasmobranchii (sharks and skates/rays) and holocephali (chimaeras) (Nelson, 2006). Previous studies showed that multiple forms of immunoreactive GnRH are present in cartilaginous fish, more particularly in elasmobranchs (for review, see Lovejoy et al., 2017). For example, as many as seven immunoreactive GnRHs were identified in the striped catshark (*Poroderma africanum*) (Powell et al., 1986). However, until the past few years, no more than two GnRH variants had been characterized: only a single GnRH form in the ratfish *Hydrolagus colliei* (Lovejoy et al., 1991) and the elephant shark *Callorhinchus milii* (Nock et al., 2011) and two GnRH variants in the spiny dogfish *Squalus acanthias* (Lovejoy et al., 1992). In a recent study, Roch et al. (2014a) revealed the occurrence of three *GnRH* genes in the little skate *Leucoraja erinacea* that correspond to the *GnRH1, GnRH2*, and *GnRH3* paralogs. They also found a second *GnRH* gene in *C. milii*, indicating the occurrence of both *GnRH1* and *GnRH2* genes in this species. Moreover, they reclassified the two GnRH forms previously identified in *S. acanthias* as GnRH2 and GnRH3, instead of GnRH1 and GnRH2.

In an attempt to better understand the molecular evolution of the *GnRH* gene family in vertebrates, we searched the genome and/or transcriptome databases of two representative species of elasmobranchs, namely the common dogfish, now renamed small-spotted catshark, *Scyliorhinus canicula* (Coolen et al., 2008) and the whale shark, *Rhincodon typus* (Read et al., 2017). We also took the opportunity to reexplore the genome and transcriptome databases of the holocephalan elephant shark *C. milii*, the first cartilaginous fish to have its full genome sequenced (Venkatesh et al., 2007). Here, we describe the characterization of three distinct *GnRH* genes in each species and the tissue distribution of their corresponding mRNAs in the spotted catshark. We also report a prediction of the 3D structure of the corresponding GnRH-associated peptides (GAPs).

## Materials and Methods

### Tissues

All the catshark tissues used in this study come from the same collection as that previously used in (Quan et al., 2013). Briefly, they were taken from mature catshark *S. canicula* of both sexes captured off Concarneau (Finistère, France) and stored in large natural seawater tanks at the Station de Biologie Marine of Concarneau (Muséum National d’Histoire Naturelle). At that time of the experiments, no regulation concerning the protection of animals used in scientific purposes existed in France. Such a regulation did not come into effect until February 1, 2013. Therefore, no application for authorization was necessary for the implementation of these studies. The animals were anesthetized in 0.01% MS222 then killed by decapitation, in accordance with relevant institutional and national guidelines on animal experimentation. The brain, spinal cord, skeletal muscles, heart, spleen, gills, stomach, duodenum, valvular intestine, liver, ovary, and testis from four specimens were dissected out. The organs were frozen in liquid nitrogen and kept at -80°C until use.

### Identification of GnRH-Related Sequences in Cartilaginous Fish

Catshark *GnRH* genes were sought by TBLASTN (Altschul et al., 1990) using the little skate GnRH sequences (Roch et al., 2014a) as queries against the catshark genome draft assembly (version 1) (unpublished data). Briefly, this assembly size is 3.68 Gb (*N*50 = 9558 bp; %*N* = 7.22) for an estimated genome size of 5.3 Gb (Stingo et al., 1980). It was obtained from a 151× coverage using a combination of Illumina paired end and mate pair sequencing, generated by the Genoscope (France). Assembly was conducted using CLC (Bio, Qiagen) for assembly and scaffolding of paired end sequences, SSPACE (Boetzer et al., 2011) and GapCloser (Luo et al., 2012) for the introduction of mate pair sequences. Whale shark *GnRH* genes were sought by TBLASTN using the catshark *GnRH* cDNA sequences as queries against the whale genome assembly database (Read et al., 2017) via NCBI^1^. Elephant shark genes were sought by TBLASTN using the catshark *GnRH* cDNA sequences as queries against the elephant shark genome assembly plus transcriptome data from 10 tissues (brain, gills, heart, intestine, kidney, liver, muscle, ovary, spleen, and testis; GenBank accession number SRA054255) (Venkatesh et al., 2014).

### Molecular Cloning of Full-Length *GnRH* cDNAs in *S. canicula*

Total RNAs from catshark forebrain and midbrain were extracted using RNAble (Eurobio, Courtaboeuf, France). Poly(A^+^) RNAs were selected from total RNA with Dynabeads mRNA Purification Kit (Invitrogen, Saint Aubin, France). 5′RACE-ready and 3′RACE-ready cDNAs were both constructed from 1 μg of poly(A^+^) RNA using the SMARTer RACE cDNA Amplification kit (Clontech, Saint-Quentin-en-Yvelines, France). The 5′- and 3′-ends of each cDNA were amplified by nested PCR using the Advantage 2 PCR kit (Clontech). The gene-specific primers were designed based on the *GnRH* sequences previously found (see **Supplementary Table S1**). PCR was carried out in a MyCycler thermal cycler (Bio-Rad, Marnes-la-Coquette, France) for 35 cycles (denaturation 94°C, 1 min; annealing between 57 and 60°C depending on the Tm of the primers, 1 min; and extension 72°C, 1 min), and a final extension of 72°C for 7 min. For the 5′ amplifications, the primers used were GnRH Rev × Universal Primer A Mix (UPM) then GnRH Rev Nest × Nested Universal Primer A Mix (NUP), and for the 3′ amplification, they were GnRH For × UPM then GnRH For Nest × NUP. The PCR products were subcloned into the pGEM-T vector (Promega, Charbonnières-les-Bains, France) and sequenced (Value Read Sequencing at MWG Biotech, Ebersberg, Germany). The coding sequence of the catshark *GnRH* cDNAs have been deposited in the GenBank database under the accession numbers MH468810, MH468811, and MH468812 for *GnRH1, GnRH2*, and *GnRH3*, respectively.

### Phylogenetic Analysis

Amino acid sequences of 74 chordate GnRH precursors [73 sequences from vertebrate species (Pérez Sirkin et al., 2017) including the eight complete chondrichthyan prepro-GnRH sequences characterized in the present study plus the amphioxus (*Branchiostoma floridae*) prepro-GnRH sequence (Roch et al., 2014b), used as outgroup] were aligned using the MAFFT algorithm^2^ then manually adjusted. The phylogenetic tree was built using PhyML (Guindon and Gascuel, 2003) via the Seaview version 4 software (Gouy et al., 2010). The best amino acid substitution model for the alignment was determined to be JTT+G+I using ProtTest (Abascal et al., 2005). The robustness of the tree was assessed by the bootstrap procedure with 1,000 replications. The GenBank accession numbers for all sequences used in the analysis are listed in **Supplementary Data Sheet S1**. The alignments are presented in **Supplementary Data Sheet S2**.

Phylogeny analysis was also performed specifically on chordate GAP sequences, including the eight chondrichthyan GAP sequences characterized in the present study, as previously described (Pérez Sirkin et al., 2017). GAP sequences were delimited between the dibasic site for proteolytic processing after the GnRH sequence and the stop codon of the open reading frame (ORF) (see **Supplementary Data Sheet S3** for the alignment).

### Synteny Analysis

To generate synteny map, genes flanking the elephant shark, whale shark and catshark *GnRH* genes were identified by searching flanking sequence in batches of 10 kb non-overlapping fragments against NCBI NR database by BLASTX. Catshark *GnRH1* and *GnRH3* genes were not included in the study because they were found on very short scaffolds that did not contain any other genes. Genes flanking the *GnRH* genes in five osteichthyan species, namely human, chicken (*Gallus gallus*), western clawed frog (*Xenopus tropicalis*), spotted gar (*Lepisosteus oculatus*), and medaka (*Oryzias latipes*), were obtained from Genomicus (Muffato et al., 2010).

### Reverse Transcriptase-Polymerase Chain Reaction (RT-PCR) Amplification

The expression profiles of catshark *GnRH1, GnRH2*, and *GnRH3* genes were examined by RT-PCR, as previously described (Quan et al., 2013). Total RNA was extracted from various tissues, including telencephalon, diencephalon, mesencephalon, cerebellum, brain stem, spinal cord, skeletal muscles, heart, spleen, gills, stomach, duodenum, valvular intestine, liver, kidney, ovary, and testis and purified by using RNeasy Plus Mini kit (Qiagen, Courtaboeuf, France). For each tissue, ∼330 ng of total RNA were reverse transcribed using ImProm-II Reverse Transcription System (Promega, Charbonnières, France). Gene-specific primers of catchark *GnRH1, GnRH2*, and *GnRH3* were designed according to the predicted sequences (**Supplementary Table S1**). PCR amplifications were carried out for 35 cycles (denaturation 94°C, 30 s; annealing between 57 and 60°C depending on the Tm of the primers, 30 s; and extension 72°C, 30 s) and a final extension of 72°C for 7 min. The catshark Egf1 gene was amplified in parallel with specific primers (**Supplementary Table S1**) to verify the quality and quantity of all cDNAs samples, as previously described (Quan et al., 2013). Negative controls were performed without cDNA template. All PCR products were electrophoresed through 2.0% agarose gel and stained with ethidium bromide and then detected under UV light with the ChemiDoc Touch Imaging System (Bio-Rad). Three independent PCR amplifications were performed to check the consistency of amplification.

### Prediction of GAP Three-Dimensional Protein Structure

Secondary protein structures of GAP from the three pre-proGnRH variants present in catshark, elephant shark, and whale shark were modeled using the I-TASSER server, an automated protein-modeling server from the Zhang Lab at the University of Michigan^3^ (Yang et al., 2015). GAP sequences were delimited between the dibasic site for proteolytic processing after the GnRH sequence and the stop codon of the ORF. Only models with C-score between -4 and 2 were considered. Visualization of the predicted three-dimensional (3D) structures, as well as its orientation (N-terminal extreme to the left), were performed using the Jmol software^4^.

## Results

### Structure of GnRH Precursor cDNAs and Genes

#### In Catshark

**FIGURE 1:**
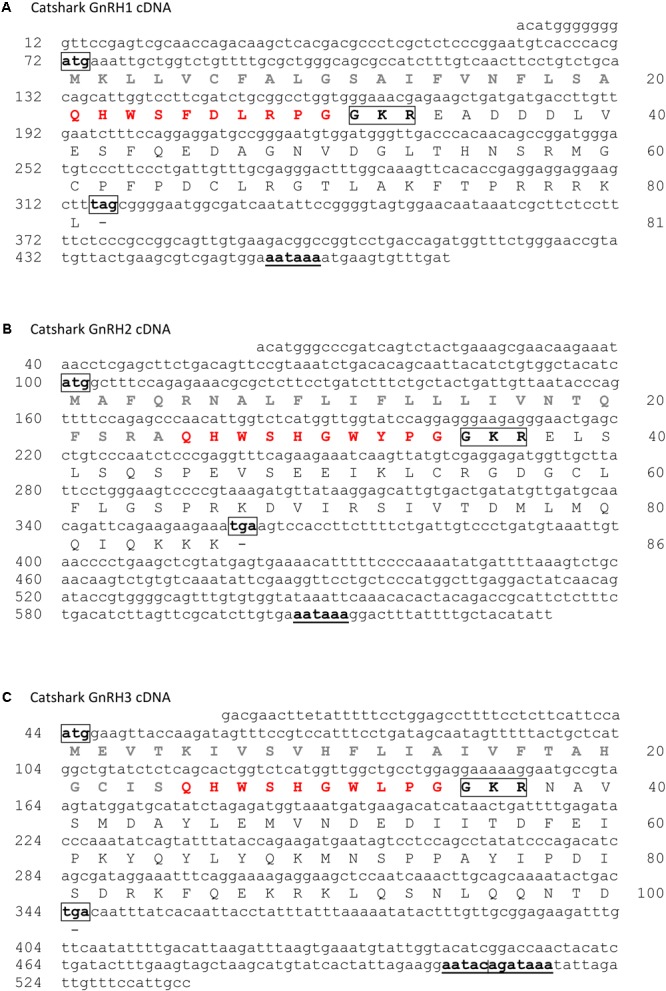
Nucleotide sequence and deduced amino acid sequence of catshark *GnRH1* **(A)**, *GnRH2* **(B)**, and *GnRH3* **(C)** cDNAs. Nucleotides are numbered 5′ to 3′ and amino acids N-terminal to C-terminal from the putative starting methionine. Signal peptides are in gray. GnRH peptide sequences are in red. Potential cleavage sites are boxed. Polyadenylation signals are underlined. Sequences of catshark *GnRH1, GnRH2*, and *GnRH3* cDNAs have been deposited in the GenBank data base under the accession numbers MH468810, MH468811, and MH468812, respectively. ND, not determined.

The catshark genome assembly was searched by TBLASTN using the three little skate GnRH sequences (Roch et al., 2014a) as queries. Three hits were found encoding three putative different GnRH forms. Specific primers were designed from these three sequences and RACE PCR experiments were conducted in order to obtain the complete cDNA sequences (**Figure 1**). The *GnRH1* cDNA sequence contains a 71 bp 5′ terminal untranslated region (UTR) and a 159 bp 3′ terminal UTR with the canonical polyadenylation signal sequence (AATAAA); an ORF of 243 bp encoding a 81 amino acid (aa) protein including a 20 residue potential signal sequence (Nielsen, 2017), the GnRH1 decapeptide, the 3 aa proteolytic cleavage site GKR and the 48 aa GAP (**Figure 1A**). The *GnRH2* cDNA consists of a 99 bp 5′ UTR, a 275 bp 3′ UTR, with the consensus polyadenylation signal; the 258 bp ORF encoding a peptide of 86 aa which comprises a 24 aa putative signal peptide, the decapeptide GnRH2, the 3 aa proteolytic cleavage site GKR, and the 49 aa GAP (**Figure 1B**). The *GnRH3* cDNA sequence contains a 43 bp 5′ UTR and a 194 bp 3′ terminal UTR with a non-canonical polyadenylation signal sequence AATACA or GATAAA (Beaudoing et al., 2000); an ORF of 300 bp encoding a 100 aa protein including a 24 residue potential signal sequence, the GnRH3 decapeptide, the 3 aa proteolytic cleavage site GKR and the 62 aa GAP (**Figure 1C**). Comparison of the cDNAs with genomic sequences revealed that *GnRH1* and *GnRH3* genes are composed of three exons and two introns (**Supplementary Data Sheet S4**). For each gene, exon 1 encodes the 5′ UTR, the signal peptide, the GnRH decapeptide, the amidation/proteolytic processing signal and the N-terminus of the GAP; exon 2 encodes the central portion of the GAP; and exon 3 encodes the C-terminus of the GAP along with the 3′ UTR. *GnRH2* gene possesses an additional exon that contains the first part of the 5′UTR (**Supplementary Data Sheet S4**).

#### In Whale Shark

Using TBLASTN, we searched the whale shark genome assembly using the three catshark GnRH precursor sequences as queries. Three hits were found encoding three putative different GnRH peptides. Where possible, catshark GnRH precursor sequences were used as reference to predict the whale shark GAP-coding exons. For the *GnRH1* gene, only a partial sequence (135 bp) could be retrieved corresponding to exon 1 (**Supplementary Data Sheet S5**). For the two other genes, *GnRH2* and *GnRH3*, the complete coding DNA sequences (CDS) were retrieved split into three exons (**Supplementary Data Sheet S5**). *GnRH2* CDS is a 258 bp sequence composed by a 138 bp exon 1, a 84 bp exon 2 and a 36 bp exon 3, while *GnRH3* CDS is a 285 bp sequence composed by a 141 bp exon 1, a 90 bp exon 2, and a 54 bp exon 3 (**Supplementary Data Sheet S5**). The predicted amino acid GnRH2 and GnRH3 precursor sequences consist of 86 and 95 aa, respectively (**Supplementary Data Sheet S6**).

#### In Elephant Shark

TBLASTN was used to search the elephant shark genome assembly using the three catshark prepro-GnRHs as queries. Three hits were found encoding three putative GnRH precursors, GnRH1a, GnRH1b, both containing the same GnRH1 peptide sequence plus GnRH2. Full length *GnRH1a* and *GnRH2* cDNAs were retrieved from RNA-seq data (GenBank accession number SRA054255) (Venkatesh et al., 2014). Comparison of these cDNAs with the genomic sequences revealed the general organization of *GnRH1a* and *GnRH2* genes. The *GnRH1b* CDS, which could not be retrieved from the RNA-seq data, was predicted using the GnRH1a sequence as a reference. The *GnRH1a* cDNA sequence contains a 486 bp 5′ terminal UTR and a 235 bp 3′ terminal UTR with the canonical polyadenylation signal sequence; an ORF of 252 bp encoding a 84 aa protein including a 24 residue potential signal sequence, the GnRH1 decapeptide, the 3 aa proteolytic cleavage site KKR and the 47 aa GAP (**Supplementary Data Sheet S7**). The *GnRH2* cDNA consists of a 14 bp 5′ UTR, a 293 bp 3′ UTR, and a consensus polyadenylation signal; the 258 bp ORF encoding a peptide of 86 aa which comprises a 24 aa putative signal peptide, the decapeptide GnRH2, the 3 aa proteolytic cleavage site GKR, and the 49 aa GAP (**Supplementary Data Sheet S7**). *GnRH2* gene is composed of three exons and two introns (**Supplementary Data Sheet S8**): exon 1 encodes the 5′ UTR, the signal peptide, the GnRH decapeptide, the amidation/proteolytic processing signal and the N-terminus of the GAP; exon 2 encodes the central portion of the GAP; and exon 3 encodes the C-terminus of the GAP along with the 3′ UTR. *GnRH1a* gene possesses an additional exon that contains the major part of the 5′ UTR (**Supplementary Data Sheet S8**). The predicted *GnRH1b* CDS is a 252 bp sequence composed by a 144 bp exon 1, a 81 bp exon 2, and a 27 bp exon 3 (**Supplementary Data Sheet S8**). The predicted amino acid GnRH1b precursor sequence consists of 84 aa (**Supplementary Data Sheet S7**).

### Comparison of Chondrichthyan Prepro-GnRH Sequences

**FIGURE 2:**
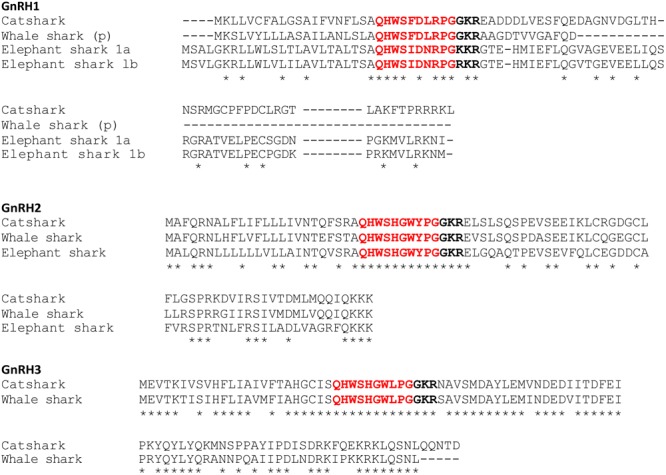
Alignment of the amino acid sequences of prepro-GnRHs characterized in the present study. Note that whale shark prepro-GnRH1 sequence is restricted to that encoded by the first exon. ^∗^Denotes conserved residues. Putative bioactive peptides are in red. Potential cleavage sites are in bold. (p), partial sequence.

As depicted in **Figure 2**, elephant shark prepro-GnRH1a and -1b exhibit very high level of sequence identity (88.1%; see **Supplementary Table S2**). In contrast, catshark and elephant GnRH1 precursors show only low sequence similarities (32.9 to 34.1%, **Supplementary Table S2**). Alignment of the different chondrichthyan prepro-GnRH2s reveal a moderate to high level of sequence identity (54.8% between whale shark and elephant shark, 61.2% between catshark and elephant shark and 80.2% between catshark and whale shark, **Supplementary Table S2**). Finally, catshark and whale shark GnRH3 precursors exhibit a high level of sequence identity (78.9%, **Supplementary Table S2**). Note that the two elephant shark prepro-GnRH1 sequences reported in the present study (GnRH1a and GnRH1b) and that previously published by Roch et al. (2014a) exhibit only 50% of sequence identity (**Supplementary Data Sheet S9**). The differences only concern the region encoded by exons 2 and 3.

### Phylogenetic Analysis

**FIGURE 3:**
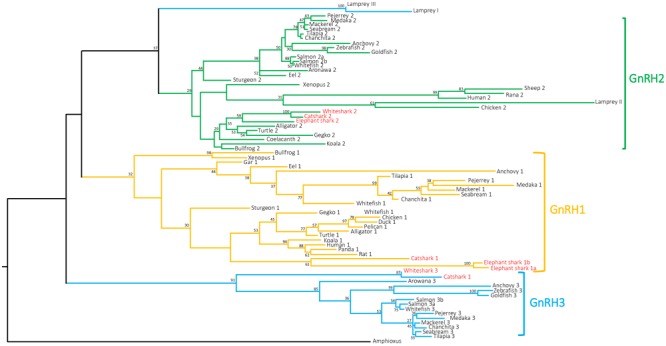
Phylogenetic tree of vertebrate GnRH precursor sequences. Phylogenetic analysis of 73 vertebrate GnRH amino acid sequences was performed using Maximal Likelihood, with 1,000 bootstrap replicates. The number shown at each branch node indicates in percentage the bootstrap value. Only values above 25% are indicated. The tree is rooted with a non-vertebrate chordate (Amphioxus) GnRH sequence used as an outgroup. Sequence references and alignment are given in **Supplementary Data Sheets S1, S2**, respectively.

Based on an amino acid alignment of 74 chordate selected GnRH precursor sequences (73 sequences from vertebrate species including the 8 sequences characterized in the present study plus the amphioxus prepro-GnRH sequence, used as outgroup) (**Supplementary Data Sheet S2**), a phylogenetic tree was constructed using the PhyML method. As shown in **Figure 3**, the phylogenetic tree segregated the gnathostome GnRH sequences into three main clades which correspond to the three paralogs GnRH1, GnRH2, and GnRH3. However, only the GnRH3 group is supported by high bootstrap value (91%) while the other two are extremely low (32% for GnRH1 and 29% for GnRH2). Cartilaginous fish GnRH3 sequences clustered at the base of the GnRH3 clade. For their part, cartilaginous fish GnRH1 and GnRH2 sequences grouped with some tetrapod GnRH1 and GnRH2 sequences, respectively, but not at the root of the corresponding clades. It is to note that, in agreement with its proposed orthology (Gwee et al., 2009; Decatur et al., 2013), lamprey GnRH-II grouped with some of amniote GnRH2 sequences within the GnRH2 clade. In contrast, lamprey GnRH-I and -III sequences, while being possibly related to gnathostome GnRH3 (Decatur et al., 2013) did not cluster with gnathostome GnRH3 sequences.

As for GnRH precursor phylogeny analysis, GAP phylogeny analysis showed large sequence variation with only few nodes being supported by bootstrap values over 50 (**Supplementary Image S1**). Gnathostome GAP1 and GAP3 sequences clustered in two well-supported clades, including chondrichtyan sequences, with bootstrap values over 60, while GAP2 sequences, including catshark, elephant shark, and whale shark GAP2 sequences, did not clustered in a single clade. While lamprey GAPII sequence clustered with some of the gnathostome GAP2 sequences, mainly from tetrapods, lamprey GAPI and GPAIII clustered together at the base of the phylogenetic tree.

### Synteny Analysis

**FIGURE 4:**
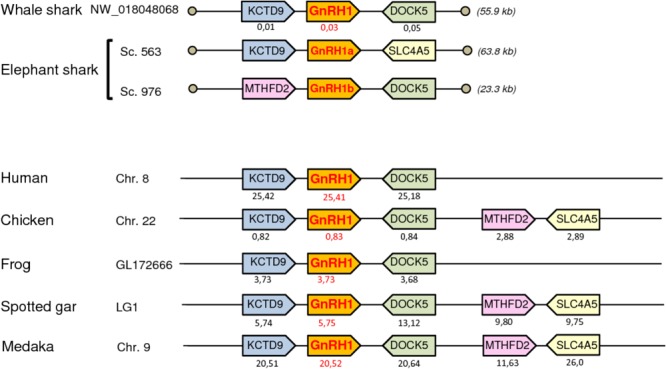
Synteny of genes in the *GnRH1* locus in catshark, whale shark, and elephant shark plus five selected bony vertebrate species (human, chicken, western clawed frog, spotted gar, and medaka). Genes are represented by block arrows. Genes with conserved synteny are colored. Position of the genes (in megabases, Mb) is displayed below each box, according to the Ensembl database. Empty circles indicate the end of scaffolds. The detailed chromosomal locations of genes displayed in this map are included in **Supplementary Table S3**.

**FIGURE 5:**
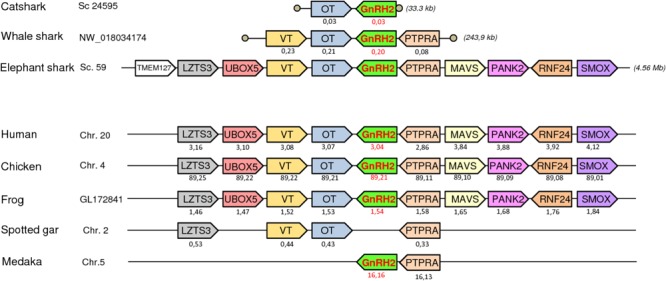
Synteny of genes in the *GnRH2* locus in catshark, whale shark, and elephant shark plus five selected bony vertebrate species (human, chicken, western clawed frog, spotted gar, and medaka). Legends are the same as in the **Figure 4**.

**FIGURE 6:**
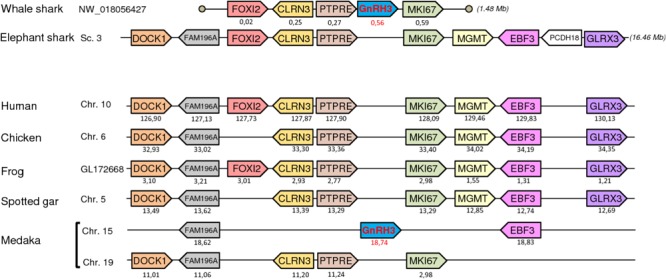
Synteny of genes in the *GnRH3* locus in catshark, whale shark, and elephant shark plus five selected bony vertebrate species (human, chicken, western clawed frog, spotted gar, and medaka). Legends are the same as in the **Figure 4**.

To further resolve the origin and orthology relationships between the different GnRH genes identified in the present study, a synteny analysis was performed. For this purpose, the genomic environment of *GnRH* genes was determined in elephant shark, whale shark, and catsharks and compared to that in five representative osteichthyan species, namely human, chicken, western clawed frog, spotted gar, and medaka. As shown in **Figure 4**, the two genes surrounding *GnRH1* in whale shark, *KCTD9* and *DOCK5*, are also present in all the osteichthyan species examined. In elephant shark, where *GnRH1* is in two copies, one of the copies, *GnRH1a*, is surrounded by *KCTD9* and *SLC4A5*, while the second, *GnRH1b*, is surrounded by *MTHFD2* and *DOCK5*. In chicken and spotted gar, *SLC4A5* and *MTHFD2* can be recognized as two additional genes present at the *GnRH1* locus. In most species examined, *GnRH2* were positioned in genomic regions containing common neighboring genes including *LZTS3, UBOX5, VT, OT, PTPRA, MAVS, PANK2, RNF24*, and *SMOX* (**Figure 5**). Likewise, *GnRH3* reside within a gene cluster that commonly includes *DOCK1, FAM196A, FOXI2, CLRN3, PTPRE, MKI67, MGMT, EBF3*, and *GLRX3*. However, in elephant shark but not whale shark, *GnRH3* gene is missing from this cluster (**Figure 6**). Note that in both catshark and whale shark, *GnRH* scaffolds contained only a small part of these neighboring genes (the closest) due to their very small size (less than 100 kb). In summary, synteny analysis strongly supports the orthology of *GnRH1, GnRH2*, and *GnRH3* genes among all vertebrates, including cartilaginous fish, as previously reported in (Roch et al., 2014a).

### Tissue-Specific Expression of the Catshark *GnRH* Genes

**FIGURE 7:**
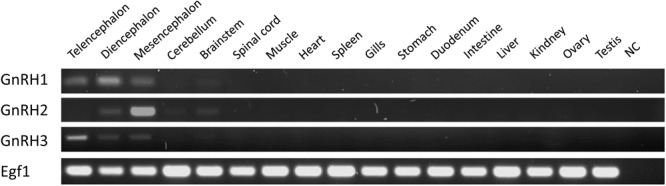
Tissue distribution of catshark *GnRH1, GnRH2*, and *GnRH3* mRNAs using RT-PCR. Parallel amplification of dogfish Egf1 mRNA served as internal control. NC, non-template control.

The distribution of catshark *GnRH1, GnRH2*, and *GnRH3* mRNAs in various tissues was examined by RT-PCR. **Figure 7** shows the results of one representative experiment out of three performed with identical results. Expression of the three *GnRH* genes was detected exclusively in the brain. *GnRH1* mRNA appeared particularly abundant in the diencephalon and at slightly lower levels in the telencephalon and mesencephalon. The *GnRH2* mRNA seemed to be predominant in the mesencephalon but also present in the diencephalon. The *GnRH3* gene appeared to be highly expressed in the telencephalon and to a lesser extent, in the diencephalon and mesencephalon. Note that traces of *GnRH1, GnRH2*, and *GnRH3* mRNAs were also detected in the brainstem.

### Prediction of GAP Three-Dimensional Protein Structure

**FIGURE 8:**
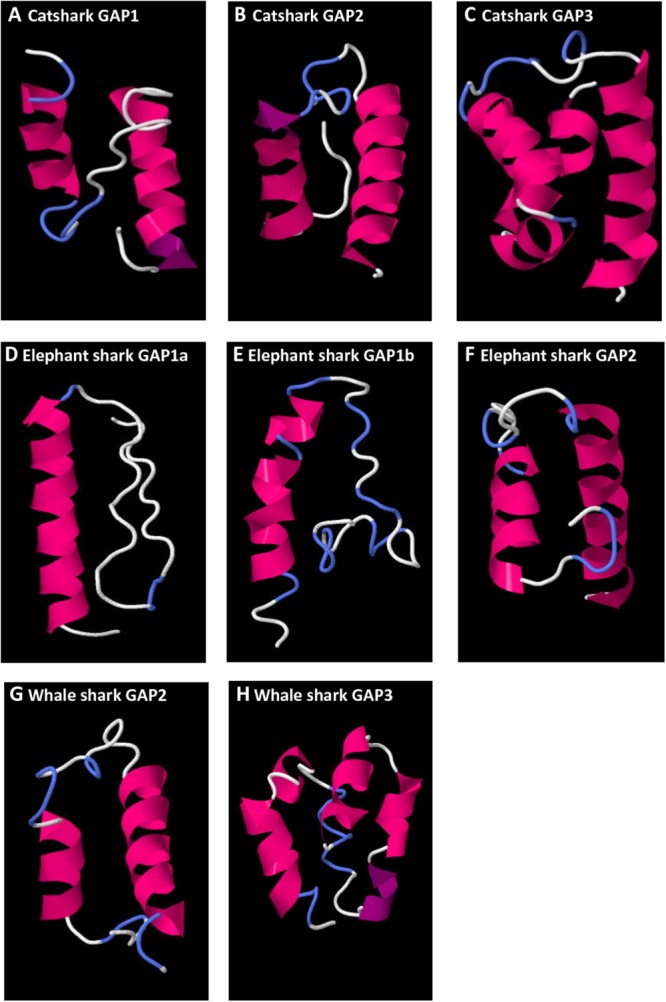
Predicted 3D structure of catshark GAP1 **(A)**, GAP2 **(B)**, and GAP3 **(C)**, elephant shark GAP1a **(D)**, GAP1b **(E)**, and GAP2 **(F)**, whale shark GAP2 **(G)** and GAP3 **(H)**. Models predicted in I-TASSER server with a C-score between 2 and 4 were presented. In all the models, the C-terminal is oriented toward the right. In pink appears α-helix, in violet 3_10_-helix, in white loops, and in blue β-turns.

Secondary protein structures of catshark, elephant shark, and whale shark GAP variants were predicted using the amino acid sequences obtained in the present work (**Figure 8**). For catshark GAP1, a 3D structure characterized by two alpha helices separated by a loop (helix-loop-helix structure, HLH) was obtained (**Figure 8A**). The number of amino acid involved in the loop was 16 aa and the number of aa involved in the N-terminal and C-terminal alpha helices were 10 and 15 aa, respectively. Elephant shark GAP1a (**Figure 8D**) and GAP1b (**Figure 8E**) predicted 3D structures showed a single N-terminal alpha helix composed by 20 and 16 aa, respectively. In the case of catshark, elephant shark, and whale shark GAP2 (**Figures 8B,F,G**), the 3D predicted models presented the classical HLH structure. The loop was formed by 9 aa for catshark and elephant shark GAP2, and 13 aa for whale shark GAP2. The number of aa involved on the N- and C-terminal helices varied from 9 to 20. In the predicted catshark and whale shark GAP3 secondary structures, multiple alpha helices were observed with no typical HLH structure.

## Discussion

The present study reports the identification of three distinct *GnRH* genes in three representative species of cartilaginous fish, namely the catshark *S. canicula*, the whale shark *R. typus* and the elephant shark *C. milii*.

In catshark and whale shark, the three *GnRH* genes corresponded to *GnRH1, GnRH2*, and *GnRH3*, as revealed by both phylogenetic and synteny analyses. The functionality of all genes as well as the validity of their coding sequence was demonstrated in catshark by molecular cloning of the corresponding full-length cDNAs. The occurrence of the same paralogous genes was recently reported in the little skate *L. erinacea* by Roch et al. (2014a). However, the sequences provided in the study were generally incomplete, either reduced to the single exon encoding the mature peptides (*GnRH2*), or extrapolated from sequences generated *in silico* using a gene prediction algorithm (*GnRH1* and *GnRH3*). It is plausible that these predicted sequences were not perfectly correct because they do not exhibit any appreciable similarities when aligned with those from other closely related species (**Supplementary Data Sheet S9**). The existence of only three *GnRH* genes in catshark and little skate seems in apparent contradiction with older studies showing that up to five immunoreactive GnRH forms can be present in these species (Calvin et al., 1992; D’Antonio et al., 1995). One possible explanation for these discrepancies is that some of the GnRH forms detected by HPLC then immunologically characterized may represent enzymatic cleavage products or anomalous elution profiles resulting from interaction with other proteins (Lovejoy et al., 1993; Nock et al., 2011).

In elephant shark, the three *GnRH* genes identified corresponded to two copies of *GnRH1, GnRH1a* and *GnRH1b*, plus *GnRH2*, as revealed by both phylogenetic and synteny analyses. *GnRH1a* and *GnRH2* transcripts could also be characterized, confirming the validity of the gene sequences. Although no *GnRH1b* transcripts could be detected from RNA-seq reads, prediction of the *GnRH1b* sequence was made easier by the fact that *GnRH1a* and *GnRH1b* share a very high level of sequence identity. In contrast, no *GnRH3* gene could be found. Two of the three *GnRH* genes found in elephant shark, *GnRH1b* and *GnRH2* were previously described (Nock et al., 2011; Roch et al., 2014a). In contrast, this is the first time *GnRH1a* has been reported in elephant shark. It is noteworthy that the coding sequence of *GnRH1* predicted by Roch et al. (2014a) strongly differs to that of *GnRH1b* reported here. The inexactness of the Roch’s sequence is undoubtedly due to limitations of the prediction algorithms used, as stated above regarding the skate sequences. Elephant shark *GnRH1a* and *GnRH1b* genes appear to encode the same GnRH1 peptide, QHWSIDNRPG. Presence of these two putative GnRH peptides is consistent with a previous study performed on the closely related species *Chimaera monstrosa* showing the presence of two immunoreactive GnRH forms, namely GnRH1 and GnRH2 (Masini et al., 2008). *GnRH1a* and *GnRH1b* genes can be reasonably viewed to have been generated by segmental duplication of a common ancestral *GnRH1* gene since each are surrounded by two genes, *KCTD9* and *SLC4A5* and *MTHFD2* and *DOCK5*, respectively, that are physically linked in several other species, such as chicken and spotted gar. Although *GnRH1a* and *GnRH1b* genes are located on two distinct contigs, it is likely that they arose by tandem duplication because these contigs are very short (less than 100 kb each) and they contain several genes present in the same genomic environment in osteichthyan species.

In the three chondrichthyan species studied here, as in all other vertebrate species examined so far (Millar, 2003), except in lamprey (Kavanaugh et al., 2008), the sequence of the GnRH2 peptide is totally conserved. In contrast, the sequence of the GnRH1 peptide appears much more variable. Little skate GnRH1 was previously shown to exhibit the same structure to that found in humans and other mammals but elephant shark GnRH1 was proven to be a totally new GnRH1 variant (Roch et al., 2014a). Here we show that catshark and whale shark GnRH1 peptides share the same hitherto unknown primary structure, QHWSFDLRPG. To our knowledge, this new sequence is the thirteenth molecular form of GnRH1 to be described in vertebrates (**Supplementary Table S4**). Finally, the sequence of the GnRH3 peptide found in the two elasmobranch species examined here is identical to that first reported in little skate (Roch et al., 2014a). GnRH3 peptide from cartilaginous fish differs to that from teleosts (generally called salmon GnRH3) by only one residue at position 5 (His in place of Tyr).

The 2R hypothesis predicts that the vertebrate ancestor possessed four *GnRH* genes, from which only three, *GnRH1, GnRH2*, and *GnRH3*, were conserved in the gnathostome ancestor (Okubo and Nagahama, 2008; Kim et al., 2011; Tostivint, 2011; Roch et al., 2014a). It is generally assumed that the putative *GnRH4* gene disappeared shortly after 2R without leaving any trace in current species (Decatur et al., 2013). In the present study, we confirm the absence of *GnRH4* gene in cartilaginous fish. Up to now, *GnRH1, GnRH2*, and *GnRH3* genes were found to coexist primarily in teleost species (Okubo and Nagahama, 2008) and in coelacanth (Roch et al., 2014a; Yun et al., 2015). The occurrence of all these genes in several elasmobranch species, including little skate (Roch et al., 2014a), catshark and whale shark (the present study), indicates preservation in the chondrichthyan ancestor. The *GnRH3* gene is known to have been lost independently multiple times during vertebrate evolution, for instance in tetrapods and in several teleost species (Okubo and Nagahama, 2008; Kim et al., 2011; Tostivint, 2011). Absence of *GnRH3* in elephant shark, already reported by Roch et al. (2014a) also suggests its loss in the holocephalan lineage. To our knowledge, *GnRH1a* and *GnRH1b* found in elephant shark are the only copies of the *GnRH1* gene reported so far in vertebrates. In contrast, two copies of the *GnRH2* and/or *GnRH3* gene were already found in salmonids (Ashihara et al., 1995), probably due to the salmon-specific whole- genome duplication (4R) (Lien et al., 2016) and in sea lamprey (Smith et al., 2013), through tandem duplication. It is likely that duplication of the *GnRH1* gene in the holocephalan lineage occurred recently in evolution since the sequences of the two elephant shark *GnRH1* paralogs are very similar. Despite that, the fact that no *GnRH1b* transcript could be detected in elephant shark strongly suggests that, at least in this species, the *GnRH1b* gene is in course of pseudogenization (Gout et al., 2010; Session et al., 2016).

Little is currently known about the functional and physiological relevance of multiple GnRH peptides in cartilaginous fish compared to other vertebrates (Awruch, 2013). To address this issue, it is essential to clarify their tissue specificity. In bony vertebrates, three major GnRH neuronal systems were recognized (Fernald and White, 1999; Dubois et al., 2002). The first one (expressing GnRH1), called the ventral forebrain GnRH system, is composed of neurons mainly located in the preoptic area of hypothalamus and projecting toward the gonadotropic cells in the pituitary. The second GnRH system (expressing GnRH2), called the midbrain GnRH system, is composed of neurons localized in the midbrain tegmentum near the third ventricle. The third system (expressing GnRH3), called the terminal nerve GnRH system, has so far been studied only in teleosts. Neurons of this system are mainly located in the terminal nerve ganglion near the olfactory bulb. Using RT-PCR, the present study provides information regarding tissue distribution of the three *GnRH* mRNAs in the catshark *S. canicula*. Catshark *GnRH1* mRNA was found both in the forebrain and the midbrain but appeared predominant in the diencephalon suggesting that, as in bony vertebrates, GnRH1 peptide corresponds to the hypophysiotropic form of GnRH in catshark. Catshark *GnRH2* mRNA was primarily found in the mesencephalon while catshark *GnRH3* mRNA was mainly detected in the telencephalon. These results strongly suggest that both the midbrain and the terminal nerve GnRH systems also occurred in cartilaginous fish. *In situ* hybridization experiments will be needed to further support this view.

Prediction of GAP variants 3D structure was previously reported for different vertebrate groups (Pérez Sirkin et al., 2017) where a chondrichthyan GAP2 sequence (elephant shark) was analyzed. However, as there was no available complete sequences for chondrichthyes pre-proGnRH1 and pre-proGnRH3, their corresponding 3D GAP structures could not be analyzed in that work. In the present study, complete sequences of pre-proGnRH1 and -3 for chondrichtyan species were presented for the first time. Predicted catshark GAP1 3D structure revealed an HLH structure in concordance with the GAP1 3D structure observed throughout the vertebrate lineage (Pérez Sirkin et al., 2017). Although it has been proposed that GAP is co-secreted with GnRH (Clarke et al., 1987), and may exert some hypophysiotropic actions in mammals (Nikolics et al., 1985), the possible biological function of GAP1 is still largely unknown and controversed. The result obtained in the present work adds another group for this striking conservation in GAP1 3D HLH structure, supporting the hypothesis that this peptide could present hypophysiotropic biological functions (Nikolics et al., 1985). In contrast, elephant shark GAP1a and GAP1b 3D predicted models appear to have lost this conserved HLH structure showing a single N-terminal alpha helix. Chondrichthyan GAP2 also presented an HLH structure with great similarity to the one predicted in most osteichthyan GAP2 (Nikolics et al., 1985). The length of the GAP2 sequences, as well as the helices and loop length, were also highly conserved. Finally, no typical 3D HLH structure was observed for GAP3 in chondrichthyans, as previously shown for teleosts GAP3 (Pérez Sirkin et al., 2017). These results in chondrichthyans suggest that the HLH 3D structure seen in GAP1 and GAP2, which may convey their biological activity, would represent an ancestral feature largely conserved among gnathostome radiation.

## Conclusion

**FIGURE 9:**
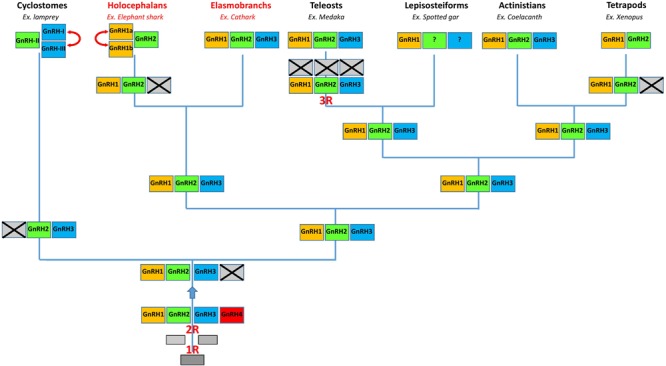
A proposed evolutionary scheme for the evolution of the *GnRH* gene family in vertebrates with a special emphasis on chondrichthyan species (in red). The names of the different paralogous genes are given in the boxes. Crossed-out boxes represent lost genes. Double-headed arrows represent local duplications. R, rounds of whole genome duplication.

We revealed the *GnRH* gene repertoire in three representative species of cartilaginous fish. We showed that in catshark and whale shark the *GnRH* genes correspond to *GnRH1, GnRH2*, and *GnRH3*, while in elephant shark they correspond to *GnRH1a* and *GnRH1b*, two copies of the *GnRH1* gene, plus *GnRH2*. Taken together, our results indicate that cartilaginous fish inherited the complete set of *GnRH* genes already present in the vertebrate ancestor (**Figure 9**). This set was then entirely conserved in elasmobranchs while gene losses and duplications could occur in the holocephalan lineage (**Figure 9**). Our results also suggest that the three GnRH neuronal systems previously described in bony vertebrates are also conserved in cartilaginous fish. Finally, they show that the HLH 3D structure of GAP1 and GAP2 is largely conserved among gnathostomes.

## Author Contributions

HT conceived and designed the experiments. A-LG, DPS, A-GL, CDF, and HT performed the experiments. A-LG, CDF, DPS, A-GL, PV, SD, BV, and HT analyzed the data. B-HT, SM, and BV contributed reagents, materials, and analysis tools. HT wrote the paper. All authors approved the final version of the manuscript.

## Conflict of Interest Statement

The authors declare that the research was conducted in the absence of any commercial or financial relationships that could be construed as a potential conflict of interest.

## Footnotes

1 http://blast.ncbi.nlm.nih.gov/Blast.cgi

2 http://www.ebi.ac.uk/Tools/msa/mafft/

3 http://zhanglab.ccmb.med.umich.edu/I-TASSER/

4 http://jmol.sourceforge.net/index.en.html

## Supplementary Material

IMAGE S1 Phylogenetic tree of vertebrate GAP sequences. Phylogenetic analysis of 75 vertebrate GAP amino acid sequences was performed using Maximal Likelihood, with 1,000 bootstrap replicates. The number shown at each branch node indicates in percentage the bootstrap value. Only values above 25% are indicated. The tree is rooted with a non-vertebrate chordate (Amphioxus) GAP sequence used as an outgroup. Sequence references and alignment are given in **Supplementary Data Sheets S1, S3**, respectively.

TABLE S1 Sequences of the oligonucleotides used for PCR amplifications.

TABLE S2 Pair-wise comparison of full-length GnRH protein sequences from catshark, *Scyliorhinus canicula*, whale shark, *Rhincodon typus*, and elephant shark, *Callorhinchus milii*. Values represent percentage of amino acid identity.

TABLE S3 Chromosomal locations of gene orthologs in the synteny analysis of the *GnRH* loci. All human gene names in the table are approved HGNC symbols and the genes from other species have been given the name of their human orthologs. Chromosomal locations of genes are expressed in bp. Blanks represent genes that have not been found in the current genome databases. Non-syntenic genes are in gray.

TABLE S4 Primary structure of GnRH isoforms in vertebrates. Conserved residues are boxed. Amino acids changed compared with chicken GnRH2 are colored: in blue for GnRH1, orange for GnRH2 and green for GnRH3. Chondrichthyan species are in red.

DATA SHEET S1 Protein sequences of prepro-GnRHs used for phylogenetic analysis and their accession numbers. Sequences revealed in the present study are in red. GnRH and GAP sequences are highlighted in green and yellow, respectively.

DATA SHEET S2 Amino acid sequence alignment of the GnRH family used for the phylogenetic analysis. The alignment was made using MAFFT and then manually optimized.

DATA SHEET S3 Amino acid sequence alignment of the GAP family used for the phylogenetic analysis. The alignment was performed using Muscle and then manually optimized.

DATA SHEET S4 Sequence of catshark *GnRH1* **(A)**, *GnRH2* **(B)**, and *GnRH3* **(C)** genes, inferred from the cDNA sequences given in **Figure 1**. Exons are indicated by colored boxed. The exon/intron boundary consensus sequences (GT/AG) are highlighted in red. Initiation (ATG) and stop codons are boxed. Polyadenylation signals are underlined. The identity of the scaffolds containing the sequences are indicated.

DATA SHEET S5 Predicted sequence of whale shark *GnRH1* **(A)**, *GnRH2* **(B)**, and *GnRH3* **(C)** genes, inferred from *GnRH* coding sequence given in **Supplementary Data Sheet S6**. Legends are the same as in the **Supplementary Data Sheet S4**.

DATA SHEET S6 Nucleotide sequence and deduced amino acid sequence of whale shark predicted *GnRH1* **(A)**, *GnRH2* **(B)**, and *GnRH3* **(C)** coding sequences. Legends are the same as in the **Figure 1**.

DATA SHEET S7 Nucleotide sequence and deduced amino acid sequence of elephant shark *GnRH1a* cDNA **(A)**, predicted *GnRH1b* coding sequence **(B)**, and *GnRH2* cDNA **(C)**. Legends are the same as in the **Figure 1**.

DATA SHEET S8 Nucleotide sequences of elephant shark *GnRH1a* **(A)**, *GnRH1b* **(B)**, and *GnRH2* **(C)** genes. *GnRH1a* and *GnRH2* are inferred from the cDNA sequences given in **Supplementary Data Sheet S7**. *GnRH1b* is inferred from the predicted *GnRH1b* coding sequence given in the same figure. Legends are the same as in the **Supplementary Data Sheet S4**.

DATA SHEET S9 Alignment of the amino acid sequences of chondrichthyan prepro-GnRHs. This figure includes the sequences already displayed in **Figure 2** plus the predicted skate GnRHs and elephant shark GnRH1a sequences previously published in (Roch et al., 2014a). Legends are the same as in the **Figure 2**.
