# Supplementary figures and images for: Characterization of Gonadotropin-Releasing Hormone (GnRH) Genes From Cartilaginous Fish: Evolutionary Perspectives
Source: Front Neurosci. 2018 Sep 6;12:607. doi: 10.3389/fnins.2018.00607 (PMC6135963; doi:10.3389/fnins.2018.00607)

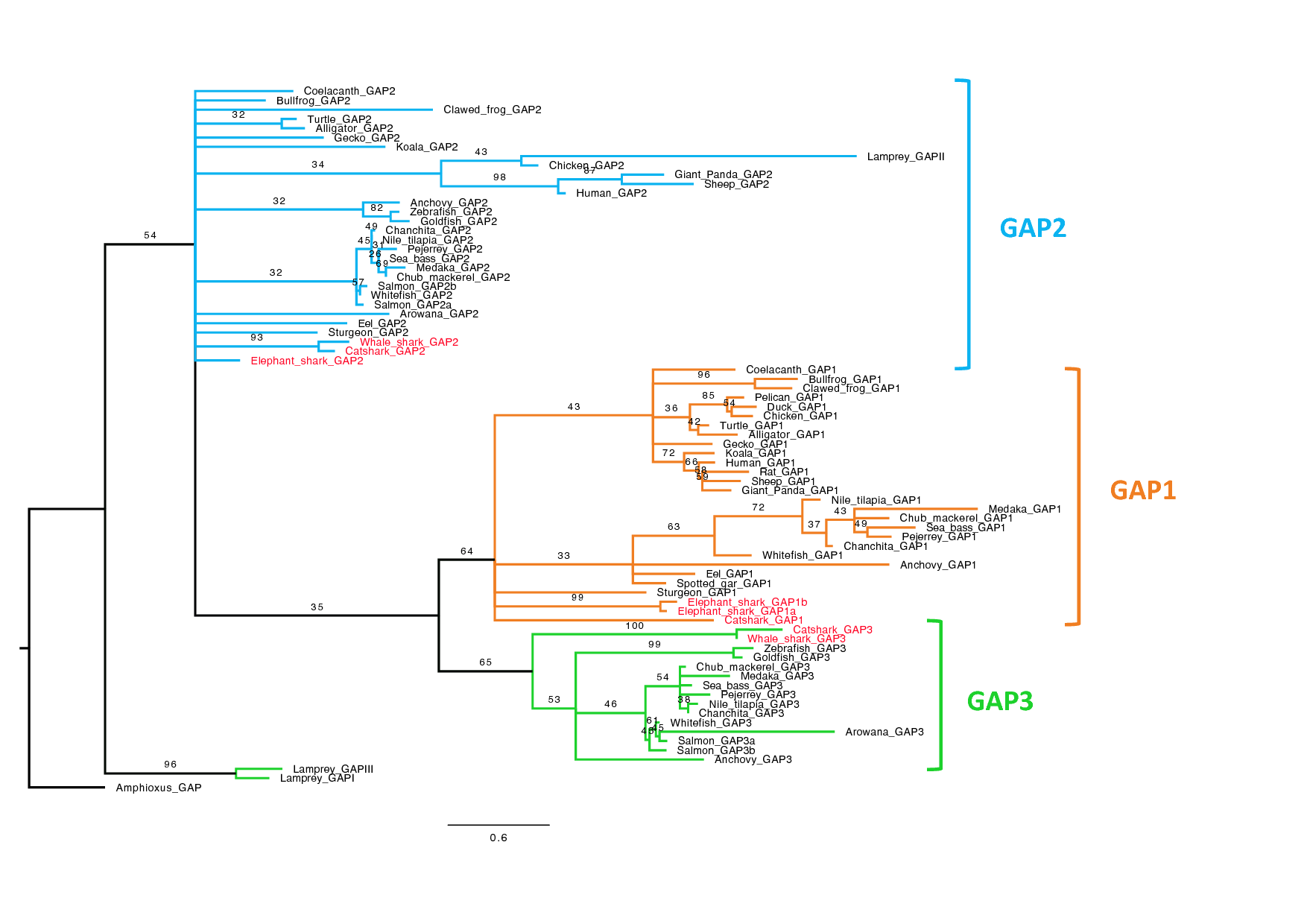

Supplement: IMAGE S1 — Phylogenetic tree of vertebrate GAP sequences. Phylogenetic analysis of 75 vertebrate GAP amino acid sequences was performed using Maximal Likelihood, with 1,000 bootstrap replicates. The number shown at each branch node indicates in percentage the bootstrap value. Only values above 25% are indicated. The tree is rooted with a non-vertebrate chordate (Amphioxus) GAP sequence used as an outgroup. Sequence references and alignment are given in Supplementary Data Sheets S1, S3, respectively. [file Image_1.TIFF]
