# Supplementary material for: Characterization of Gonadotropin-Releasing Hormone (GnRH) Genes From Cartilaginous Fish: Evolutionary Perspectives
Source: Front Neurosci. 2018 Sep 6;12:607. doi: 10.3389/fnins.2018.00607 (PMC6135963; doi:10.3389/fnins.2018.00607)
Supplement: TABLE S1 — Sequences of the oligonucleotides used for PCR amplifications. [file Table_1.DOCX]

| Molecular cloning | | |
| --- | --- | --- |
| GnRH1 | GnRH1 For | aattgctggtctgttttgcg |
|  | GnRH1 For Nest | cagcgccatctttgtcaact |
|  | GnRH1 Rev | gcagatcgaaggaccaatgc |
|  | GnRH1 Rev Nest | atcgaaggaccaatgctgtg |
| GnRH2 | GnRH2 For | gaaacgcgctcttcctgatc |
|  | GnRH2 For Nest | cgcgctcttcctgatctttc |
|  | GnRH2 Rev | atgttgggctctggaaaactg |
|  | GnRH2 Rev Nest | gggctctggaaaactgggt |
| GnRH3 | GnRH3 For | ttccgtccatttcctgatagc |
|  | GnRH3 For Nest | TCTCAGCACTGGTCTCATGGTTGG |
|  | GnRH3 Rev | TGTCTGGGATATAGGCTGGAGGA |
|  | GnRH3 Rev Nest | Aaccatgagaccagtgctga |
| Tissue distribution analysis | | |
| GnRH1 | GnRH1 For | cagcgccatctttgtcaact |
|  | GnRH1 Rev | atccacattcccggcatcctc |
| GnRH2 | GnRH2 For | acctcgagcttctgacagt |
|  | GnRH2 Rev | atgttgggctctggaaaactg |
| GnRH3 | GnRH3 For | tctctcagcactggtctcatg |
|  | GnRH3 Rev | TGTCTGGGATATAGGCTGGAGGA |
| Egf1 | Egf1 For | CTTTCGCTCACCTACCCAAG |
|  | Egf1 Rev | GGTCAAACTGCTCCCAGAAG |
