# Supplementary material for: Characterization of Gonadotropin-Releasing Hormone (GnRH) Genes From Cartilaginous Fish: Evolutionary Perspectives
Source: Front Neurosci. 2018 Sep 6;12:607. doi: 10.3389/fnins.2018.00607 (PMC6135963; doi:10.3389/fnins.2018.00607)
Supplement: TABLE S2 — Pair-wise comparison of full-length GnRH protein sequences from catshark, Scyliorhinus canicula, whale shark, Rhincodon typus, and elephant shark, Callorhinchus milii. Values represent percentage of amino acid identity. [file Table_2.DOCX]

| **GnRH1** | Csh | Esh_Roch | Esh 1a | Esh 1b |
| --- | --- | --- | --- | --- |
| Catshark |  | 28.57 | 34.18 | 32.91 |
| Elephant shark_Roch |  |  | 49.12 | 50.88 |
| Elephant shark 1a |  |  |  | 88.10 |
| Elephantshark 1b |  |  |  |  |

| **GnRH2** | Csh | Wsh | Esh |
| --- | --- | --- | --- |
| Catshark |  | 80.23 | 61.29 |
| Whale shark |  |  | 54.84 |
| Elephant shar |  |  |  |

| **GnRH3** | Csh | Wsh |
| --- | --- | --- |
| Catshark |  | 78.95 |
| Whale shark |  |  |
