# Supplementary material for: Characterization of Gonadotropin-Releasing Hormone (GnRH) Genes From Cartilaginous Fish: Evolutionary Perspectives
Source: Front Neurosci. 2018 Sep 6;12:607. doi: 10.3389/fnins.2018.00607 (PMC6135963; doi:10.3389/fnins.2018.00607)
Supplement: TABLE S4 — Primary structure of GnRH isoforms in vertebrates. Conserved residues are boxed. Amino acids changed compared with chicken GnRH2 are colored: in blue for GnRH1, orange for GnRH2 and green for GnRH3. Chondrichthyan species are in red. [file Table_4.DOCX]

**GnRH1 Mammals/Skate QHWSYGLRPG**

**GnRH1 Guinea pig QYWSYGVRPG**

**GnRH1 Chicken QHWSYGLQPG**

**GnRH1 Frog QHWSYGLWPG**

**GnRH1 Coealacanth QYWSYDLRPG**

**GnRH1 Sea bream QHWSYGLSPG**

**GnRH1 Pejerrey/Medaka QHWSFGLSPG**

**GnRH1 Herring QHWSHGLSPG**

**GnRH1 Catfish QHWSHGLNPG**

**GnRH1 Whitefish QHWSYGMNPG**

**GnRH1 Stickleback QHWSYGLNPG**

**GnRH1 Elephant shark QHWSIDNRPG**

**GnRH1 Catshark/Whaleshark QHWSFDLRPG**

**GnRH2 Chicken QHWSHGWYPG**

**GnRH2 Lamprey-II QHWSHGWFPG**

**GnRH3 Salmon QHWSYQWLPG**

**GnRH3 Dogfish/catshark/whaleshark/skate QHWSHQWLPG**

**GnRH3 Lamprey-I QHWSHDWKPG**

**GnRH3 Lamprey-III QHYSLEWKPG**
