# Supplementary material for: Characterization of Gonadotropin-Releasing Hormone (GnRH) Genes From Cartilaginous Fish: Evolutionary Perspectives
Source: Front Neurosci. 2018 Sep 6;12:607. doi: 10.3389/fnins.2018.00607 (PMC6135963; doi:10.3389/fnins.2018.00607)
Supplement: DATA SHEET S1 — Protein sequences of prepro-GnRHs used for phylogenetic analysis and their accession numbers. Sequences revealed in the present study are in red. GnRH and GAP sequences are highlighted in green and yellow, respectively. [file Data_Sheet_1.DOCX]

**Pre-pro-GnRH1**

>Human1 (NP_000816.4) *Homo sapiens* MCLRMKPIQKLLAGLILLTWCVEGCSSQHWSYGLRPGGKRDAENLIDSFQEIVKEVGQLAETQRFECTTHQPRSPLRDLKGALESLIEEETGQKKI

>Rat1 (NP_036899.1) *Rattus norvegicus* METIPKLMAAVVLLTVCLEGCSSQHWSYGLRPGGKRNTEHLVDSFQEMGKEEDQMAEPQNFECTVHWPRSPLRDLRGALERLIEEEAGQKKM

>Koala1 (AJT59745.1) *Phascolarctos cinereus*

MELTQKLVAGCLLLTVCVTISSGQHWSYGLRPGGKRDADNLIDSFQEMADEGNQLAEPQRFECTIHQPRSPLRDLKGVLASLIEGEAGRKKA

>Panda1 (XP_002914474.2) *Ailuropoda melanoleuca*

MFLRMEPIPKLIAGLLLLTLCVVGCSGQHWSYGLRPGGKRNAEKLIDSFQEIAKELDQGAEPQHLECTIHQPQTPLRDLKGALESLIEEENGQKRI

>Chicken1 (NP_001074346.1) *Gallus gallus* MEKSRKILVGVLLETASVAICLAQHWSYGLQPGGKRNAENLVESFQEIANEMESLGEGQKAECPGSYQHPRLSDLKETMASLIEGEARRKEI

>Duck1 (XP_012959602.1) *Anas platyrhynchos*

MQKSRKAFVGILLFIVSVEICLAQHWSYGLQPGGKRNVDNLGELFQEIANDMEKIGEVQKTECPGSYQHPQFTDLKEAMASLIEGEARRKKI

>Pelican1 (XP_009482176.1) *Pelecanus crispus* MEKSRKIFVSILLEVMSVEICLAQHWSYGLQPGGKRNAENLVESFQETANEMEKSGEVQKTECPGLRQHSRFSDLKEAMESLIEGEARRKKI

>Alligator1 (KQL69429.1) *Alligator mississippiensis* MYTSAASKNCRMQKTRKVFVSLLLLILSVDICLAQHWSYGLQPGGKRNAENVVESFQQMASDMEKFGEMQQFECSSGPHQPSKLSDLKKALASLIEGEAGRKKT

>Gecko1 (ABB89899.1) *Eublepharis macularius*

MGSLLTSFLULLCVAIGSAQHWSYGLQPGGKRDAENLIESFQEIANEVDKVGELQHLECTASQQRPTLQGLKGALASLIDRETGQKKI

>Turtle1 (XP_005285222.1) *Chrysemys picta bellii* MEKTRKLEVRELMFILSVEICLAQHWSYGLQPGGKRDAENLVESFQEIASEMEKIGEMQHFECTGPHQRSMLSGLKGALASLIEGDAGRKKI

>Xenopus1 (NP_001107165.1) *Xenopus tropicalis*

MKAISTYALLLLVLLFSAHVGHAQHWSYGLRPGGKRDAESLQDMYPETPNEVPLFPELERLECSVPQSRLNVLRGALMSWLDGENRKKI

>Bullfrog1 (090V63.1) *Rana catesbeiana*

MSRHVTVVLLLAIVLLLSSHMIHGQHWSYGLRPGGKREVESLQESYAEVPNEVSFTELQHLECSIPQNRISLVRDALMNWLEGENARKKI

>Sturgeon1 (AGK30598.1) *Acipenser sinensis* MAVSRGAFVWLLLSLMAVSEVCYGQHWSYGLRPGGKRETETLLDTLQEIADIEKLDTGDHSECALSSQRSQLSDLKGVLARLVGGESARKKI

>Gar1 (XP_006625369.1) *Lepisosteus oculatus* MKAQKSSLFWLVVAMTLVTQACSQHWSYGLRPGGKREVESLQDTLQDIAEEVRKLDAIRQPGCADVSPQSRLSSLRELLASLAEEERGRKNI

>Eel1 (ADD92012.1) *Anguilla anguilla* MADKSALLWLGLAVALVCQGCCQHWSYGLRPGGKRGADSLQDTLQDIIEELQKLDTSSLPSCNDLSPHITLSSLKEILANLADRETGRKNI

>Medaka1 (BAB16303.1) *Oryzias latipes*

MVVKTWMPWLLVSSVLSQGCCQHWSFGLSPGGKRELKYFPNTLE NQIRLLNSNTPCSDLSH LEESSLAKIYR I KGLL GSVTEAKNGYRTYK

>Seabream1 (AAF62898.1) *Dicentrarchus labrax*

MAAQTFALRLLLVGTLLGTLLGQGCCQHWSYGLSPGGKRELDGLSETLGNQIVGSFPHVATPCRVLGCAEESPFPKIYRMKGFLDAVTDRENGNRTYKK

>Chanchita1 (AKG03158.1) *Cichlasoma dimerus* MHRSFFLRMAAKILALWLLLAGTAFPQGCCQHWSYGLSPGGKRDLDTESDALGNMVEEFPRVEAPCSVEGCAEESPFAKMYRVKGLVGSVTERENGHRTYKK

>Whitefish1 (AAP57221.1) *Coregonus clupeaformis* MEEKKVLLLLLLLVVALVSQGCCQHWSYGMNPGGKRATGSLSDTQDNMAEDLLKIDPSCSLFGCADVSPHAKMYRLRALLASLADRQSGLNNI

>Anchovy1 (AF070217.1) *Engraulis japonicas* MRSKGALVCLLLVTAAVLQCSSQHWSHGLSPGGKREADSPSESQVMEGLPRRGGARCGSDTRDAANQERPSTLEQLISLMSRANEVYD

>Mackerel1 (ADP89591.1) Scomber *japonicus* MHRRMAMQTLALWLLLLGSVVPQVCCQHWSYGLSPGGKRELDSLSDTMDDVVEGFPQVDTPCSFLGCAEESPFAKIYRMKGLFGSVTNRENGHRNYKK

>Tilapia1 (BAC56849.1) *Oreochromis niloticus* MAAKILALWLLLAGTVFPQGCCQHWSYGLSPGGKRDLDNESDTLGNMVEEFPRVEAPCSVEGCAEESPFAKMYRVKGLLASLAEGKTDTGHSRNERFL

>Pejerrey1 (AAU94309.2) *Odontesthes bonariensis* MAVRTWALWPLLVGSVLLQVTCQHWSFGLSPGGKRDLDTFSDTLDNVVEGFPHMDAPCRVVGCADESPFAKIYRMKGFLGGVTDRENGRRVYKK

>Catshark1 (MH468810) *Scyliorhinus canicula* MKLLVCFALGSAIFVNELSAQHWSFDLRPGGKREADDDLVESFQEDAGNVDGLTHNSRMGCPFPDCLRGTLAKFTPRRRKL

>Elephant shark1a (SRA054255) *Callorhinchus milli* MSALGKRLLWLSLTLAVLTALTSAQHWSIDNRPGKKRGTEHMIEFLQGVAGEVEELIQSRGRATVELPECSGDNPGKMVLRKNI

>Elephant shark1b (ND) *Callorhinchus milli* MSVLGKRLLWLVLILAVLTALTSAQHWSIDNRPGRKRGTEHMIEFLQGVTGEVEELLQSRGRATVELPECPGDKPRKMVLRKNM

**Pre-pro-GnRH2**

>Medaka2 (BAB16300.1) *Oryzias latipes* MSRLVLLLGVLLYVGAQLSQAQHWSHGWYPGGKRELDSFEVSEEMKLCETGECSYMRPQRRSFLRNIVLDALARELQKRK

>Goldfish2 (042471.1) *Carassius auratus*

MVHICRLFVVMGMLMFLSVQFASSQHWSHGWYPGGKREIDVYDPSEVSEEIKLCNAGKCSFLIPQGRNILKTILLDALTRDFQKRK

>Zebrafish2 (AAU43784.1) *Danio rerio*

MVLVCRLLLVMGLMLCLSAQLSSAQHWSHGWYPGGKREIDLYDTSEVSEEVKLCEAGKCSYLRPQGRNILKTILLDALIRDFQKRK

>Chanchita2 (ADV31310.1) *Cichlasoma dimerus*

MCVSRLVLLLGLLLCVGAQLSFAQHWSHGWYPGGKRELDSFGTSEISEEIKLCEAGECSYLRPQRRGILRNILLDALARELQKRK

>Seabream2 (Q9IA08.1) *Dicentrarchus labrax* MCVSRLVLLFGLLLCVGAQLSNAQHWSHGWYPGGKRELDSEGTSEISEEIKLCEAGECSYLRPQRRSVLRNIILDALARELQKRK

>Whitefish2 (AAP57219.1) *Coregonus clupeaformis*

MVSVARLVFMLGLLLCLGAQLSSSQHWSHGWYPGGKRELDSFTTSEISEEIKLCEAGECSYLRPQRRNILKN**I**LLDALAREFEKRK

>Salmon2a (XP_013987737.1) *Salmo salar*

MVSVARLVFMLGLLLCLGAQLSSSQHWSHGWYPGGKRELDSFTTSEISEEIKLCEAGECSYLRPQRRNILKNIILDVLAREFQKRK

>Salmon2b (NP_001134611.1) Salmo *salar*

MVSVARLVLMLGLLLCLGAQLSSSQHWSHGWYPGGKRELDSFTTSEISEEIKLCEAGECSYLRPQRRNILRNILLDALAREFEKIK

>Anchovy2 (AF070218.1 GnRH2) *Engraulis japonicus* MACGYRWVLLAAVLLFLGVELSGAQHWSHGWYPGGKRDVDTENSAQVSEEIKLCEAGECSYLRPQRRNLLKSILLEALTREFQRRK

>Arowana2 (BAB72183.1) *Scleropages jardinii* MVCVGRLTLLLGILLCSGAQLSCSQHWSHGWYPGGKRELNSLTASEVSGKIKLCEDRKCSYLRPQQKNILTILVDASTREFRGKRK

>Mackerel2 (ADP89592.1) *Scomber japonicus* MCVSRLVLLLGLLLCVGAQLSNAQHWSHGWYPGGKRELDSFGTPEISEEIKLCEAGECSYLRPQRRSFLRNIILDALARELQKRK

>Tilapia2 (BAC56850.1) *Oreochromis niloticus* MCVSRLALLLGLLLCVGAQLSFAQHWSHGWYPGGKRELDSEGTSEISEEIKLCEAGECSYLRPQRRSILRNILLDALARELQKRK

>Pejerrey2 (AAU94307.1) *Odontesthes bonariensis* MSRLVLLLVLLLYVGAQLSYAQHWSHGWYPGGKRELDSFSTSEISEENKLCEAGECSYLRPQRQNVLRNIVLDALARELQKRK

>Eel2 (ADD92005.1) *Anguilla anguilla* MVNTGRLVLILGVLLCLGAQLSLCQHWSHGWYPGGKRELDSLTTAEVLDEIKLCDGGECSYLRPQRKSLLKNILLDALAREFQRKRK

>Sturgeon2 (AGK30597.1) *Acipenser sinensis* MACQGKLLVLLAVLLALSAQLSSGQHWSHGWYPGGKRELEGLQSPEDSDEVKLCDGDECSYLRHPRKNILRSILADMLTRQMQRKK

>Coelacanth2 (ABZ04537.1) *Latimeria menadoensis* MACQRSLVILLEVLLAVSIQLCSTQHWSHGWYPGGKRELAIPQTPEVSEEIKLCDGEECTYLRSPRKSILKEILADIIAWQIQKKK

>Xenopus2 (NP_001107550.1) *Xenopus tropicalis* MACQGHLVLLLIVLFAFSTHLSNAQHWSHGWYPGGKRQLDTRSIPEISDELKPCEGESCDYPMNEMSILKGLLTRFLFPRERQRK

>Bullfrog2 (AAL05971.1) *Rana catesbeiana* MACQRHLLFLLLVLFAVSTQLSHGQHWSHGWYPGGKRELDMPASPEVSEEIKLCEGEECAYLRNPRKNLLKNILADVLARQLQKK

>Gegko2 (BAC99084.1) *Eublepharis macularius* MACHRPLLLFLCIMIIATIHLSKAQHWSHGWYPGGKREVDLSQSPEVSEDIKLCDGDDCTYLKIPREKIVTSLLADLLAKHLQKKK

>Turtle2 (XP_006139278.1) *Pelodiscus sinensis* MACQRPFLLLLLVVLAVSTHLSRAQHWSHGWYPGGKRELDLSQAPEASEEIKLCDGEACAYLRSPRKTIVNTLLADLLARQLQKKK

>Alligator2 (XP_006020952.1) *Alligator sinensis* MACPRSLLLLLVLLLAIGVPLARAQHWSHGWYPGGKRELDLSQAPQASEEIKICGGEECAYVRSPRMNVVKTLLADMLARQLQKKK

>Chicken2 (BAE80719.1) *Gallus gallus* MAPGGCLLLALLLLAGTAQGQHWSHGWYPGGKRDLSAPQVPAALRPCPTPPCRPLPPMPSTLRAALWRPLEAALRQPH

>Koala2 (AJT59746.1) *Phascolarctos cinereus* MSCLRPLLLLGLLVLWTQISYAQHWSHGWYPGGKRALDEIPGLEASEEGKLWDGGERSLLKTLLADVLAQQQQK>Human2 (NP_847901.1) *Homo sapiens*

MASSRRGLLLLLLLTAHLGPSEAQHWSHGWYPGGKRALSSAQDPQNALRPPAGSPVQTAHGLPSDALAPLDDSMPWEGRTTAQWSLHRKRHLARTLLTAAREPRPAPPSSNKV

>Panda2 (XP 011222695.1) *Ailuropoda melanoleuca* MASCRLGFLUILLTVHPGSLKAQHWSHGWYPGGKRASSSAQHPQRAPRLLGRVLGTAASSPDQAAHNLPSNALAPPENSVPWEARTTGWWPLRQKQHLVKTLLTGRRASRPVALQ

>Sheep2 (XP_012044160.1) *Ovis aries* MASFGLGLLLLLLLLLTTHPGPSKAQHWSHSXYPGGKRASSLPRDPQHPPRPPAQSPGQIAHTLPSDALAWPEDSVPWKSRTMTRWFLRGKQHLVQTLLVSKVEGPHPWPLQGQLRTEVVGG

>Elephantshark2 (XP_007896209.1) *Callorhinchus milii*

MALQRNLLLLLLVLLAINTQVSRAQHWSHGWYPGGKRELGQAQTPEVSEVFQLCEGDDCAFVRSPRTNLFRSILADLVAGRFQKKK

>Catshark2 (MH468811) *Scyliorhinus canicula*

MAFQRNALFLIFLLLIVNTQFSRAQHWSHGWYPGGKRELSLSQSPEVSEEIKLCRGDGCLFLGSPRKDVIRSIVTDMLMQQIQKKK

>Whale shark2 (ND) *Rhincodon typus*

MAFQRNLHFLVFLLLIVNTEFSTAQHWSHGWYPGGKREVSLSQSPDASEEIKLCQGEGCLLLRSPRRGIIRSIVMDMLVQQIQKKK

>Lampreyll (ABE66462.1) *Petromyzon marinus*

MNERIDCCLSLALWRMGRASLSLVLILWLLTAPPASLGQHWSHGWFPGGKRGVQEPPRASYENVSPSDGSPFTPVSSGLQVADWHVVCSRSPNGFSGCAMCCSPGCPTFLSQVLEASLGT

**Pre-pro-GnRH3**

>Goldfish3 (BAB18904.1) *Carassius auratus*

MEWNGRLLVQLLMLVCVLEVSLCQHWSYGWLPGGKRSVGEVEATFKMMDAGDAVLSIPADSPMEQLLPIHIVNEVDADGLPLKEQRFPKRRGRV

>Chanchita3 (ADV31311.2) *Cichlasoma dimerus*

MEASTRVAMQVLLLALVVQVTLSQHWSYGWLPGGKRSVGELEATIRMMGTGGVVSLPEEASAQTQERLRPYNIINDDSSHFDGKKRFPNN

>Zebrafish3 (NP_878307.2) *Danio rerio*

MEWKGRLLVQLLLLVCVLEVSLCQHWSYGWLPGGKRSVGEMEATFRMLDPGDTVLSIPADSPMEQLSPIHIVNEVDAEGLPLKGQRFPDRRGRV

>Medaka3 (NP_001098142.1) *Oryzias latipes*

MDVSSKVVVQVLLLALVVQVTLCQHWSYGWLPGGKRSVGELEATIRMMGTGRVVSLPEDASAQTQERLRQYNLINDGSTYFDRKKRFMSQ

>Seabream3 (Q9IA09.1) *Dicentrarchus labrax*

MEANSRVMVRVLLLALVVQVTLSQHWSYGWLPGGKRSVGELEATIRMMGTGEVVSLPEEASAQTQERLRPYNVINDDSSHFDRKKRSPNK

>Whitefish3 (AAP57220.1) *Coregonus clupeaformis* MDLSSRTVVQVVVLVLVAQVTLSQHWSYGWLPGGKRSVGELEATIRMMDTGGEVALPEETSAHVSERLRPYDVISKKWMPHK

>Salmon3a (XP_014009933.1) *Salmo salar*

MDLSNRTVVQVVVLALVAQVTLSQHWSYGWLPGGKRSVGELEASIKMMDTGGVVALPEETSAHVSERLRPYDVILKKWMPHK

>Salmon3b (XP_014062302.1) *Salmo salar*

MDLSSKTVVQVVMLALIAQVTFSQHWSYGWLPGGKRSVGELEATIRMMDTGGVMVLPEETGAHVPERLRPYDVMSKKRMPHK

>Anchovy3 (AF070219.1) *Engraulis japonicus* MEQGRLVLLLVLACACKECVCQHWSYGWLPGGKRSIGGELEATFRMMDAGDTLIPLTAEKLQPNDAIIDDIEENAVRRGRRPLRRELLD

>Arowana3 (BAB72182.1) *Scleropages jardinii*

MELTGKSVLHLVVLAHVAQIGFSQHWSYGWLPGGKRSTGDTEAKVKMMDSGDLVTFFEEASPFVPESLGTNQFGSEDGGEFTRKRKWMHQKRII

>Mackerel3 (ADP89593.1) *Scomber japonicus*

MEASSRVTVQVLLLALVVQVTLSQHWSYGWLPGGKRSVGELEATIRMMGTGGVVSLPEEASAQTQEGLRPYNVINDDSSHFDRKKRFPHK

>Tilapia3 (XP_013126782.1) *Oreochromis niloticus*

MEAGSRVIMQVLLLALVVQVTLSQHWSYGWLPGGKRSVGELEATIRMMGTGEVVSLPDEANAQTQERLRPYNIINDDSSHFDRKKKVP

>Pejerrey3 (AAU94308.1) *Odontesthes bonariensis* MEASSRVMVQVLLLALVVQVSLCQHWSYGWLPGGKRSVGELEATIRMMGTGGVVSLPEEASAQIQERFRPYSVINDDSSHLDTWRKKKVLE

>Catshark3 (MH468812) *Scyliorhinus canicula* MEVTKIVSVHFLIAIVFTAHGCISQHWSHGWLPGGKRNAVSMDAYLEMVNDEDIITDFEIPKYQYLYQKMNSPPAYIPDISDRKFQEKRKLQSNLQQNTD

>Whaleshark3 (ND) *Rhincodon typos* MEVTKTISIHFLIAVMFIAHGCISQHWSHGWLPGGKRSAVSMDAYLEMINDEDVITDFEIPRYQYLYQRANNPQAIIPDLNDRKIPKKRKLQSNL

>Lampreyl (AAF78456.1) *Petromyzon marinus* MALRGQSLTLLLLATALLVSLNYAQHYSLEWKPGGKRDLEVSHTRELEQELEPPSNAFECDGPECAFSRVPNTKLIRELASYLSQRNYDRKGALK

>Lampreylll (AAL12249.1) *Petromyzon marinus* MALRGQSLVLLLLASALLVSLTHTQHWSHDWKPGGKRDLEAMRPLLEEELEAPNSAFECDGPECAFARVPTGELVREIVSYLSQKNYQRKVLK

>Amphioxus (AHE40598.1) *Branchiostoma floridae* MAARLPALLAVLLLAQILCARAFTYTHTWGRKRADSSELLTPHAAADSVSAAEVYDASEGSEVTKEDFKMAVRTLFRILGDYLQKRTNQN
