# Supplementary material for: Characterization of Gonadotropin-Releasing Hormone (GnRH) Genes From Cartilaginous Fish: Evolutionary Perspectives
Source: Front Neurosci. 2018 Sep 6;12:607. doi: 10.3389/fnins.2018.00607 (PMC6135963; doi:10.3389/fnins.2018.00607)
Supplement: DATA SHEET S2 — Amino acid sequence alignment of the GnRH family used for the phylogenetic analysis. The alignment was made using MAFFT and then manually optimized. [file Data_Sheet_2.PDF]

Alignment: C:\Users\htostivi\Dropbox\Disque dur réserve\Article Gaillard GnRH\Phylogénie\mafft-
Seaview [blocks=10 fontsize=10 A4] on Wed Feb 21 11:04:16 2018

```
                 1
Human1           MCL------- RM-------- ---------- ---------- -----KPIQK LLAGLIL---
Panda1           MFL------- RM-------- ---------- ---------- -----EPIPK LIAGLLL---
Rat1             ---------- -M-------- ---------- ---------- -----ETIPK LMAAVVL---
Koala1           ---------- -M-------- ---------- ---------- -----ELTQK LVAGCLL---
Chicken1         ---------- -M-------- ---------- ---------- -----EKSRK ILVGVLL---
Pelican1         ---------- -M-------- ---------- ---------- -----EKSRK IFVSILL---
Duck1            ---------- -M-------- ---------- ---------- -----QKSRK AFVGILL---
Alligator1       MYTSAASKNC RM-------- ---------- ---------- -----QKTRK VFVSLLL---
Turtle1          ---------- -M-------- ---------- ---------- -----EKTRK LEVRELM---
Gecko1           ---------- -M-------- ---------- ---------- ---------G SLLTSFL---
Sturgeon1        ---------- -M-------- ---------- ---------- -----AVSRG AFVWLLLS--
Gar1             ---------- -M-------- ---------- ---------- -----KAQKS SLFWLVVA--
Eel1             ---------- ---------- ---------- ---------- -----MADKS ALLWLGLA--
Whitefish1       ---------- -M-------- ---------- ---------- -----EEKKV LLLLLLLV--
Medaka1          ---------- -M-------- ---------- ---------- ------VVKT WMPWLLV---
Seabream1        ---------- ---------- ---------- ---------- -----MAAQT FALRLLLVGT
Chanchita1       ---------- -MHRSF---- ---FLR---- ---------- -----MAAKI LALWLLLA--
Tilapia1         ---------- ---------- ---------- ---------- -----MAAKI LALWLLLA--
Mackerel1        ---------- -MHR------ -----R---- ---------- -----MAMQT LALWLLLL--
Pejerrey1        ---------- ---------- ---------- ---------- -----MAVRT WALWPLLV--
Xenopus1         ---------- -M-------- ---------- ---------- -----KAIST YALLLLV---
Bullfrog1        ---------- -M-------- ---------- ---------- -----SRHVT VVLLLAIV--
Catshark1        ---------- -M-------- ---------- ---------- ---------K LLVCFALG--
Medaka2          ---------- -M-------- ---------- ---------- --------SR LVLLLGVL--
Chanchita2       ---------- -M-------- ---------- ---------- ------CVSR LVLLLGLL--
Tilapia2         ---------- -M-------- ---------- ---------- ------CVSR LALLLGLL--
Seabream2        ---------- -M-------- ---------- ---------- ------CVSR LVLLFGLL--
Mackerel2        ---------- -M-------- ---------- ---------- ------CVSR LVLLLGLL--
Pejerrey2        ---------- -M-------- ---------- ---------- --------SR LVLLLVLL--
Whitefish2       ---------- -M-------- ---------- ---------- -----VSVAR LVFMLGLL--
Salmon2a         ---------- -M-------- ---------- ---------- -----VSVAR LVFMLGLL--
Salmon2b         ---------- -M-------- ---------- ---------- -----VSVAR LVLMLGLL--
Goldfish2        ---------- -M-------- ---------- ---------- -----VHICR LFVVMGML--
Zebrafish2       ---------- -M-------- ---------- ---------- -----VLVCR LLLVMGLM--
Eel2             ---------- -M-------- ---------- ---------- -----VNTGR LVLILGVL--
Anchovy2         ---------- -M-------- ---------- ---------- -----ACGYR WVLLAAVL--
Arowana2         ---------- -M-------- ---------- ---------- -----VCVGR LTLLLGIL--
Sturgeon2        ---------- -M-------- ---------- ---------- -----ACQGK LLVLLAVL--
Coelacanth2      ---------- -M-------- ---------- ---------- -----ACQRS LVILLEVL--
Bullfrog2        ---------- -M-------- ---------- ---------- -----ACQRH LLFLLLVL--
Gegko2           ---------- -M-------- ---------- ---------- -----ACHRP LLLFLCIM--
Turtle2          ---------- -M-------- ---------- ---------- -----ACQRP FLLLLLVV--
Alligator2       ---------- -M-------- ---------- ---------- -----ACPRS LLLLLVLL--
Elephantshark2   ---------- -M-------- ---------- ---------- -----ALQRN LLLLLLVL--
Catshark2        ---------- -M-------- ---------- ---------- -----AFQRN ALFLIFLL--
Whale            ---------- -M-------- ---------- ---------- -----AFQRN LHFLVFLL--
Xenopus2         ---------- -M-------- ---------- ---------- -----ACQGH LVLLLIVL--
Koala2           ---------- -M-------- ---------- ---------- -----SCLRP LLLLGLLV--
Lampreyl         ---------- -M-------- ---------- ---------- -----ALRGQ SLTLLLLA--
Lampreylll       ---------- -M-------- ---------- ---------- -----ALRGQ SLVLLLLA--
Chicken2         ---------- -M-------- ---------- ---------- -----APGGC LLLALLLL--
Human2           ---------- -M-------- ---------- ---------- -----ASSRR GLLLLLL---
Panda2           ---------- -M-------- ---------- ---------- -----ASCRL GFLUIL----
Sheep2           ---------- -M-------- ---------- ---------- -----ASFGL GLLLLLLL--
Elephantshark1a  ---------- -M-------- ---------- ---------- -----SALGK RLLWLSLT--
Elephantshark1b  ---------- -M-------- ---------- ---------- -----SVLGK RLLWLVLI--
Lampreyll        ---------- -MNERIDCCL SLALWR---- ---------- -----MGRAS LSLVLILW--
Anchovy1         ---------- -M-------- ---------- ---------- -------RSK GALVCLLL--
Goldfish3        ---------- -M-------- ---------- ---------- -----EWNGR LLVQLLM---
Zebrafish3       ---------- -M-------- ---------- ---------- -----EWKGR LLVQLLL---
Chanchita3       ---------- -M-------- ---------- ---------- -----EASTR VAMQVLL---
Mackerel3        ---------- -M-------- ---------- ---------- -----EASSR VTVQVLL---
Seabream3        ---------- -M-------- ---------- ---------- -----EANSR VMVRVLL---
Tilapia3         ---------- -M-------- ---------- ---------- -----EAGSR VIMQVLL---
Medaka3          ---------- -M-------- ---------- ---------- -----DVSSK VVVQVLL---
Pejerrey3        ---------- -M-------- ---------- ---------- -----EASSR VMVQVLL---
Whitefish3       ---------- -M-------- ---------- ---------- -----DLSSR TVVQVVV---
Salmon3a         ---------- -M-------- ---------- ---------- -----DLSNR TVVQVVV---
Salmon3b         ---------- -MTVPF---L TTLIKRTHDY SHHTVRNRRN GDTSMDLSSK TVVQVVM---
Arowana3         ---------- -M-------- ---------- ---------- -----ELTGK SVLHLVV---
Anchovy3         ---------- -M-------- ---------- ---------- -----E-QGR LVL-LLV---
Catshark3        ---------- -M-------- ---------- ---------- -----EVTKI VSVHFLIA--
Whaleshark3      ---------- -M-------- ---------- ---------- -----EVTKT ISIHFLIA--
Amphioxus        ---------- -M-------- ---------- ---------- -----AARLP ALLAVLLL--
```

```
                 61
Human1           --LTWCVEGC SSQHWSYGLR PGGKRDA--E NLIDSFQE-- ---------- ----------
Panda1           --LTLCVVGC SGQHWSYGLR PGGKRNA--E KLIDSFQE-- ---------- ----------
Rat1             --LTVCLEGC SSQHWSYGLR PGGKRNT--E HLVDSFQE-- ---------- ----------
Koala1           --LTVCVTIS SGQHWSYGLR PGGKRDA--D NLIDSFQE-- ---------- ----------
Chicken1         --ETASVAIC LAQHWSYGLQ PGGKRNA--E NLVESFQE-- ---------- ----------
Pelican1         --EVMSVEIC LAQHWSYGLQ PGGKRNA--E NLVESFQE-- ---------- ----------
Duck1            --FIVSVEIC LAQHWSYGLQ PGGKRNV--D NLGELFQE-- ---------- ----------
Alligator1       --LILSVDIC LAQHWSYGLQ PGGKRNA--E NVVESFQQ-- ---------- ----------
Turtle1          --FILSVEIC LAQHWSYGLQ PGGKRDA--E NLVESFQE-- ---------- ----------
Gecko1           --ULLCVAIG SAQHWSYGLQ PGGKRDA--E NLIESFQE-- ---------- ----------
Sturgeon1        --LMAVSEVC YGQHWSYGLR PGGKRET--E TLLDTLQE-- ---------- ----------
Gar1             --MTLVTQAC -SQHWSYGLR PGGKREV--E SLQDTLQD-- ---------- ----------
Eel1             --VALVCQGC -CQHWSYGLR PGGKRGA--D SLQDTLQD-- ---------- ----------
Whitefish1       --VALVSQGC -CQHWSYGMN PGGKRAT--G SLSDTQDN-- ---------- ----------
Medaka1          --SSVLSQGC -CQHWSFGLS PGGKREL--K YFPNTLENQ- ---------- ----------
Seabream1        LLGTLLGQGC -CQHWSYGLS PGGKREL--D GLSETLGNQ- ---------- ----------
Chanchita1       --GTAFPQGC -CQHWSYGLS PGGKRDL--D TESDALGN-- ---------- ----------
Tilapia1         --GTVFPQGC -CQHWSYGLS PGGKRDL--D NESDTLGN-- ---------- ----------
Mackerel1        --GSVVPQVC -CQHWSYGLS PGGKREL--D SLSDTMDD-- ---------- ----------
Pejerrey1        --GSVLLQVT -CQHWSFGLS PGGKRDL--D TFSDTLDN-- ---------- ----------
Xenopus1         --LLFSAHVG HAQHWSYGLR PGGKRDA--E SLQDMYPE-- ---------- ----------
Bullfrog1        --LLLSSHMI HGQHWSYGLR PGGKREV--E SLQESYAE-- ---------- ----------
Catshark1        --SAIFVNEL SAQHWSFDLR PGGKREA-DD DLVESFQE-- ---------- ----------
Medaka2          --LYVGAQLS QAQHWSHGWY PGGKREL--D SF---E---- ---------- ----------
Chanchita2       --LCVGAQLS FAQHWSHGWY PGGKREL--D SFGTSE---- ---------- ----------
Tilapia2         --LCVGAQLS FAQHWSHGWY PGGKREL--D SEGTSE---- ---------- ----------
Seabream2        --LCVGAQLS NAQHWSHGWY PGGKREL--D SEGTSE---- ---------- ----------
Mackerel2        --LCVGAQLS NAQHWSHGWY PGGKREL--D SFGTPE---- ---------- ----------
Pejerrey2        --LYVGAQLS YAQHWSHGWY PGGKREL--D SFSTSE---- ---------- ----------
Whitefish2       --LCLGAQLS SSQHWSHGWY PGGKREL--D SFTTSE---- ---------- ----------
Salmon2a         --LCLGAQLS SSQHWSHGWY PGGKREL--D SFTTSE---- ---------- ----------
Salmon2b         --LCLGAQLS SSQHWSHGWY PGGKREL--D SFTTSE---- ---------- ----------
Goldfish2        --MFLSVQFA SSQHWSHGWY PGGKREI--D VYDPSE---- ---------- ----------
Zebrafish2       --LCLSAQLS SAQHWSHGWY PGGKREI--D LYDTSE---- ---------- ----------
Eel2             --LCLGAQLS LCQHWSHGWY PGGKREL--D SLTTAE---- ---------- ----------
Anchovy2         --LFLGVELS GAQHWSHGWY PGGKRDV--D TENSAQ---- ---------- ----------
Arowana2         --LCSGAQLS CSQHWSHGWY PGGKREL--N SLTASE---- ---------- ----------
Sturgeon2        --LALSAQLS SGQHWSHGWY PGGKREL--E GLQSPE---- ---------- ----------
Coelacanth2      --LAVSIQLC STQHWSHGWY PGGKREL--A IPQTPE---- ---------- ----------
Bullfrog2        --FAVSTQLS HGQHWSHGWY PGGKREL--D MPASPE---- ---------- ----------
Gegko2           --IIATIHLS KAQHWSHGWY PGGKREV--D LSQSPE---- ---------- ----------
Turtle2          --LAVSTHLS RAQHWSHGWY PGGKREL--D LSQAPE---- ---------- ----------
Alligator2       --LAIGVPLA RAQHWSHGWY PGGKREL--D LSQAPQ---- ---------- ----------
Elephantshark2   --LAINTQVS RAQHWSHGWY PGGKREL--G QAQTPE---- ---------- ----------
Catshark2        --LIVNTQFS RAQHWSHGWY PGGKREL--S LSQSPE---- ---------- ----------
Whale            --LIVNTEFS TAQHWSHGWY PGGKREV--S LSQSPD---- ---------- ----------
Xenopus2         --FAFSTHLS NAQHWSHGWY PGGKRQL--D TRSIPE---- ---------- ----------
Koala2           ----LWTQIS YAQHWSHGWY PGGKRAL--D EIPGLE---- ---------- ----------
Lampreyl         --TALLVSLN YAQHYSLEWK PGGKRDL--E VSHTRE---- ---------- ----------
Lampreylll       --SALLVSLT HTQHWSHDWK PGGKRDL--E AMRPL----- ---------- ----------
Chicken2         ------AGTA QGQHWSHGWY PGGKRDL--- ---------- ---------- ----------
Human2           --LTAHLGPS EAQHWSHGWY PGGKRAL--S SAQDPQNALR -------PPA GSPVQTAHGL
Panda2           --LTVHPGSL KAQHWSHGWY PGGKRAS--S SAQHPQRAPR LLGRVLGTAA SSPDQAAHNL
Sheep2           -LLTTHPGPS KAQHWSHSXY PGGKRAS--S LPRDPQHPPR -------PPA QSPGQIAHTL
Elephantshark1a  --LAVLTALT SAQHWSIDNR PGKKRGT--E HMIEFLQG-- ---------- ----------
Elephantshark1b  --LAVLTALT SAQHWSIDNR PGRKRGT--E HMIEFLQG-- ---------- ----------
Lampreyll        --LLTAPPAS LGQHWSHGWF PGGKRGV--Q EPPRAS---- --YENVSPSD GSPFTP----
Anchovy1         --VTAAVLQC SSQHWSHGLS PGGKREA--D SPSESQ---- ---------- ----------
Goldfish3        --LVCVLEVS LCQHWSYGWL PGGKRSV-GE ---------- ---------- ----------
Zebrafish3       --LVCVLEVS LCQHWSYGWL PGGKRSV-GE ---------- ---------- ----------
Chanchita3       --LALVVQVT LSQHWSYGWL PGGKRSV-GE ---------- ---------- ----------
Mackerel3        --LALVVQVT LSQHWSYGWL PGGKRSV-GE ---------- ---------- ----------
Seabream3        --LALVVQVT LSQHWSYGWL PGGKRSV-GE ---------- ---------- ----------
Tilapia3         --LALVVQVT LSQHWSYGWL PGGKRSV-GE ---------- ---------- ----------
Medaka3          --LALVVQVT LCQHWSYGWL PGGKRSV-GE ---------- ---------- ----------
Pejerrey3        --LALVVQVS LCQHWSYGWL PGGKRSV-GE ---------- ---------- ----------
Whitefish3       --LVLVAQVT LSQHWSYGWL PGGKRSV-GE ---------- ---------- ----------
Salmon3a         --LALVAQVT LSQHWSYGWL PGGKRSV-GE ---------- ---------- ----------
Salmon3b         --LALIAQVT FSQHWSYGWL PGGKRSV-GE ---------- ---------- ----------
Arowana3         --LAHVAQIG FSQHWSYGWL PGGKRST-GD ---------- ---------- ----------
Anchovy3         --LACACKEC VCQHWSYGWL PGGKRSIGGE ---------- ---------- ----------
Catshark3        --IVFTAHGC ISQHWSHGWL PGGKRNA--- VS-------- ---------- ----------
Whaleshark3      --VMFIAHGC ISQHWSHGWL PGGKRSA--- VS-------- ---------- ----------
Amphioxus        ------AQIL CARAFTYTHT WGRKRAD-SS ELLTPHA--- ---------- ----------
```

```
                121
Human1          IVKEVGQLA- -----ETQRF E-----C-TT HQPRSPLRDL KGALESLIE- -EET-GQKKI
Panda1          IAKELDQGA- -----EPQHL E-----C-TI HQPQTPLRDL KGALESLIE- -EEN-GQKRI
Rat1            MGKEEDQMA- -----EPQNF E-----C-TV HWPRSPLRDL RGALERLIE- -EEA-GQKKM
Koala1          MADEGNQLA- -----EPQRF E-----C-TI HQPRSPLRDL KGVLASLIE- -GEA-GRKKA
Chicken1        IANEMESLG- -----EGQKA E-----C-PG SYQHPRLSDL KETMASLIE- -GEA-RRKEI
Pelican1        TANEMEKSG- -----EVQKT E-----C-PG LRQHSRFSDL KEAMESLIE- -GEA-RRKKI
Duck1           IANDMEKIG- -----EVQKT E-----C-PG SYQHPQFTDL KEAMASLIE- -GEA-RRKKI
Alligator1      MASDMEKFG- -----EMQQF E-----CSSG PHQPSKLSDL KKALASLIE- -GEA-GRKKT
Turtle1         IASEMEKIG- -----EMQHF E-----C-TG PHQRSMLSGL KGALASLIE- -GDA-GRKKI
Gecko1          IANEVDKVG- -----ELQHL E-----C-TA SQQRPTLQGL KGALASLID- -RET-GQKKI
Sturgeon1       IA-DIEKLD- -----TGDHS E-----C-AL SSQRSQLSDL KGVLARLVG- -GES-ARKKI
Gar1            IAEEVRKLD- -----AIRQP G-----C-AD VSPQSRLSSL RELLASLAE- -EER-GRKNI
Eel1            IIEELQKLD- -----TSSLP S-----C-ND LSPHITLSSL KEILANLAD- -RET-GRKNI
Whitefish1      MAEDLLKID- ----PSCSLF G-----C-AD VSPHAKMYRL RALLASLAD- -RQS-GLNNI
Medaka1         I--RLLNSN- ----TPCSDL S-----H-LE ESSLAKIYRI KGLLGSVTE- -AKN-GYRTY
Seabream1       IVGSFPHVA- ----TPCRVL G-----C-AE ESPFPKIYRM KGFLDAVTD- -REN-GNRTY
Chanchita1      MVEEFPRVE- ----APCSVE G-----C-AE ESPFAKMYRV KGLVGSVTE- -REN-GHRTY
Tilapia1        MVEEFPRVE- ----APCSVE G-----C-AE ESPFAKMYRV KGLLASLAEG KTDT-GHSRN
Mackerel1       VVEGFPQVD- ----TPCSFL G-----C-AE ESPFAKIYRM KGLFGSVTN- -REN-GHRNY
Pejerrey1       VVEGFPHMD- ----APCRVV G-----C-AD ESPFAKIYRM KGFLGGVTD- -REN-GRRVY
Xenopus1        TPNEVPLFP- -----ELERL E-----C--- SVPQSRLNVL RGALMSWLD- -GEN--RKKI
Bullfrog1       VPNEVS-FT- -----ELQHL E-----C--- SIPQNRISLV RDALMNWLE- -GEN-ARKKI
Catshark1       DAGNVDGLTH -NSRMGCPFP D-----C--- ---------L RGTLAKFTP- -----RRRKL
Medaka2         VSEEMKL--- ------CETG E-----C-S- YMRPQRRSFL RNIVLDALA- -REL-QKRK-
Chanchita2      ISEEIKL--- ------CEAG E-----C-S- YLRPQRRGIL RNILLDALA- -REL-QKRK-
Tilapia2        ISEEIKL--- ------CEAG E-----C-S- YLRPQRRSIL RNILLDALA- -REL-QKRK-
Seabream2       ISEEIKL--- ------CEAG E-----C-S- YLRPQRRSVL RNIILDALA- -REL-QKRK-
Mackerel2       ISEEIKL--- ------CEAG E-----C-S- YLRPQRRSFL RNIILDALA- -REL-QKRK-
Pejerrey2       ISEENKL--- ------CEAG E-----C-S- YLRPQRQNVL RNIVLDALA- -REL-QKRK-
Whitefish2      ISEEIKL--- ------CEAG E-----C-S- YLRPQRRNIL KNILLDALA- -REF-EKRK-
Salmon2a        ISEEIKL--- ------CEAG E-----C-S- YLRPQRRNIL KNIILDVLA- -REF-QKRK-
Salmon2b        ISEEIKL--- ------CEAG E-----C-S- YLRPQRRNIL RNILLDALA- -REF-EKIK-
Goldfish2       VSEEIKL--- ------CNAG K-----C-S- FLIPQGRNIL KTILLDALT- -RDF-QKRK-
Zebrafish2      VSEEVKL--- ------CEAG K-----C-S- YLRPQGRNIL KTILLDALI- -RDF-QKRK-
Eel2            VLDEIKL--- ------CDGG E-----C-S- YLRPQRKSLL KNILLDALA- -REFQRKRK-
Anchovy2        VSEEIKL--- ------CEAG E-----C-S- YLRPQRRNLL KSILLEALT- -REF-QRRK-
Arowana2        VSGKIKL--- ------CEDR K-----C-S- YLRPQQKNIL -TILVDAST- -REFRGKRK-
Sturgeon2       DSDEVKL--- ------CDGD E-----C-S- YLRHPRKNIL RSILADMLT- -RQM-QRKK-
Coelacanth2     VSEEIKL--- ------CDGE E-----C-T- YLRSPRKSIL KEILADIIA- -WQI-QKKK-
Bullfrog2       VSEEIKL--- ------CEGE E-----C-A- YLRNPRKNLL KNILADVLA- -RQL-QKK--
Gegko2          VSEDIKL--- ------CDGD D-----C-T- YLKIPREKIV TSLLADLLA- -KHL-QKKK-
Turtle2         ASEEIKL--- ------CDGE A-----C-A- YLRSPRKTIV NTLLADLLA- -RQL-QKKK-
Alligator2      ASEEIKI--- ------CGGE E-----C-A- YVRSPRMNVV KTLLADMLA- -RQL-QKKK-
Elephantshark2  VSEVFQL--- ------CEGD D-----C-A- FVRSPRTNLF RSILADLVA- -GRF-QKKK-
Catshark2       VSEEIKL--- ------CRGD G-----C-L- FLGSPRKDVI RSIVTDMLM- -QQI-QKKK-
Whale           ASEEIKL--- ------CQGE G-----C-L- LLRSPRRGII RSIVMDMLV- -QQI-QKKK-
Xenopus2        ISDELKP--- ------CEGE S-----C-D- YPMNE-MSIL KGLLTRFLF- -PRE-RQRK-
Koala2          ASEEGKL--- ------WDGG E--------- ------RSLL KTLLADVLA- -QQQ-QK---
Lampreyl        LEQELEPPSN ---AFECDGP E-----C-A- FSRVPNTKLI RELASYLSQ- -RNY-DRKGA
Lampreylll      LEEELEAPNS ---AFECDGP E-----C-A- FARVPTGELV REIVSYLSQ- -KNY-QRK-V
Chicken2        SAPQVPAALR -----PCPTP P-----C--- RPLPPMPSTL RAALWRPLE- -AAL-RQPH-
Human2          PSDALAPLDD ---SMPWEGR T-----T-AQ WSLHRKRHLA RTLLTAARE- -PRP-APPSS
Panda2          PSNALAPPEN ---SVPWEAR T-----T-GW WPLRQKQHLV KTLLTGRRA- -SRP-VALQ-
Sheep2          PSDALAWPED ---SVPWKSR T-----M-TR WFLRGKQHLV QTLLVSKVEG -PHP-WPLQG
Elephantshark1a VAGEVEELIQ SRGRATVELP E-----C--- SGDNPGKMVL RKNI------ ----------
Elephantshark1b VTGEVEELLQ SRGRATVELP E-----C--- PGDKPRKMVL RKNM------ ----------
Lampreyll       VSSGLQVADW ---HVVCSRS PNGFSGC-AM CCSPGCPTFL SQVLEASLG- -T--------
Anchovy1        VMEGLPRRG- ---GARCGSD T-------RD AANQERPSTL EQLISLMSRA -NEV------
Goldfish3       VEATFKMMDA --GDAVLSIP AD-------- -SPMEQLLPI HIVNEVDADG -LPL-KEQRF
Zebrafish3      MEATFRMLDP --GDTVLSIP AD-------- -SPMEQLSPI HIVNEVDAEG -LPL-KGQRF
Chanchita3      LEATIRMMGT ---GGVVSLP EE------AS AQTQERLRPY NIINDDSSH- -FD--GKKRF
Mackerel3       LEATIRMMGT ---GGVVSLP EE------AS AQTQEGLRPY NVINDDSSH- -FD--RKKRF
Seabream3       LEATIRMMGT ---GEVVSLP EE------AS AQTQERLRPY NVINDDSSH- -FD--RKKRS
Tilapia3        LEATIRMMGT ---GEVVSLP DE------AN AQTQERLRPY NIINDDSSH- -FD--RKKKV
Medaka3         LEATIRMMGT ---GRVVSLP ED------AS AQTQERLRQY NLINDGSTY- -FD--RKKRF
Pejerrey3       LEATIRMMGT ---GGVVSLP EE------AS AQIQERFRPY SVINDDSSH- -LDTWRKKKV
Whitefish3      LEATIRMMDT ---GGEVALP EE------TS AHVSERLRPY DVI------- -----SKKWM
Salmon3a        LEASIKMMDT ---GGVVALP EE------TS AHVSERLRPY DVI------- -----LKKWM
Salmon3b        LEATIRMMDT ---GGVMVLP EE------TG AHVPERLRPY DVM------- -----SKKRM
Arowana3        TEAKVKMMDS ---GDLVTFF EE------AS PFVPESLGTN QFGSEDGGE- -FT--RKRKW
Anchovy3        LEATFRMMDA --GDTLIPL- ---------- --TAEKLQPN DAIIDDIEE- -NAV-RRGRR
Catshark3       MDAYLEMVND ---EDIITDF E-----IPKY QYLYQKMNSP PAYIPDISD- -RKFQEKRKL
Whaleshark3     MDAYLEMIND ---EDVITDF E-----IPRY QYLYQRANNP QAIIPDLND- -RKIPKKRKL
Amphioxus       AADSVSAAEV ---YDASEGS E-----V-TK EDFKMAVRTL FRILGDYLQ- -KRT-NQN--
```

```
                  181
Human1             ---------
Panda1             ---------
Rat1               ---------
Koala1             ---------
Chicken1           ---------
Pelican1           ---------
Duck1              ---------
Alligator1         ---------
Turtle1            ---------
Gecko1             ---------
Sturgeon1          ---------
Gar1               ---------
Eel1               ---------
Whitefish1         ---------
Medaka1            K--------
Seabream1          KK-------
Chanchita1         KK-------
Tilapia1           ERFL-----
Mackerel1          KK-------
Pejerrey1          KK-------
Xenopus1           ---------
Bullfrog1          ---------
Catshark1          ---------
Medaka2            ---------
Chanchita2         ---------
Tilapia2           ---------
Seabream2          ---------
Mackerel2          ---------
Pejerrey2          ---------
Whitefish2         ---------
Salmon2a           ---------
Salmon2b           ---------
Goldfish2          ---------
Zebrafish2         ---------
Eel2               ---------
Anchovy2           ---------
Arowana2           ---------
Sturgeon2          ---------
Coelacanth2        ---------
Bullfrog2          ---------
Gegko2             ---------
Turtle2            ---------
Alligator2         ---------
Elephantshark2     ---------
Catshark2          ---------
Whale              ---------
Xenopus2           ---------
Koala2             ---------
Lampreyl           LK-------
Lampreylll         LK-------
Chicken2           ---------
Human2             ---NKV---
Panda2             ---------
Sheep2             QLRTEVVGG
Elephantshark1a    ---------
Elephantshark1b    ---------
Lampreyll          ---------
Anchovy1           -------YD
Goldfish3          PKRRGRV--
Zebrafish3         PDRRGRV--
Chanchita3         PNN------
Mackerel3          PHK------
Seabream3          PNK------
Tilapia3           P--------
Medaka3            MSQ------
Pejerrey3          LE-------
Whitefish3         PHK------
Salmon3a           PHK------
Salmon3b           PHK------
Arowana3           MHQKRII--
Anchovy3           PLRRELLD-
Catshark3          QSNLQQNTD
Whaleshark3        QSNL-----
Amphioxus          ---------
```
