# Supplementary material for: Characterization of Gonadotropin-Releasing Hormone (GnRH) Genes From Cartilaginous Fish: Evolutionary Perspectives
Source: Front Neurosci. 2018 Sep 6;12:607. doi: 10.3389/fnins.2018.00607 (PMC6135963; doi:10.3389/fnins.2018.00607)
Supplement: DATA SHEET S4 — Sequence of catshark GnRH1 (A), GnRH2 (B), and GnRH3 (C) genes, inferred from the cDNA sequences given in Figure 1. Exons are indicated by colored boxed. The exon/intron boundary consensus sequences (GT/AG) are highlighted in red. Initiation (ATG) and stop codons are boxed. Polyadenylation signals are underlined. The identity of the scaffolds containing the sequences are indicated. [file Data_Sheet_4.DOCX]

A. Catshark GnRH1 gene

**sc 691705**

#### …tatagggtggatccgtgggtttgaggagggtgatcatggctcggcacaacatcgagggccgaagggact

#### gttctgtgctgtactgttctatgttctatgtgcagggtaggtggattggccgcgctaaatcgccccctta

#### attggatagcagaagaatttcttttaaaatcaattctttttttaaaacaatattcattcttagagctgat

#### ctatatggcacgtacaccccaccccacttatttttccaactgacaaataaatcaatctatgttttaaaat

#### ttgcagctcacgacgccgtcgctctcccggaatg…

**sc 669879**

tcacccacgatgaaattgctggtctgttttgcgctgggcagcgccatctttgtcaacttcctgtctgcac

**M K L L V C F A L G S A I F V N F L S A**

agcattggtccttcgatctgcggcctggtgggaaacgagaagctgatgatgaccttgttgaatctttcca

**Q H W S F D L R P G G K R** E A D D D L V E S F Q

ggaggtcggtccctcccccctgtgttttccccaattccctttcggaatccacacggtggcacagtggtta

E

gcactgttgcttcacagcgccagggtcccgggctcgattcccagcactgcctgtgcggcgtccgcccgtt

ctctcccccgtgtctgcgtggggtttcctccgggcgctccggtttcctcccacaagtcccggaagacgtg

cttgctgggtgaattggacattctgaattctccctccgtgtacccgaacaggcgccggagtgtgacctcg

ccggagtttagaagagagtggatcttgctgaaacgtataaaatccttttcgattttaacggcgtcaatga

gcttggggagaatcgggaacatgccttcgcgatcaaggattagccgtaatgagcagtctcgaagggctga

atcgccttctgctattttctatcgttctatgtttaatcttgttgaagttaacacctggaggaagggacgg

aagggtcagggtcatccttatgagattgtatcccgtgttaactggagattaatggtgtttaatagtgccg

aggcgtcgctcaatctataaacaggaggagatagaaacgggagtgtgttgggaggtccttaagacatagg

agcagaattaggccattcggcccatcgagtctgctccgccattcaatcatggctgatatctcatccccat

tctcctgccttctccccgtaacccctgacccccttattaatcaagaacctatccatctctgtcttaaaga

cactcagt

**sc 433394**

ggggtaggtcccgtgccaaccggtgctgaagagcctccgccggccggcacgagttggcacacgcgcggga

gtgccagcgtggtgctggtgtcatcccagcgcatgcgcggtggggggggtncgggagtgccagcgtggtg

ctggtgtcatcccagcgcatgcgcggtggggggggggttcgtctccgcagcggacggtgcggagggaaag

ggtgcccccacggcacagggccgcccacggatcggtgggccccgatcgcgggccaggccaccgtgggggc

agcccccgggggccagatcccccccccccccgaggaccccggaggccacccgcagagctgggtccccccc

gttaagtaccaggtctaatttacgctggcggaaccagccagaaacgggcggccgctcggcccattgcggg

ccggagaatcgccgaggggggggtcggagaatcatgcccagtgttttgcaatgaatgtgtccctgtgggg

gaaagacgagcggtctaaccttattgtccttcattccaggatgccgggaatgtggatgggttgacccaca

D A G N V D G L T H

acagccggatggaatgtcccttccctgattgtttgcgagggactttggtaagcacgggatttgacaatgg

N S R M E C P F P D C L R G T L

gctttgatctttatccctttcccggtacgatttccttggcctgaattccgcgtttctacaaacctctcct

tcaaagtgccactgagtgttaacctggccttgtcgaccacagtgacctgcacactcaaaaaccctctcgc

aatcggttacgctatctggccgatagttacctgttttttttcgtctccctcagttttactttggcagttt

tccaatcctctggtactttccagaattgaagaattcttgtacgattactaccagtacatttctgtcgcta

cttccttgaacatcctggaattcaacccatcaggtccaggggacttcctggccattagcctactttccct

aatacattttctctcgtgtgagttgatgtatttatttcctccccactctcttctctatctcttaattatt

tagtatttttggaatgatgttactgtctcccattgtgaagactgacacaaagtgtttgattaactcctct

gtcatctcctggttccccattgccatttcccagttttgttctcgaagaacgtgcagactccacacagtga

cccaagccgggaatcaaacctgggaccctggagctgtgaaggaactgtgctaaccactgtgctaccgcgc

ttccctatatatatatgccttgctgtccgtttttatatcaatagctggtttgccctcaatttaggggctg

gtttagctcactcggctaaatcgctggctttgaaagcagaccaaagcaggccagcagcacggttcgattc

ccgt

**sc 243066**

ggctaaatcgctggctttgaaagcagaccaaagcaggccagcagcacggttcgattcccgtaccagcctt

cccggacaggcgccggaatgtggcgactaggggcttttcacagtgacttcattgaagcctactcgtgaca

ataagcgattttcatttttaattttttcttttatcttctccctttttagtataatttttggtcatccctt

gttggtttttaaaaaacttccccaatcctctggtttaccactgatctttgccacgttatatgcattttct

ttcagggtaataccacccttcacatccttggttaaccacggctgacttatccccttcccagaatccttct

tcctcaccgggatatatctttgtcgtgagtcatgaactattttcggtgactcatcaacaagaccctttcg

ggcatcaacaattccctagcctctcccaccaaaacaagaagcgagtggggccgtttaaatgaaccatgtg

tcaaaattgttcgattcctgtcttttccaggcaaagttcacaccgaggaggaggaagctt**tag**cggggaa

A K F T P R R R K L -

tggcgatcaatattccggggtagtggaacaataaatcgcttctccttttctcccgccggcagttgtgaag

acggccggtcctgaccagatggtttctgggaaccgtatgttactgaagcgtcgagtgg**aaataa**aatgaa

gtgtttgaactcgttgagtcacagtgtggtcttattcgctctccgttcactcaatatgtctctttggaaa

gaggcctggagcggggattctccgtcccgttttccggcacggttttcccattatggccaccatcgggaaa

ctccgtgggtgtagatgcgctgccgaggaaacggaggagcccgctgttggcgtttgatgagatgctaggc

cgagagcctaaccacgtcggacagcatcagtctgccttctgtgcagtaaaatgtcccaaggtgctttgca

gaagcactatccgtgtcttacattgcagcacgtcccgttcccctattataaccccccctccccacacccc

ccccggggcgcactctggctccttgcctggccacaattttcaaatgatcaccctcgtcctcaaaccccct

ccatggtctcatctctctccatatctcctccagtgtaaggctccttcctgtttattcccatcattcccct

ttcttttattctgccgtccttgttttgcttcatggacacgcccctttaagacaagcggcctgtaccttta

attcctgcaggagcgatcactctgccagcctgtgctggtttgagcaggagaccacagaggtgccagcatc

agtgagcaagatggggtgagactcggtttgattggtggccaatgaattggcccaaaaggccgcactctgc

ccggtaacaggtggggattggatcctgtccaaatgggatgcttttcagcatcctaaggaatttcagtttt

gattctggcagagcagagggagaaccgactatctatcgctccagaaagcctatgagtgctaactctctcc

agaaagcctgtgagtgctaactctctccagaaagcctgtgagtgctaactctctccagaaagcctgtgtg

agagctgcatttctaaaactacagagaacctggatgaatgaatctacagagaccattctgggctgcaaac

aaagacttacccataagcgtgctgtgaaagaagatatgcaaagaaaaacatcatctgaaacataaactct

tttccctttcatttatggtttttacccctctccgcccctcggtgtttgtctgtctcttgtgtgtgtgtgt

gtgtcgagggtgggcaggcaagttaaaatggggaattaggaatgagttaatagttaaccagttgtactcg

gcgtatcggtcattatagttcctgctataaacaaacgctaattgtgttaaaacttacaaaccaggtgact

gtaattatcgggcagcaacgtgccaaagacttcagacgttatgatgagaaatattggtcaattcactggt

gttgcgactccaggacctgtcgggattgaattgaccacacactagcccaggatgtcgtaaccccagaccc

ctccgcgatctctccgctgcccaaaaccgggcctcttgagcactcccccgattattttctttaaaattta

gagcgcccaattattttttttctccaattaagggggcagtttagcatggccaatccacctaccctgcacc

tctttgggttgtgggggtgagacccacacaggcaacggggagaacgtgcaaaccccacacggacggtgac

ccggggctgggattcgaatccgggtcctcagcgccgcagtcccaccgctaaccactgcgccacatgccgc

ccccccccaccgaatataatcgctccgccattgtttgcccgtgacccccaagatctggaactccgctctc

ccctgaaatgggacgaactcgattggaaccttggcagtgagaggagggcggggaggcgttttgtcttacg

agttgagtccgcgcgcgtattcgcatccaacaccttttggaatcaatttgtcctgaagtcggattaatgg

ctctatttcttgcatggaggctggatgcgaacctgataccaaaaaaaccttctgttcaatggttgtctct

ccaaagactaccgg

B. Catshark GnRH2 gene

**sc 291231**

…ttcaccccgatacggtaaggcgctgctccctcgcggtacaccccgctaaccagagaatccccctagcct

ccagatcccactccgtggcgatccgtggggtattgcatcgccaccctcaccgtccagcacggaccgattc

tccgaccgggctttgggggggggggtcagagaatcccaccccttatcatcagggatgtgcaataaatgct

ccccttgccaggaatgcccatatccataatggaagatttacaaaatctcacacagtttacttaggatgat

ctgaacaaacgcatataaggatgtaaatgataaatatgacggtcttccacatctgcaattgtgaagttca

gagaccagcctataatgcattttagcaactggtgatttttcgaaagggatatcaaatcttggtaatgtaa

agaaggatgaagtgttgttaaggttgaccgcggtgcacattttagccattggtgaacaacgattgccaat

atttagcctaagttgtttctaacctcccctggcagctacagtgtttactctgatgtgcaaatagaaagat

tcaatctccagggactgcagcaacctacaatcccagattggaagcgggctgagttgaacactgtttgcgc

atggtcataatgtattgatcactttctgtaggcaggatgattccaaattgaagctgctacgttcatcgca

gagccaaggacggtatatatagaggggtagagagccgagtgagtcccgatcagtctactgaaagcgaaca

agaaataacctcgagcttctgacaggtgagccccaactactccaagtctttgttcgatttggatatcatt

ttgcaagttttcttatgaataacaagaagatcataacataagctaacagaaagttgccgatttaattagc

acataggattttcaggcttgtaatctatcagtaatttaataggaaaaagttgttccacttgaaaagtgtg

tttcgtttcaaactggcactgttgactcactttcttttccttttattgctgtgagctgagtccttttaca

tttaagatcttggctgtccctcactgcaggttgaatattacttgtggttaagattgttgctaattgttca

gagcttttgatgtctctctccaccactaatgcttccttaaactacatttgcaaaatgcagggtgaagaat

tccagccattagatttaaactggcgtgattttgaaggattaaccaaacagaattaaatgttgaagttaaa

gttttactgacagctggtggtcatcctcgtggtgtcagcagataccagggggaatattttgtgtgctggg

gcttgttgcacggaaaccggctcactaacatagaaaatagagcaggaggaggccattcagcccttcaagc

ctgctctaccattcattatgatcatggctgatcatccaactcaatagtctaaccctgcctccccccccat

atcctttgctccccttcgccctaagtgctatatctaattgcaagagacacgtctcgaacagtggagcact

cactcagcagacagcctcaaactaggttgtgtgccaaaatctttccaacagtacttgaacccacagctga

ctgacacagtgatgggatatcacagttgacactaattgtgtaatgtagaacaccagcaaggttagaaaag

tgtgttacgataacacagtttgcaaggacttttttttgccgctctagctctagttaaaaacattgttaaa

caaaaaatatttcatgggatgtggatgggtgtcgggagctaggcccagatttattgcccagccctaattg

ctcttgagaaggtggtggtgagctgctttcttgcaccacagactggcttgctaaaccacagttaaggatc

aatcacattcctggggatctggagtctcatgtcggccagaccagataattaggcagatttccttccctat

gggacagtagcaaaccagatgagtttttaatga

**sc 224214**

atattgtcggggcccatagccactgcagtatccagtgctttcagctgtttcttgatatcacgtggagtga

atcgaattgactgaaaactggcccctgcgatgctgcggacgtctggaggagcccgagatggatcatccac

tcggcactcgtggctgaagattgttgtgaatgtttcagccttgtcttttgcactgatgtgctgcgctccc

ccattattgaagatggggatatttgtggagtgtgtttatgcacaaatcctagaggaagaagggacaggct

aacaatgtggtttataatgc

ttggattcttgtctttataaatataggcatagacgtggaagttatgcgtaagttttataaaacactagtt

ctgccccagctacacccaatccagttctgggtaccacattccagaaaggaggtgaaggctccttttggag

agggtacagaagacatttactcgaatgattccaggaatgaggggttataggtacatggatagattggaaa

atcttgagttattttccttggtgcaaaggaggctaggaggagattagatagaggtgttctttaatgttct

ggttgagcaaataagtgaattctttccttgtttgaagggtcaagaaccaaagagaaacaggtgacatgat

agtaaactttttttatgcaaccaatgtttaggatttggaatgtgctttctgatcgggtggtggatatagt

tttgaaaactagccttcagcagggaattggatattaaacacttgaaggaggaaatattgcagaggcatgg

tgaagtcagcagggtggaactaacaggatttctccatgtaagagccagcactgactggatgggccaaatg

gcttgtttctgtgctgtattattcaatgatcttacttgaattatcagtgctgtcagaaatattttccata

acttgtcaaccataatggctgtctcgtttttcaccagttcggtaaatctgacacagcaattacatctgtg

gctacatc**atg**gctttccagagaaacgcgctcttcctgatctttctgctactgattgttaatacccagtt

**M A F Q R N A L F L I F L L L I V N T Q F**

ttccagagcccaacattggtctcatggttggtatccaggaggaaagagggaactgagcctgtcccagtct

**S R A Q H W S H G W Y P G G K R** E L S L S Q S

cccgaggtgagtcctactgtatttgcatttctgaaactttgtacaatctcaactccttcctaaaatggca

P E

ggtgactggaaattctgaatctcataagcagaataatcaccctatatgtgtttggtctccgcaatgtggg

agattcaagctggtgtagtgcaagaagaatgagagggaatctcatagaaacatacaaaatcctggatagg

ctagatgtgggaagaatgttcccgacattggggacgcccagaactagggatcacaacctaagaatatggg

ggaagccattcaggactgagatgaggaagaacgttctctctcagagttgtgaatttgtggaattctttac

cacagaaagctgttggggccagttcttggatatattcaagtgggagttggacatggcgcttgaggctaaa

gggatcaaggagtatggagagaaagtgggagtgagatactgaatttgcatgatcagccgtggtcatattg

aatggtggtgcaggctcgaagggccgaatggcctactcctgcacctattttctgtgtttatatgtttcta

agtcatacttagctgcagaaagaatatttctcttttataatataggtttcaaacgttgaaatggaaagat

aaaaatcggtttcacctagccatagtgccaggtagaatttatttttgtaattaattctcaggcaatggat

taagagtaaaatagtaggacaatttgttaatttgttcaaggagccacacatgcagatgaacatgcaaaca

ttggagccactctgcccttccagcctgctgcacaattctggctaatctgattgtaaccacaaccccacat

tcctgcctacccccaataatctttcacccccttgcttatcaagaatctacctctgcctcaaaaatatttc

aagaagagagttccaaagactcacaaccttctgagagttagaatttttcctctttgtctgaaatgagcga

cggtgacttctagttctaggttctcccacaaggggaaacatcctttccacccctcaagatcttcactgtt

tcaatcaagtcacctcttactcttctaacttccagttgatacaagcccagcctgttcaatcctttctcat

aaaacatcctgcccattagtcaagtaattagtcaagaattagtctggaattagtcaagtaaaccttccct

gaactgcttctaaaccattgacatccttccttaaataaggagatcaatactgtagaccatagtccagatg

tggcctcactagtgctctgtaaaactgaagcattgctcactactttcatattgaaatttcctcacaataa

atgatatcattctattagccttcctgagaattgctgtagccgtgcactaacctagatccctctgcatctc

tgagctctccaatctctcaccatttattcttcctgccaaaatggacaacttcgcactttttcacattata

ccctatttgtcatatctttgctcaatcatttaactatatatatatatatacatatacatgtatatctctt

tgtagcctccttacgtcctcttcacagcttactttcctacctatctttgtgacgtcagcaaatttagcaa

ccctacctttcttcatccaagtcatttatataagttctggccttagatgatggtctgtgtgaagtttgca

tgttttccccatgtttgcgtgggtttcctctgggtgctccggtttcctcccacagtccgaagatgtgcag

gtttggtggattggccatgataaattgccctt

**sc 227869**

tggagtttgcacattctccccgtgtttgcatgggtttcgccccccacaacccaaagatgtccagggtagg

tggattggccacgttaaattgccccttaattggaaaaaatgaatcaggcactctaaatttattaaaaaca

agggaaggtgctatgcctagttcttgaactgtataatctggtgtcaaacatgatgccacaatgttgttaa

tgtacggacttccaagattttgaccaaataacgatgacaaaacgctgatgtatgtccaaggcagcctggc

atatgtttcggtgggaaaaaggatatggcatcccccatgagtctggtactcttatccttctggtacccca

agccaatggaggtcatgaatttgggatgtgatgctgatgttgtttgactgtatatggtgggggagatggt

gacctagtggtaatgtcactggacccgcaatccaaaagctctgggcgtactgctctggggacacgggttc

aaatcccaccatgacaactggtagaatgtaagttcaattaattcatttggaatatagagctagtctcagt

aatggtgagcatgaaaccatcatcaattgttgtgaaaaagcttgatcactaatgtcctttagggaaggaa

atcttccatctttacctggtctggcctacatgtgactccagacccacagcaatgtggtggactcttaaca

gatcttgtatgtggcccagaaagccactcagttccagggcaacaagtgttgaccttgccactgatgccca

tattaattcattaatttattcatttacgggatgtgggtgtgatcccatgaaagaataaaaaaaacacaaa

gaatcatagaatttacagcacagaaggaggccattcggcccatcgagtctgcaccggcccctggaaagag

cactccacttaagctcacacctccaccctatccccgtaacccagcagccccacctaaccttttttaggac

actaagggcaatttagcacgtccaatccacctaacctgcacatctttggactgtgggaggaaaccgaagc

acctggagcaaaccgatgtagacacggggagaacgtgcagactccgcacagacagtgacccagccgggaa

tcgaacctggaaccctggagctgtgacgcaactgtgctaaccactgtgctaccgtgcggcccgaggagca

ggagataactacttatttttaaaagtgcgtaggggggttgaggggagaactaaactaagggtgagaagtg

attggaaaagtccaattgaatgtttcttcttattccccaggtttcagaagaaatcaagttatgtcgagga

V S E E I K L C R G

gacggttgcttatccctgggaagtccccgtaaagatgttataaggagcattgtggtaattttaaaattct

D G C L S L G S P R K D V I R S I V

tactggttgaggaaataaacatagtgacttgcgaaagcaaactcatttctaaaatgtgtaaaggtttcta

actgcgctttgctcataagagaaggattaatgaagaaacccggattcaccgtgagtttgtactgcacaaa

cccacaagcttcacagggttgtgttgagaaagggctgcagttgggtggaaagtccgaagaaattgggatt

gttttcagtacagcggagaaggctaaagagagatttatcgctcatgtgtaaaatgggagggttttggagt

aaatacaggaaaaatgattccagtggtggtctcagagcaggtttaaggtaattggcaaaagaatcataga

agataaggacaagagtggcctcaagtgagagtgctaaattgggggaaagctaacgacaacagaattcggc

aagagcagaataatgtgaattgggagcagctgtttgaggataaatccacaatttttttgtgtaaaacgtt

ttattaaggtatttataattttataataacagtaaacagtacaaatacagttataaacatagtgcataac

ccatcttattgcnttgcctctgctgacagttaattttccccgaagaagtcgacaaacggctgccacctcc

agacgaacccgagcattgaccctcttaaggcgaacttaattttctcaagtctgagaaacccggccatgtc

actaacccagatctctgatttcgggagcttcgagtccctccacactagcaatatccgtctccgggctacc

aaggaggcaaaggccaaaacgccagcctctctcaacccgtggactcccggatctgccgacactccaaaaa

tcgccacctccagactcggcaccacccttgtttttagcaccgtggacatggcctcggcaaatccctgcca

gcatcccctcagcttcggacatgcccagaacatatggacatggtttgctggccctcccgcatacctcaca

cacctgtcctctagtccaaaatacctgctcatccagcccactgtcatgtgtgcccggtgagccaccttga

attgtatcaggctgagcctggcacatgaagaggacgtattgactcgctcaaagcatctggccacagaccc

acctctatctcctcccccccccagctcatcttcccatttgcactttagctcctctatctgagttacctcc

gactccatgagttctttatagatatccgagactttcccctctcccaacccccgttctagaaactaccctg

ccctgtatcccctgcggcggtaggagcgggaaggtcgaaacctgccttcggacaaaatctcttatcttca

gatatcaaaacccattccctcccggcaattcgaacttctcctccaaatcctataaacaggaatgctccca

tcaataaatagatctcccatcctctcaatccctgctctctgccatctccgaaaccccccatccagcctcc

c

**sc 24595**

aatggtatacttgttttgaaggggaacggccacgagggatccctgcactgtctgtctgttccctttcctt

cccctgactgtaacccagctactcttgtcctgcactttgggtgtagctacctccctttaactcctctcta

tcaccccctctgccttccggatgatccgaagttcatccagctccagttccttaacgcgatctccgaggag

ctggagttgggtgcacttcccacaggtgtagtcagtaggaacactatgaaggttattaattatctttaca

ttggaaataagtaaagcatatctttagatctggataaatgtgaaatgatcgtaactttgttttttctccc

catttgcctccctgtctacttcttaacatcagactgatatgttgatgcaacagattcagaagaagaaa**tg**

T D M L M Q Q I Q K K K -

**a**agtccaccttcttttctgattgtccctgatgtaaattgtaacccctgaagctcgtatgagtgaaaacat

ttttctccaaaatatgattttaaagtctgcaacaagtctgtgtcaaatattcgaaggttcctgctcccat

ggcttgaggactatcaacagataccgtggggcagtttgtgtggtataaattcaaacacactacagaccgc

attctctttctgacatcttagttcgcatcttgtga**aataaa**ggactttattttgctacatatcaacacca

gctctcaaatgtcattgtttg…

C. Catshark GnRH3 gene

**sc 39961**

…tgtttactaagagagttaatttcaatgaaggtgtgaaacattgcttttagctgtatgctaaaaattcact

tggttataacttgaaaggtttacatttatcacctaaatcaagtgaaaccctcatccatgtttttccagatg

cttactaagatagttactttcaatgaaggtgtgaaccattgtttttaacttcatacaaaaatcctgtgtgt

ttgcttgggagaaggcatcttcattttataatcctgctctttgcagctgctgttaaccctttgtgggatct

tcccctgtattctctcatgtttctctcagaccagatattgccagacttccatgtttcaaatgacacatctt

tccattacaaggtcagattgacaaaaatgttagatgcctatgtttggacccctttggacaatattgttgtg

ctgaaatagaggccaaagttgccaaaagagaataatacctttgactgtgatgatgtacagattgaataaaa

ttcctgcaactgcccagtcagatggttaagtgaatttgtaagctttaaagctcagttaagtgcttaaaagt

agagtgcattttaatataccaagggatttttctttcaaaagaacatgttttcatcttaatgtccttaatgg

attatatacttgttataggtttagttatggaaaaaaaaaaatccctttgttgccaattagggatacaatgg

tgatctgctatatattgaagatccgcaactgaaccttttacttttcgataaaaagaataaaggacccttga

gtgcagagtgctcttgccaagtaattacctactggtcacatctagcactattagtctgttaacactaaata

aggaataggtttgttttgtatattttcctgagagctaatttctatttctcattccattttatagacgaact

tctatttttcctggagccttttcctcttcattcca**atg**gaagttaccaagatagtttccgtccatttcctg

**M E V T K I V S V H F L**

atagcaatagtttttactgctcatggctgtatctctcagcactggtctcatggttggctgcctggaggaaa

**I A I V F T A H G C I S Q H W S H G W L P G G K**

aaggaatgccgtaagtatggatgcatatctagaggtaagactcagtctttattccattgttatttgttatg

**R** N A V S M D A Y L E

agcaagtgacttcagtcaaggctgcaacactccttgtctaataataatcatctttactgtcacaagcaggc

ttacattaacactgcaatgaagttactgtgaaaagccccaagtcgccacattccgacgcctgttagggtac

atgaagggagaatttagaatgtccaaattacctaacagcccgtcttttgggattgtgggaggaaacctgga

cagacacagggagaacgtgcagactccgcacagtgaccaaagccgggaatcgaaccttggagcccggcact

gtgaagcaacagtgttaaccactgtgccgcctcatatattgatgttaatgactggtgccaggaaatgcatc

atttgcacaaacctttcttaaaacttttccccaacttacctcaccgctgctggtgctaactataaggaagc

taaggttctgtgaagattcaaactatcccttgccaagacgccagcagaagaccggtgggaaccccagagaa

attatagatttgattatgttcagtcatttccctgttttttctgggattcagctgctcctcttcggccatga

gagtttccatggaatttcagccctagaatttttaccttcaccagagctgagctcagctaactcagtacaga

ggagggttgagtcagcaacttctcaggtttgtactctttaacatcttttatgatataatttgttccctatt

gaagtgagctactttagctgtttgcatccatttccaacaacaagctcacatattaaatggctgccaaacat

tccaacttgatggctcagcttatatttcaaatctgttgacaggatgagggtagtgagaagtgaacgagtga

cttcaaccaaccaggtataagcaggaaatcttcaagcttgcagagaatattcagtaaagaataattcattt

acattgcacatgctttcttttgcataatgctagcgattaacttctacttggcttggtccaagcaagcaatc

agcacagcatttaacatgtagaagtagtgaatgttttatgaattagtggttataacaggattatgtttggg

cagcacggtagcacagtggttagcacagttgtttcacagctccagggtcccaggttcgattcccggcttgg

gtcactgtctgtgcggaatctgcacgttcttcccgtgtctgcgtgggtttcctctgggtaccccggttccc

tcccacagtccaaagatgtatgggttaggtggattgaccataataaatagtccttagtgtccaaaaaggtg

aggttactggtttacggggatagggtggaggcatgggcttaagggggggtgctctttccaagagccggtgc

agactcaatggaccgaatggcctcgttctgcactataaattctatgatatttgataaacaggaaacggggg

tggggagggagtggatggggagtgtcggctgacaggtcaggtggagtagggaacaggggagcagaagaggg

gtaagtgttgagggagcctgacccttcacatatgtgctctggtactgactgacagcagaatagctcaaaca

cttagaagacactattaagatcttttctccacaattttataatttagtccaaattatgagccagcagtgtt

gactggggttaggttttgtatatcgttgaggagaattgatgtccgcattggtgtgagagtaaatatttatg

aagtgctttgctgctgacctcatgcattgttggctgaccatgtgagatagtttttcaggctgttagccaac

gaaggttttaaaaatcacgctgagcattgctgcagcatccagagtatcatctcacaaggaactaaaatcca

ttgtgataagtaaagtgaaagggaaaaacatatttttagaagcaaactgaggaaaagatggaagtgtttga

taaaagtgggggaggtataaataaaatacctgcgcaataaacaaatgctataaatttacattggtgatgga

acaaaaaaaggaaaatcttgttgaggcctggatttttctgtggtaatggtgaaaaatctgtcagcgttcac

tgcaataaaacagattgaaagttcaggagcttagccatgtgcaggataatgtggaaatctaaaagctgcta

tgactaagttcaggcttggttcatgaactgacaagcactttcactctaacgctgttagtctcactgtaaaa

acctggaaaagaattgcaccttgttaaaataagatgtttttgatgacataccaatgtcataattaaacact

ttcactggccctgaaaataattttatatttggaagattgtcaaattttcccataattaattaaaagttcca

catgcattcaatgtgaaatattcataaaatcaataaaaagataaagattattttcttaaaaagtctctttc

tcttcattttcattcccacctttatattgtcacacacttacttcacaatttttaaatttaaattaaaagtg

aaggattttaagtgctttcaattctttggtttgctgtgtataaacacttcagtttgattgattgcctacct

gcaactggacacaggtcatccccttgttttgacagcaggtttaaactggcgttggaaagggagcatttcca

ccaaagtgatcactaaaccttgttggacagcttttatcgtggtcagcagcaagtgtccttgccgaccgcaa

aatcagggataatataagtcattctaaatcagagatttaatttttttaataaatatttttattttccattt

tcacactttcttcagaatttacaccccaccaacaaatagtaaatggtaacgaatacaatgtcaatcccttt

gataacaacaacaatcccatcctcccaccatcccccaaacaacggcccacctgacaatataagcatcaaat

aaaacaaaccctcccaaggtggaaaaaaaggaaaaaagaaaaaggaatcaggaatcgcctatggtcaccat

tgacatatacnnnnnnnnnnnnnnnnnnnnnnnnnnnnnnnnnnnnnnnnnnnnnacagtaagaagtctta

caacaccaggttaaagtccaacaggtttgtttcaaacacgagctttcggagcacagctgaagaaggagccg

tgctccgaaagctcgtgtttgaaacaaacctgttggactttaacctggtgttgtaagacttcttacaaata

tatacagcattggctcatttagtacatacaccaacacgcagtgaaaaaagaaagttaatgaggctacatca

tcaattctgccttcgcaaactcctccgctgtttcagctgttctaaaataatagtccttggatttgtaggtc

accctcaacttagctggatatcctctgtcgcactgtaccttgctgatgtacagtgccttcttcacccggct

gaaggcagcccgcctcctggccagctccaccgcaaagtcctggtatatacctatacctgctccagcccacc

gcaccactcgctactgttttgcccagcacatgactttctccttcactttatacctacggaaacacagagtt

actactcttggcagctcttacgcctttggtataggcctccacgatcaatgagcccgatccagttcatatca

ggagggatcgtccccctcactcaatagctttgcatcgtggcaaaatactccgtcgatcttgggctttccaa

ccctttgggcagacccacaatcctcagattctgtcgcctggatctgttttccaggtcttccattttggctc

acatacccttgttggtttctatcacgctccgcaactccttccccaacgaggtgagttgatcattgtgctgc

aataaagcctcttccactgccttcagtgtctcaccttgctcctgcactccgccgcagcgcttgataccgcc

gccctcaccggggcaatcgcctcctccaccagcactttcaataccgtctccatctccttcttcatcgcttc

catgtgttttgtgaactgttttttcaagttccaccaccttcaccttggtcatttcttctgccgtgagcgat

gcggcctcccctggtgcctcagcctccgctttccttgcagttcctgcgctgacttttccactcaccggcgg

actttcattagccccctttttcacggccatttttctcccaaacttggacatttctcctccctgtgccttct

tacggcctttccagcctccgttgcccccgggaccgggcattaaaactccgaaattcctattcccgagcggg

agccctccagtgtgcagctgcctcctgcccgccgtcaccagaagtgccctaaatcagagagttgaggagaa

aagaacaaactaggttaattatgtattaaaatatttcttaaatttgaagaaaacaatcggagtaagaatga

atgctgctgctgaggattagcagacgaactggatgatttcaataaaaaaagtcatgagtggcaattgaata

aactagctaaatccagctccaaatcttttgctgcatcctgcatattgctctcattatttaatttctaggtg

attagtatagattagaaaaaatattatgcaagcaggatgccattcaacatcttcaccagtctgacattatg

aatctcacaattaatctctgtttgctaattcaggacaactttactgagtcccatgttttctacctgaatcc

tctgtattcaagtttaattataagtctttaatgtcaaaacctttttaaagggtataaatgaaggatataat

gtggattaattgtgaaaaagagtacagaggttaatgatagtaattgaagaaacactaattgttgcactgac

agcatgtaagttttccatatcaataccagtgaaagacccatcacaactatatttgcacattcatttgcagg

cacagctgtcaggatttgcccccctacactctcagctttccatacatttgtcaaactcttatagtgatatc

ccaggtaattaaaaaccatgatcacactcacaccgccactaaacctttacccctcatcccacccctctgtg

gtgaatcagaatatgaagttttgctggtgggggaatgattaataattaatcaattaataatacggtggtgg

ggggggaggggttagcaaatgagaggagagtgtgttcaggcacatggcaatcctgcctagctaaatgccct

ttcctcctccatcactaggtcggccataatatttcacccttatagtttttcaagcttcacaactaaacggg

cattattgctccaaatcacagttgaacgaccaggtctcttttttttttaagtgaagagtttagtcagcatt

aaattacaaactttatgggaatacccttaacagaattgtgcatgtgtttatcatgctatgaatgctgtgta

tgtactgacctcagtgcggaaggaaaattgcatgttgaaaacattagttgcaaattaatttagtttgcatt

gagcagattgtggttccttgattgattgattttgctcactggttcgtctcattcagtctagtagctttccc

tacctggaacgtaaccagtggatatttgagccaatcaacatggctaattaaacaggttatttaaaccaatt

caagtggcacagcataaatggttaaagtgtgatggatgttgcaccccatgtgtgataccctgtgatgcatg

atatacacaagactatcaattaaatagagaagcaaaagaagaatgcgtttagagagcaccttttcatatca

caggaacatgttgagccacttagcatataattaattttttaaaatgtagttccgagtacttagataattaa

gatagccattttgtgcaccaagaacgatgggattaataactattaatctggtaggaatacagataagcata

ttttgaaaaataatgcacacacaatacccagtttgtaaatctggggtaagtttgaaatgaagaatctgttc

tctgaacccagaatagttatgcccttgcgaataaatgtatcatatatatgtatgttttatccctcaacggg

gagatggtggtttagtgataatgtcactaatccagaacctcaagctgtattttccctgcccttctctcctg

aaagcaagaaaaccttgatgggttcagttccacagactctgatcatcctccgcaattcacctgtagagatg

cattttctaactggaggactttaaacaattcaacctctcaaaattataatttggtaatttgtaaaaataat

ctttttcatttcagatggtaaatgatgaagacatcataactgattttgagatacccaaatatcagtattta

M V N D E D I I T D F E I P K Y Q Y L

taccagaagatgaatagtcctccagcctatatagtaagtatcattcataaaaagcacgatgatcctgaaag

Y Q K M N S P P A Y I

aaacataggcaatttggggaaatttgaattctttcagaaatgggcatgtttgggtcaggtggagagctgta

gtcgtaaaagtttgaatcccaaatccaaaccactcactccaggttttaaggggagtgtgatgaattgtggg

aataaccttaacaggttattctcaggctgtcaacccatccccaagagaagactgggcacattaaatattat

aatgatgttgcatgtctcatttttacacctcatttgaaattgaactctggttggccaagattcccaggcct

cgcgaaacccaacaactaaggtttaaaccaaggcgttaatttccacttggctcccttcaatcaggcctcag

acttaaaatcttgtccggaggtctccccatcctctgatctccaagctctccgcgatgggaatgaaacactc

gccaacccaacaccagattcccacacacctgcaggctgtaaaactatgccccaatgggaataaaaattggg

aaaaaggaacaccggttcgaattggtattctattttaaggcatacaagactagtttggaacagtactcttt

atttcccagcttttactgttttgtttcccattggacctgtgaactgcattttgccgaactagaaaccaagg

ctgtcaatcagattggcttctgggcagggaatctgttggtgatgtcttctgggttttctaatcgctactga

gcaaccacaacccaccgctcatgataattcacctcttggtgttgccactgtaggaattattttcttaacaa

tgtcaacgaataaaggtaatttggaatgaaagatgtggcactgtttttgttgtgccatttatattttcttc

acattgattgaattgtatttattaattattgtcccaattttggttgtgttccagccagacatcagcgatag

P D I S D R

gaaatttcaggaaaagaggaagctccaatcaaacttgcagcaaaatactgac**tga**caatttatcacaatta

K F Q E K R K L Q S N L Q Q N T D -

cctatttatttaaaaatatactttgttgcggagaagatttgttcaatattttgacattaagatttaagtga

aatgtattggtacatcggaccaactacatctgatactttgaagtagctaagcatgtatcactattagaagg

aatacagataaatattagattgtttccattgcctgaaagggtgtttccttttcatttttgataagtggata

catctttaatgttttctacagacaaacattcttgtaaggatcatcaagcctttgtccatgtatttaagaac

aaggcatattgccatgtgaattaaccatctaacattctaccacaagaacaaatgcttctaataattaactt

cttataacatactttgctcagaaatgccctggcaatcaaatgaaagtaagggcttgataaacttccagtca

tggtccatcaattgctcatgttgtacagcaaactacgttaaatcattagggttaactcaagagaagccatt

tcactgaaaactgaatttaaatccctccaaaataacaacataattatataataaaattgtcagatgtgaat

gtagaacatgccatctacatggtaactattaacctgacaattcagtcacctgtattgggttctcaatcaat

tttcttcttcctcctttctaattggctattttctatttttagtcaccaatcatccaaagcctttctcccca

tcattgtcaacggagcaaaaaaaactctgaagtacagtcagtcagtatccctaaaaataacatgtaaccat

ttatttaaaataaacggacccagttggctttaaggtcttttcttatttctgtgaagctatagaatcataga

aattacaatgccgaaggaggccattcggcccatcgagtctgcaccagcctctagaaagtgccccccaccca

aacccacacctccaccccatccccgtaacccagcaacctcacagaacctttttggacacagagggcaattt

agcatggccaatccacctaaccttaatttgcaattcggacatgcaaatgggccagcactctgctggca
