# Supplementary material for: Characterization of Gonadotropin-Releasing Hormone (GnRH) Genes From Cartilaginous Fish: Evolutionary Perspectives
Source: Front Neurosci. 2018 Sep 6;12:607. doi: 10.3389/fnins.2018.00607 (PMC6135963; doi:10.3389/fnins.2018.00607)
Supplement: DATA SHEET S5 — Predicted sequence of whale shark GnRH1 (A), GnRH2 (B), and GnRH3 (C) genes, inferred from GnRH coding sequence given in Supplementary Data Sheet S6. Legends are the same as in the Supplementary Data Sheet S4. [file Data_Sheet_5.DOCX]

Haut du formulaire

A. Whale shark GnRH1 gene

**NW_018048068.1**

…AATAGGCTGGGGACAGTTTTCCCTGGAGCGTCAGAGGCTGCGGGGTGACCTTATAGAGATTTATAAAAT

GAGGGGCATGGGGTGGGGGTGAAGAGATAAGGTCTTTTCCCCCAGGGTAGGGGAGTCCAAATCTAGAGGG

CATAGGTTTAAGGTGAGAGAGGGGAAAGATTTAAGAGGGATCTGAGGGGCAACTTTTTCATGCAGAGGGT

GGTGCGTGTAGGGAACGAGCTGCCAGAGGAAGTGGTGGAGGCTGGTACAATGACAACATTTAAAAGGCAT

CTGGATGGGGACATGGATAGGAAGGGTTTAGAGGGATAGGGGCCAAATGCTGGCAAATGGGACTAGGTAT

CTGGTCAGCACTGGACCGAAGGGTCTGTTTCTGTGCTGCATGACTCTAAGTAACAAAAAGGGGCAGACTC

AAGAAAAGTTGGCTTTTATTGCAAAGGAAATGGAGTGTGAAACTGGAGAAGTCTTGTTATAAGTTTGGAG

GGCTTTGGTGAGGCTACTGTACAGTTCTAGCTCTCCCTACTTGCATTAGCCCATTAGATATCGGAGCAGC

ATTTGGACATTTGGTCCATCGCCATTCAATCATGGCTGATCTGTTTCCCAACCCCATTCTCCTGCCTTCT

CCCTGTATCCCTTGATCCCCTTATCTCCGTCTTAAAGACTCTCATTCTCACGTAGCTGAATCCTGGAGCG

AGGGGTGTCCGTCTTCTGAGGGAGGGCTGGGTCTGCATCCGTTGGAGTTTGGAAGAAGGAGAGGGGATCA

TACACGAGGCAGATCAGATCCCGAGGTTGGGGGGCGCGGGGGGACCACGGCAAGGTGCATGCTCGGAGGA

TGTATCGAAGGATCATGGAGCACCACTCTCAAAATCAGGAATCCCCAGTCTAAGGTGGGCGTGAGGAGGA

ATTTCTTCCCTCGGAGCAGTAGCTCAGAATTCTTGCCCCCACGTGAGAGTGGGGGAGGCTGGTTTGAGGT

CAGCCTATTTAAGGCAGCCCCAGCAATTATCGGGTGTTCTAACTCCTCCTTGTTGCTTCTATGAGAGCCC

AGGAAACACTATGGGGAACGGACAGAGAGCTGGGGACATCCTGTAGAGGTTTGATTGCCTGATGATCATA

GGTTAACTGTGGTTGGAAGCTCTTCCAAATGGGAATTGACTGAAACAGGTGTTTTTGTTTTGGAGTTAGG

AGACATAAGCTTGTAATATGCGTCTCTGTAAGTAAATGCTCCATTGCAAATGCTCTATAAAAATATCGAA

TCCAGTTCCATCCCTTTCTGGATAGTATTTACAGGATGCAGTCATCACTCAGGAAGGATGGAACTCTTCC

TGTGTAGCTGAAAACTTGAAAGGATTGCAAAATCAAGCAACATGCACATTGTCATAAAATCTCAGCCAGG

TTGTAGATCGATGGAGAGTATGGAAGCTTAGTGCCGAAGTATCAATCCCCACTGTCAGTTGTCGGAATAA

GCCCATTTATTTCCAAGTCGGGGACGGTAAGGGGCTCGGAGGGTGAACTTGCAGGGGGTGGTGTTCCCAT

GTACCTGCTGCCCCTTGTCCTTCTGGATGGAAGTGGTCATGGGTCTGGAAGGTGCTGTCTAAAGATCTTC

AGTAAATTGCATCTTTTAGACAGTACACACTGCTGTTACTGAGCGTCGGTGGTGGGGGGAGTGGGATGTT

TGTGGATGTGGGGCCAATCGAGCAGGGGGCTGCTTTGTCCCTGGATGGTGTCGAGCTTCTCGAGTGTTGT

TGGAGCTGCCCCCATCCGGGGCAAGTGGGGACTATTCCATCACATTCCTGACTTGTGTCCCTTCGAATTC

TGAACATTTAAATGCGTACATTCTTCCGGAGTTTGCAATCTAGTATTTCAATGAGATTTCACTCGGTTCA

CGGCAATAATAGAGTCTTGCAATCTTCCCAATTTTACTTTTGGATTCATGACCGGAGCTGCATTTGCCTG

TCTTTTGAAAAAAGTGAGAAGACGGTGTTTCTTTTTAAAAAATTGGCAGGTCCGCTTGCTTCCTCACAAT

GTCATCTGCA**ATG**AAGTCACTTGTTTACCTCCTGCTGGCCTCTGCCATCTTGGCGAATCTATCATTGGCG

**M K S L V Y L L L A S A I L A N L S L A**

CAACACTGGTCCTTCGATCTGCGTCCTGGTGGGAAGCGAGCGGCCGGTGACACTGTCGTTGGAGCCTTCC

**Q H W S F D L R P G G K R** A A G D T V V G A F

AGGATGTAGGTATCTCGATTTCACCTTCCTGTAAGGTAGTTGGAGGCCATTCAGCCCTTCCGAGTCCTAT

Q D TTCTGCCATTCAGTGGACGGCCTGACCATCCCCCAATTTCACACCCCTCCTCGCCTTTCCTAGCCCTCCA

TTCCCCTTCCTGATTACAATCCAGCTCGGCCTTGTGTAGGCTTTACAATTCCACTGACTTAGCCCCCTCC

TCGGAGACAGGAAATTCCTCCTCATCTCTCTGTCTTCTTTATTCTGAGATCACATCCCAGACTCTCCCAG

GAGGGAGAGGGCCAAGCTATGGAAACAGGAGCAGGAGTGGGCCATACGGCCCATCGAATCTGCTCTGCCG

TTCAATACGATCTGTCTGTACCCTAATCCTGCTCTCCCCCATATCCCATCGATCCCTTTAGCCACAAGAG

CTATATCCACCGCCTTCCCGATAACAACAAATCCCGACTTCTCCGATCTCCACACCCGCCATCATCTAAC

ATCCTCCCCATCTGCAAGACGTGGGCTGGAAATTTCCCGAGCGTTTTCTTGCGTCGGAATGCTTCGCGAG

GCTTCCTCCCAACCTCTCAGAGGGTCAGTGGACGTCACACCGCGGTCTCCTAATGCTGGTGTTCGTGTTG

TTTCAGGCTCCCCAGGACGCTATCAACAAAAGGACTGAGATTTTCTCCCTGGACTGCCCCGGCTGTCTCC

CGGTAAATCCAATTCCTGACAAACGCCACTTGGGGTCAGGATTGCTCCGTGCATCGGTCCGAGTACATCG

ATCCAAACTTCGGGAGTGTTTTCCTAGCTAACTTTCTGCATTCCCCCCCTAGCGTTTCTATAGCCTTCCC

AAGTGACACTATCTCTTCAGTCCTCTGGGTTGATGACAGATAATCGAGAACTTGGACCTTTCCAATCGTT

GATTCGTTGCAAATGTACCGTCTTGGCATCCAATAACACCCTTTAAAAGCTCAGCCCAGTGATCCCCACC

CATGGCCTCGGCTGGTGGTGTTGTGGGCCCGCTAGCTCCTGAGAGATGCTTGTTGCAAGTTCCAATCATG

CTCCTGTTGACGTGACTGGTTTAAAATTTTCGGAATTAAAGATTAGGCTGGTCAAAATAGACCTGATTAC

CCCATGTCCTTCAGGGAAGGAAACCTGCCAATCTGGCGCAGTAGGGAAAGATTTTGATCCCCTTTAGATT

AGCCTGAGTTCTGGCTTCCACCTCAAGGCTACGAATCCGTCTTTGATCCCGATTTTCTGCCTCCGCTGTT

AAAGGTTTTGATTGTTGTTCGCTTCCTGGGGTAGCACCTACTTTTCTGTAAGGGCGGAAATATAGGAGCA

GGAGAAGGCCATTCAGCCCTCATAGCCTTCTCCATCATTCGATGGGCCGAACAGTCCCCTGTTTCCTTCT

TTCTCCCTGAGAACGACGTCAAACTCCTCCTTGAAAACATTTTCGAGGTTTAGCCCTCGCCCAGAATTCC

CACAGGCTCCCCACTCCCCGGGTGAAGACCTTTCCCTCTCATTTCTGTCCTACCCCATACCTGTAGACCA

TGACCTCCTGGTTCTGGGGATGGGGGGGTGGTGGTTGCGGTTGTGGGTTGGGGGTGTGTTCGGATGTGGC

TTTGCTGTGTGCTGACCCCTCTGATGCCCTCGTTTATCCCTCCAGGCAAAACGTTCACCAAGGAAGAAGA

AACTGTGATGTGGAACCACAAGACGACTTTAGCCCTCGAAGGGCAACAGCCTGGACTTTCAGCTCTTGTT

GAGAAAACCAGCATTTCTTATGGTGTTACTGATCAAAGGAAATAAAAGATTTTAACTCTTGATGAGTCTC

TGTGTCTTTTCCCTCTTGTTAATTCACACAAATGCTCCCCCTTGGGTAAAGAGTTCTTTACAGCACTCAC

AGGAATGTAGAAACCAGGGCAAGCGTAGACCACAGCTGATCCTCAATCTCCCGCTCTCTCTTTCCCCACA

CCCCTCAATACTTTTACCCTTCAGAAATCAATCTCTTTCCTGAATATATTCAGCAACTTGGCCTTCACAG

CCTTTGGGTCATAGAGTCATGCAGCATGGAAACAGACCCTTACCTTGTCGGGACTCCACAGCAACATGGT

TGTCTCGTGACTGCCCCCCTCTGGCCAATTAGGGATGGGCAATAAATGCTGTCCTATCCAGCGATGTCCT

TATCCCATGAATGAATAAAGGAAAATGGTCACCCCTCAGCTTCTGACGCTCCAGGGAAAATAGTCCCAGC

CTACTCAGCCTCTCCCTGTAGCTCAAACCCTCCAACACCAGCAAAATCTTTGTACATCTTATCTGAACCC

TTTCAAATCTCACAACATCCTCCCTATAGCAGGGAGGCCAGAACTGCACACGGTATTCCAAAAATGGCCC

TAACCAATGTCCTGTACAGCCWCAACATGACCCTCCCAACTCCTATACTCAATGCACTGACCAATAAAGG

CAAGYGTACCAAACACCTTCTTCACCGCCCTGTCTACCTGCAACTCCACTTTCAAGGAACTATGAACCTG

CATCCTGTTGGGCACAGGCGGGAAGTTAGAATTTGTCAGTAAAAATGATCGTGGGGGAAAGAGTGCTGTG

GTCCCTTTAAAAGCTTTGCTTTGCCTGTGCTTGGTAGTTTATAAATGCAGGTGCACACAGGCAGTGCCCG

AATGAAAACATTTTGTGTAGAACAGCTAAGCAGCAATTTTTTCCAAGACAACAAGTTTGAATTTGGCCAG

TTAATTTAAACCAAGCACCTAGAAACATAAAGCCAATTGAGTTTAAATCTGATGGCGTTAACAACCTGAA

ACCAATCACGTTATAAGAGTTTGATTATGTCGTTACGGGTATATAAGACGAGGCCATTTGGAAAATTGGG

GAGAGCCATCAGCCATCTGCTAGAGTTAGCACCATTCAGCTCTCAGAGAAAGCCCATCACATTCATCAAA

GGTCCCACCATATTTTAGCTCAAATTCCTCATTGAAGAAATTATAGAGGTAAAAAAATCCTTGACAGCAG

AATTAATGGAAGAAAGGTGTTTGTGAAGAGACTGGAGATGTCAAAATATTCCAGAAGATAGTCAACTGCG

AACTTGGAGAGATTAAGTTACAATTTATTGCTTCTTATTAATTGGGTTTATATTAGTGGGACCTGTCTTT

AATTAAACAGCGTTCCAGTAGAATTTAGACCAGTTATGGAGTAGTTAGTAGTCTAAGGGGGGAATTGTTG

GTTTGTTAGGAATTTTGTTAATTTGTTCATTGTTGGGTTAAATAAATAAATGGTTTCTTGTTATGTGTAA

AGTGGAGATCAGGGATTTTCTTTCTTTTAACCACACTTTTAACTAGAAATTATTTGTGGGGGAAAGACGG

ACTGCTCTGTGCTGTTTAGGGAACATTGTTAGAGAGGAACCCCTACTTCCTGTACATAGCAGGGATTGCG

GTATGAGTTTTACGCTTTAACTTATCACCCCCCCCCCCCCCCCCCTCCCCCACCCCCCCCCCCCCCCCCC

CCCCCCCCCCCCCACCACCTCCCCCCCCCCCCCCCCCCCCCCCCCCCCCCCCCCCCCCCCTCCCCCCCCC

GCCTGCAGGATTCAAAGCCATTGCCCTCTCCAGTAAAATTATTAAAGGTCTTTTTGTTCGGCAACACCCC

CCAGGCCCTTACCTTTAAGTGTATAAGCCCTCGCCTCGATGTTCCTGTTCCAAAAATTCCGCCCCTCACA

TTTATCTAAATTAAACTCCATCTGCCACTCCTCGGCCCATTGGCCCATCTGATCGAGGTCCCGGTGTACT

CTGAGGTAACCTTCTTCACTGTCCACTACAACCTCCAATTTCAGTGTCATTTGCAAACTCACTAACCGCA

CCTCCTATGTTCACATCCAAATCATTCATATAAATGATGAAAAGCAGTGGGCCCAGCACCGATCCCTGTG

TCGAGTCTGAAAGACAACCCTCCACCATCATCCTTTGCCTTGTACCTTTGAGCCCATTTTGGGAGCCTTC

TGTAGGAGGGAATTCCACAGGTTGACTATAACCTCCTGAGTGAAGAAATGTTTCCTCGTCTCAGTCCTAC

CTCGTATCTGGAGACTGGGTGCCCCCCCCAATCTACTGTTCTAGACTTCCCTCAACCAGGCGAAATAAAT

CTATCAAGCCCCTATTGGCATTCTTTATGTTTCAATCAGATTCCCCCTCTCGTTCTTCTGAACTTCAGTG

AATAGAGGCCCAATCTCACCTCATGGGACAGCCCTGCTATCCTTGGTATCAGCCTCGTGAACCTCCACTG

CACTCCCTCTATATCTTCTCTCACAGTAGGCCTGGAGCCTGGACTCTTGGTGATGACGGTGCCCAAAGTG

AGGACTCCTGGCGGCATTAGGCCCGGAGCCGGACTCCTGGCGGCATTAGGCCTGGAGCCTGGACTCCTGG

TGGTGGTAGGCCTGAAGCCTGGACTCATGGCAGCAATAGGCCCGAAGCCTGGACTCTTAGCAGTGTTGGA

CAGCAGAAGGGCCAGGACCTCCTGGGGCATCGACGCCGGCATTGTTGTTCCTGGAGCGGAGATCCTTGAG

GCTCAGGAGCACCTTTGGGTCCTTGCAGAAGCCCTGAAGCCATGCTCGAGACCCCAATTTGAAGAGAAGA

TTTATGTAATTTTTATTTTATCCTTATTGTAAGTAAAAATGACGCCGGATTGTGACGACAAAACACTTTT

CACTGTGTCTCCAGGATATATGTGACAATAAATAAATCATGCGTTCAATACTCCAGGAGTGGCCCCACTG

CTCATCTATATTCATCGACTTGCTTCTGAATTTTTCCACCACAGAAGCGTTTATCAAATTGACACAAGTC

ATCACAACTCTGTTACAGAATCCTATAATACGTCCCTTCGTCCCAACGAGTCTGTCCTGTCAAAAATACA

CCGAGTTGTGTGATCATCCCATTTTCTAGGCCCAAAGCCTGACACTTCAGGCCTGTGGTGCTTTTGTAAG

ACAGAGGCGAATGGTTTGGCTGAAGGGGTGGATTAAGCACCTTCGAGGGAGGTAGGCAGCCGAAGGCACG

GCCTCCAATGGTGGAGCGATGGGAATCGGGGGCTTAGAGACATCAGAGAGTTTTTGGGTCTCCTGGATAT

AGGACAGAGAGTGAAAGGCTGTGGAGGGATATGGTACCCATGGGATGCGAATGTTAAAAGTGAGGCCTTG

CCAGGGTGCAGGCCAGTGTAGGTCATTGCTTTTGTGTCTTTGATTGACAAACCCAATTTGTAAATTCTCA

TTAAAAAAAACCTACGCATTCCCAATCTCAGACAGCAACCCTGTTGCTATGGTATCTTTTGTCATCTGAG

CATTTAATTAAATTTCCCTTGCCATTGTAAAGAAACACTTAAATCATTTATTGTAAACCCATCTGCTTTT

TTTCTGAGCTAACTAAGGTCAAAAATTAAGCTTTGGAGGATCAAAGGATGGTTCTCAATCACAAAAAGCT

GATTAAATTTCCTCACAAAGTCGTCTTTAAATGGGTTTCTTAACACCTTTTTTTTTAATTCTCTAGGTCT

CAAAAGCCTGGGCCTTGAGTAGCACTCGCTCCCCAGGACTCCCATCTGTCAGGTGGGCTGAAACGGTGCG

GCCTGTTCTTCCAGGTCTCGCGGATTTACCTCGGGATCCTTTGCATTTTAAGTGAGAGTTTGGAGGAAAA

TGGAGGTTTTTCTTAGTCACAGTGGGTAGCATCAAAAATGTCACCCAGCCAGGTGGGAGATGCTGCCTAG

GATTAAACACTAGGCCCCCACTTCCACAACTCTCTGAAATTGAGCCAATCGTGGAATGGGGAATTGCGAT

GGGGCTTTGCGTAAACATTCTGCAGCATCTGATAGTGCCGAATGAGACCATTTGGCTCATTGTTGTAGGG

ACGGGTCTCGCTCAAATCCATTTGTCCGTGCCGCCCCATTTTCACAATTGATCGAGTCCCATTTGCCTGC

ATTTGGTCCATATCCCTTCATACCCAACCCATCCGTGTACCTGTCCAAATGTTCATCAAATGACAAAATT

GTACTCACCACTACCTCTGGCAACCCGTTCCAGACACTCACCACCCTCTGTGTGAAAATATTATCCCTCT

GGACCCTTTTGTATCTCTTCCCTCTCACTTATGCCCTCTAGTTTTACACTCCCCTACCCTGGGGAAAAGC

TGCTGGCTATCCATGCCTCTCATGATTTTATAGACCTCTATAAGGTCACCCCTCAGTCTCCTACGCTCCA

GGGAAAAATATCCCAGCCTATCCTTAGAACTCTCCTTATAACTCAAACCTTCCAGTCCAGGTAGCATCCC

AGTAAATCTTTTCTGCACTATTTCTAGTTGAGTAATATCCTTTTTTGTAGTAGGGTGACCAGAACTGCAC

ACAGTGCTGCAAAATTGGCCTCAGCTGCATCATGATGCCCCAACTCCAATACTCAATGCTCTGACCGATG

AAGGCTAGCATTCCGAAAGCCTTTTTCACCGCCCTGTCTACCTGTGACTCCACTTTCCAGGAGCTACACA

CCTGTATCCCTAGATCTCTTTGGTCTATAACATCCCTCAGGGCCTTACATCGACTGTGTAAGTCCTGCCC

TGGTTTGACCCACCAAAATGCAACGCCTTGCATTGATCTAAATTAAACGTCATCTGCCATTCCTCAATCC

CACTGGATCAAGATCTCGCTGTGTTCTCAGATAATCTTCTTCACTGCCCACTCTACCACCAATCTCGGTG

CCACACACTGCTGTGAGGAGACCAGCTTGCTTGGTTTTTTCTGTGTGTGGTCTTAATGGAATGTAAAAGC

CAACAAGTTAGGCTTCACCAGAGTGTTTTCCAAATGGTGACTGACTGCAGAATCTGGGTGTGAAGGTCAC

TAAATGCAGGTACGGTAAACGCTGGAGCGTGAGTAAGCCATTTGGCCCTTCTCCCCTGCTCTGCTATTCA

ATCCGATCTAATGCTGATCATCCAACTCAGTCCCCTATTCCCCGCTTTCTCCCTAAGAACTACATCCAAC

TCCTTCTCAAAAACATTCAGTGTTTTGGCTCCCAGCCACTTTCTGCGGCAGAGAATTCCACAGATTACTA

CCCCGCCGCCAACGCTCTCTGGGTGAAGACAATTCCCATCTCCGTCCTACCCCGTATCCTTAGACTGTGA

CCCCCTGGTTTTGGGCTCCCCCCAGGTCATCGGGAACATCCTTTACCCTGTCTAGTCCCTGTTAGAATTT

GAAAGGTTTCTATGAGATCCTCCCGCCCCAATTCTTCAAAACTCCAGTGACTATAATCCTAATCCGATCT

GGTCTCTCTCCATCCCTCAGTTCCAGGAATCCGTCTGGGAAATCCTTTGCTGCTCTCCCTCCATTCGCCA

GAACCCCCTCCCCTCAGATAAGGGGACCCTCCCCCAAACCTCCCACACACAATACTCCAGGGTGCAGTCT

CTCCGTGTTCAACTGCAGAGAGACATCCCTGCTCCTGTACTCCAATCCTCTCGCCATGGAGGGGCCCAAC

ATCCCATTGACCTTCCTCACGAAACCTCCCCCCCAGGCTGTAGGAGGGACACCTGGCTCTCGTTGTACCT

CCCCTGTTTCCCCAATCGATCGCGAGTGATTGAGAAGGTCAACGCCAATTATATCTTCTGTTACGAGAGG

AATCAGACATCAAAGTTAAAGTTGTTTCAGAGATAGTAGGAACTGCAGATGCTGGAGAATCTGAGATAAC

AGGTGTAGAGCTGGATGAACACAACAGGCCGAGCAGCATCAGAGGAGCAGGAAAGCTGACGTTTCGGGCC

TAGACCCTTCTTCAGAAAATGGGGGAGGGGAAGGGGGTTCTGAAAAAAATAGGGAGAGAGGGGGGCTCTA

TTTATTTCAGAACCCCCTTCCCCTCCCCCATTTCTGAAGAAGGGTCTAGGCCCGAAACGTCAGCCTTCCT

GCTCCTCTGATGCTGCTCGGCCTGCTGTGTTCATCCAGCTCCACACCTTGTTATCAAAGTTAAAGTGTTG

TTCTTCAGTTCCTCGAGGGGCACTGGAGAGACCACATGATGAAACTGTGTCCCATTTCGGCTCTCTACAT

TGTAGGAAGGATATTGCGAGCAGTTCAGAGCAGGATGGATGCCTAGAATAAGCGGTTTGCTGCTGACTAA

AGATCAGACGCGTCTTTCGGGCTTGTTTTCACTGGCGTTTCGAAGGCTGGGTGTTTCGAAGGCAGGGCGT

TTCGAAGGCTGGGCGTTTGAGGCTCAAAGATGGGAGTGTTCGGGTCAAAACCCTAATCAGGACAGCCACA

GCAGTACAAACCTACCTCCTGGTTTGTGTTCATACAGCGCAGGCCCTTCGGCCCAACCAGTCCGTGCCGA

CCCTAATCCCAAGCTAAACTAGTCCTACCTGCCTGCTCCAACCTTTCCTATCCATGCACTTATCCAAATG

TCTTTTAAACGTTGTAACTGTACCCACATCCACAACTTCCTCAGGAAGTTCATTCCACATGCGAACCACT

CTGCATTAAAAAAAACAAATTTGCCCCTCATGTCTTTTTTAAATCTCTCTCCTCTCACCTTAAAAATGTG

CCCCCTACACTTGAAATCCGCTACCCTAGGGAAAGGACAGCTACCATTAACTCTATCTATACCCCTCATG

ATTTTATAAATTTCTATAAAGTCACCCCTTACACTCCAGTGGAAAAAGTCCCAGCCTATCCAGGCTTTCT

TTACAACTCAAACCCTCCATTCCCGGCAACATCCTGGTAAATCTCTTCCGAACCCTCTCCAGTTTAATAA

CATCCTTCCTATAACGGGGCAACCAGAACTGGACACAGTGCTCCAGAAGAGACCTCACCAATGCCCCTGT

ACAACCTCAACATGACATCCTATAGCTCCTCCGATCTCTCCCTCTGGAGTGAACTTCCCTCATTCCTTGT

GAGAATTTCTGACGTCATTTATTTGGAAGGAGGCTAACGTTCGGAGGGGGGCTTCTGAGAGCGGGGAGTC

AATCCCTCCCTGCTTCTCTAAGGGACCGTGGTAGACGTGTCTCCGTTAGTCAGTGAGGGTGACTAATTAG

GGCTGCACAGTGGTTAGCACTGCCATCCTACAGCGCCAGGGACCCAGGTTCGATTCCAGCCTTTGGCACC

TGAGGTTTGGAGATGGGAGAGTGGAGTTTGCACGTTCTCTCTCCTCCCCACCGTGTCTGCGTGGGTTTCC

TCCGGGTGCTCCAGTCGTCTCCAAAGATGCGCAGGTTAGGGTGGACTGGCCATGGGAAATGCAGGGTTTA

CAGGGGATGGCTCTGGGTGGGATGCTCTTCAGACAATCAGTGTGGACTTATTGGGCCAGATGGCCTGTTT

CCGCGCTGAAGGGATTTTATGATTAAATAGAAGTTGCTGGCGGGATTAGGAAACCTTTTCCTGTCTGAAA

TCAGCGTTGACTTGTTCAGAAACTGCCTGATTCTAGTCAAAAGTGGTGAACGAATATTGTCTCACTGCTG

TGTGGTACCAGTTTGAGGATTGGACAATTCATGCTGCCAATCCAAGTCTGCTTGGATACATGGCCCTGAC

CGCTCTCCCGATGGAATCCTCCATCCATTGTCAACATGATGAAAGAAAAGACCAGCAAAGTGGCACATGA

ACTGCTGTACACATTTTTATTATGGTTGACCTCAGTGCCAGGCACACTTGCTTCAATATTGATATTTCTG

CTTTTTTTTTAAAAAGTCAGTATATTTACAAACTTTACACTAGAAGTGCATGTTAAAAATCAAGTAACTA

ACGTTGGAGTGCTTTATTCTGTCAGGAAGCCTGCCCACAGATGGCCTAGTCGTCAGGCTTCAGGCCTTGT

GTGAGGACAGAGGGAGGTAGTCTACTCCACTAAGGCCTTGGGAGTTAAAGAGAGCGTTCGGTTTTAGACG

GGGCGGGGAGGCTGTGAGCGTGGTTAGTGGATGTGTCTGACAGGGACATGATTGACTGGATACATCTCTG

GGCCTGGAGAAAGCCTTGCCTCTTTTCCCTCTCGGCCCACAAGCCGCGCTGCAAAAAAAAAATCAAGAAT

GACCTGCTGGATTCCGCGGCCCATCTTTTTGTGTTTTGTTTTGGAGGGGTGGTTGCTAGGGGTGGGGGGG

TTGAAGATGGGTGGCACAATTGCCTCTCTGCGTGTAACCTTTGACCCAGCGTCCACGTCAGAGCAGAACC

AACAAAGTAAGCAAAATGCAATTGCTTAGTACCGAAAGGAATCATGAGGCGATGGCCTAGTGGTATTATC

ACAGGTAAAGTTCTGGGGACCTGGGTTCGAATCCTGCCATGGCAAACGGTGGAATTTGAATTCAATAAAA

AAAAGTCTGGAATTAAGAGTCCAATGACAACCGTGAATCCATTATCGATTGTCGGGAAAAACCCATCTGT

TTCACTAACGTTCTTTAGGGAAGGAAACTGTCATCCTTACCTGGCCTGGGCCAGGTAATCTGGTTGACTC

TGAACTACCCTCTGGGCAATTAGGGATGGGCAATAAATGCTACCTGGCCAGCGATGCCCACTTCTTGTGA

ATGAATTAACCAGGGGAAAAAAAGATTTCTCTGCCGCTGCTGTGGGCATAGTCTTGTTAGGTTGGAGGAC

AGATCAGCAAACCATCATCTCGCAATGGCTGCTTCCCCACATAGTCCCCTTCCAGTTTAGGATTGTAGCA

GCAAAATAAAGATACATTCACAAAAATATTTCAACCCGTTCGGTTCCGAGTTTGATCCCAGGCAAAGAGT

GGGGGCAATAACTGGGGGGGGGGGGGGGAWCTCTTGCGCGCGCATCCGAGATTTCTACTGGAACTGTACA

TCCTCCAATTTTGGGAAGAGCGTCGAATGGTGAACCTTGGAGGGGGTGTTTGACAAGCTGAGTCTGGTGA

CAAAATGGCCGCATGTTGCCAAGGCCTTGCGCGCATCAGGACAAATGCGAGAATTCCAGGGTTCAAACAA

GAGAAAGGGTTGGCAAAGAGATGGTCGCACACTTCCTGACGAACCCCGAGTGCGCCAACAGTGAACAAGT

CTTTAGGACTTGTGCTTTTTCTGAATCACCCAGGAGCTTGGTGGAGCTTCTCCTTGGCCATTGGCTTGCC

AACCGATTGGCACCCTTTCTGTCCTACAGCGCAAATGGTTTCGCTCCCTCGCGATTAGCGCGTGTAATGA

TGAGCGCGAGTCTAAGAAGCTACGGCAGCTGATGAGAGAAGCCAGCTTCTTCGCGGGAATTTGAAGCGGG

GCTGAATTCTCAAATCACGGCTCGTTCTTTTTTTTTWAAAAGGGGCGTTTACCGACCAGGTTACGGCTTG

AAGGTAAACTTTTCCATTGAGGCCTTCAGGAGGGCTTTGGAGGATTACCCATTGGGAAATAAAGTGCTAA

GGGACGTTTGGGAAAAAAAACTGAAGAAACGGCGCCAGCCAGCGATGCTCGTTTGAAGGGGTAGCACGAG

AACGACGGGCCGAATTCCTCCATCCGCGCTGTAAAACGTATGCGACAGAGTCCGTTTTGGTTTTGGCTCC

TTTTTCTGCGGGCACAGGAGGTCAACATAGATTAGGTTACGTGCTAAGTATGTACTCAACCGGGGATCAT

TCGTCGCCAAGTGTCTGGCTTTGCCAAAAGATTGAACCTGCACATCCCAGTGAGATTTTGTTTCAGGCAA

ATACTTTTTGTTTGTTTGTTTCTCTTGACAACGAAGGGGTTTGCTGGAATTCTTGGTGGAATCCAACCTC

GCGACACGGTGGGACACTCAAGGAAATGGCAGCACTTTGCTCTGACTGAAGCCAACTGATCGATCGTCTT

CTGCAGAGACCCTTGGGTCTCCATTCGTGACCCGGTGGGAGCCGCGTTGTGTATCCAAGCGATCCTCTTG

GCAACAGGGGTGGGACACAACGGCGTTGCCCGTTTCACCCGAGGCTGCTGGGTCGATATTTTTAATGCCC

TGTTCCCTAATTGTCCTCCTGGAAAGGAGAGAGGGAGGTAGCGTACGCCAATAAACTCCTTTAAACTTAG

GCCATAGCCTAATCGTTAAAAGTGACCACTGTTTTTTTTTCTTACACGTTTCGAACCTTAAATTAACAAG

CCAGTTTCTCTTTTGCTTCTGCGCTTGACAGACAGTAAACAGATGTTCTTGCTAATATTTTGACTCAAAG

AGCTGTTGGGATTTGGAACGCGCTGCCTGGGAAAGTGGTGGCGCTGGATACATATTTGAAAGGGATGGGT

TTAGAAGGCTATGGAGATAGGGCCGGAGAGTGGGACTAGCCAGGGAGCTCTGTCGGGAGCCGGAACAGAC

ATGATGAATTGAATGGCCTCCTCTGTACTGTCACATTCCATGATTCCATTTGTGAGTTATTCCAGAGGGG

CCACCGAGCAATGTGTTGATGAAGTGGAGGCTGGTCGCTGGGATGATCTCTTCCTTCTGTCTGTTCCCCA

CCTTCCGTATCCTGTTTCCACACTCTCCAGGGCTAACTGCTGGACCAAACTCACACTCACACTCACACAC

ATACACACACTCCTCACACACACACAATCTCTCTCTCACACACACACATATACACACACACACACACACA

CACACACACAATCTCTCTCTCACACACACACATATACACACACTCCTCACACACACACACAGACACACAC

ACACACTATCTCTCTCTCACATACACACATATGCACACACTCCTCACACACACACACACAGACACACACA

CACATACACACTATCTCTCTCTCACACACACACATATACACACACTCCTCACACACTCACACACAGACAC

ACACACATACACACTCTCTCTCACACACACACTCTCTCACACACACACTCTCTCTCACACACACACACAC

ACACACACACACACACACTCTCTCACACGCACAGACACGTACACACACTCTCTCTCACACACACTCACAC

ACAGACACACTCTCTCTCTCACACACACACACAGACACACACTCTCTCTCTCACACACACATACGCACAT

ACTTACACACACAGACACACTCTCTCTCACACACACACAGACACATATACACACACTCTCTCAAACACAC

TCTCTCTCTCAGACACACATACGCACATACTTACACACACACACTCTCTCTCACACACACACACAGACAC

AGACACTCTCACACACTCTCTCTCACACACACACTCTCACTCTCACACACACACTCACACACACACACAC

TCTCTCTCTCACACACACACTCTCTCTCTCACACACACACACACCCTCTCTCTCTTTCTCATACACACAC

AGCCACAGACAGATACACACTCTCTCACATACACACACATTCACAGACCCACTCGCTCTCTCTCACACAC

ACATATACACACACACACATACACATATACACACGCATACACGCACACACTCACACACAGACAGAGGGAG

AGTGTTTTGGTGGCACCATGCCCCTTTGCCCTCGGTCACTCCCCTCCGGGGCTGGGTAGCTGAAAGAGCG

GCTGTGACCTCCTCAGCTTTGGAGGGGGGGTCGGCGGCTGCGGAACCCACTTCTCTGGCACGTGCGGGGC

ACCCTGCAAAAAACCGAGAGACGTGTGTCAAAAAAAGGGCAGGAAAACATCCTGCCTGGGGTCCCGCTGT

GTCTCCGTCTCTCTCCCTTCCTTCTCAATCCCCTTTCTATATATCTCCCTCCATTCCCTCCCCCATCTCC

CTTCTGGCTCATCCCTCCAAGTCTGTTTCCCGTTCTTCCAGGCCTGTCTTTCGATTTCACCCTTAGCGAC

AAATCCTCTTCGATGTGGGCTGGGGTTGGGAGTTGGGCCCTGGCACGGAAACTGCGGACTCCCCCTCCAC

TCACTTCTCGATAGCTCCCCACACAAGAACAGGGGCGTGCAAAAATGCTTTCTCAATCATCCACCTTGTC

AACTCCCCTCAGAACCCAACGGGCTCCAATCAGATTACCACCATGTTCCTCTGAACCTACCCCCCTGCCC

CCGGGCACAGGCCCAGCCATGCCCCAGCTGAGCGAACCATCTCTAGTGCCAATGTATCCCTCCTTAAAAC

AAACAGAACAAAACTGTGCCCTGTATTGCAGAGATGGTTTCCTCTCTGTCTACAGTGAAACATCCTTCCT

TTAACATTCCAGTCCCTTTTCAAAAGTGACCGACTTTCCATTTTGCTTTCTAATCACTTGATGCTGCTAC

AACTTTTTGTCGCTAATGTTTTGTTCCTAGCTTTCTTGTTGCTAAATTTTGGTTACTTTGACTAGAGGGT

TTGGAGAGGTTGGATAAGACTGGTTTCACTGGAGGACCTTAATAGAGGCTTATGAAATTAATTATAAAAA

TACACTTTAGGCGAATAGCAAAAAGTCTGTTCCCTAGGGTGGGGGAGTTCGAAACTAGGGGGCATATTTT

GCAGGTGAGAGGAGACAGATTTAAAAGGGACCTGAGGGGCAGCTTTTTCACACAGGAAGGTTCGTGCGTG

GAACGACCTGCTAGATGAAATGGTGGGTGCGGGTACAATTTCAACGTTTAAAAGACATTTGGACAGGTAG

ATGAATAGGAAGGGTTTAGAGGGTTGTGGGCAAAATGCAGACAAACGGGACTAGTTTAGTTTGGGAAACT

TGGTCAGCATGGATGTGTTCGACCAAAGGGTCTATTTCCGTGCTATATGACTTTATAAATCTATAACGTA

TACTAATTTTTTGTTACTAACATTTTGTTACTAACTTGTGCTAACTTTTCGTCGCCAAATTTTTGTTACT

CCTTTTTTGTTATTAACTTTTTGTTACTAATTTTCTGTCACCAACTTTTTGATACCAACTTTTCATTGCT

TTCTTGTAACTAACTTTTTGTTGTGAATTTTTTGCTGCTAGTTTTCTTTCTAACGTTTCGTTGCTGACTC

CACGTATCAAGACTCCCAAGTCCCTCTGTACCACAGCATTCCGCAGTTTCTCTCCGTTTAAGTTATAATC

TGCTCTTTTCCGTCGTCCCTCACTGACCAAGTTCACAATTTCTTGCATTGCGCTTGGCAGGTTCCTTTTC

CCTCCCCTCTTGGTACGTTCTTGGTATCAACACGAGAGAAAGCGTAGCTGTTGTACAGGTCCCTGATGCA

GACTGTAAGTAGGGGAGTCTAGCTCCTCTCTCATCACCCCCGCCCGTGCACACCCCAGCACTGACACTCC

TGTCTCCTCTCACGCGGCACTCAATTACGTGGCCAGTTTGCGTGAATCATCCTCAGTGCTACCTCGGTCG

TATCCCTCTGGCTCCCATTTTCGTAATGTTTGTTCTTTGGAGGGGGCCGCGGGGGACTGTCACAAGAACC

CAGTGAGTTCTCCTGGTCTCTCTGCAGCGCGGGGTAACCTGTAAAAGAGAGACAAGGGTCAGGCGAGATC

CAAAATGAGACCACTGCGTCGGACACAGGGAGCAGATCAGGCAGAAATTAATGGGACGTCGATCGCCATC

CAATTTCAGGGGTCAATGGGTGCCCTTGCTGGGAGATGGTCAATGCCTGGCACTTGGGTGGCGCAAATGT

TAGTTGCCACTTAGAAACGTCAGAGCAGGGGTGGGCCATTCAGCCCTTCGAGCCTGCCCCACCATTCAAT

ACAATCATAGGCTGATCATCCAACTCAGCCCCCTTTCACCCTTCGATCCCTTTAGCCCCAAGAACTATAT

CCAACTCCTTCCTGAAAGCTTCCGATTTCTGTGGCAGAGAATTCTACAGACTCCCCCCAACCCCCTAACT

CTCTGGGTCAAAACATTTCTCCTCATCTCAGTCATATCACCGTATCCTTAGACTATGACGCTCTGGTTCT

GGGCTCTCCCCTGGGTCATCGGGAACATCCTTTACCCTGTCTAGTCCCTGTTAGCATTTGAAAGGTTTCT

ATGCGACCCCCCCCACAAACCCGCGCCCCCCCCACCAATTCTTCTAAACTCCTGTGAATATAATCCTAAC

CCAATCCAGTCTCTCTCCGTCCATCAGTCCATGCCATCCCAGGAATCTGACTGGGAAACCCTTCGCCAGA

ACCCCCTCCCCTCAGATAAGGAGACCCCCAAACCCCCTACACACAATACTCCAGGGTTATGTCCCACTCT

GTCCAATTGCAGAGAAACATCCCTGCTCCTGTACTCCAATCCTCTCGCCATGAAGGGACGAACATCCCAT

TGGCCTTCCTCATTCCCTACTGCTCCTGACTTTCAGCGACTCGTGTACGAGGGGACGCCCAGGTCATGTT

GCGTCTTTCTCTCTCTCTCTCTCCCAACCGATCGCTATTTGGAATAATAACCACCTCCCTCTTTCTGCTC

CCAAACCCTACTTGCCAGCTCAGGCCTGGGAATATTGTCCAGGGAATATTGCTGCATGTACAATCCCTCA

GTATCTGTAGCGGCGCTAACGGGTTTGGATATTCCAAAATTGCCGGCGAACATCCCACCATCGGAATTGT

GATGGGGGGAGGATCCCAGAATGTTCTGGATGGGAAGAAGAGCTTTTATTAAGGGAGAACATCGAGAGAA

AAAAAAAGAACACGCTGAGTAGGCCGGGACGATGAAGAGGAATTTTTAGCACTGCGCGCGCAGCCTCGCC

TTCGCGAGTTGAGCCAGCCATTACATACTCAGAGTCCGCATGGTGTCTGCTGCTGTGATCTGCGTTGGCA

CCGGCTGCGGGGCGTAGATTTGCATCGAAGACTGGCACCGATCGCCAAACGGAAGACTTTTTGGACGCAG

TTGCCTATGTCTCGCCACCTACACGACCAGAAGACGGCGACTGTGACTCTCCGGGGGAACAAGAAAACGC

AACAACGATCTCTGACGCTCTCGACACCTTGGGAACCAGGCGAGCGTCACACCCTCACCCATCCCCTGGC

AGCAACACCGCACCACCCCCCACCCCCTGGTGTTTCCTTCCACTCTCTCTCTCTCTCTGCACGTGTGTGT

CCCTCAGAGTTGCGCTCCCGGGCACTAACGCCGCACGGAAATCCAGTCATCCCAATGGATCTTTTCTTTA

AAAGCTGAATTCCCTGGTTAGGCTGGGAATTGTTGCCCAGACTCCCAGAGGGCAGTTAAGGATCACCCAC

AATGCAGTGGGGCTGGAGAGCAGAATGGCCAATTTCCTCCCCAATAGATATTAGCGAACCAGAGCGATCT

TTCCAACACGGGTCATCAATTTTGTTTTTAACTCTGGAATTATGTTGAGTTCAAACTTGACTATAAATTC

TTAAAAACCCTTAGGAGCGCTGATATACAGAGGGATCGTGAGGCGCATGTCCCTTACCCCCCTCTGTAAG

TGACAACACAAGTGGATAAGGTGGGAAAGAAGGCGCATGGCAAGTTTGCCTCCATCGGTCGGGGCTTTGA

GTAGAAAAGTTGCCAAGTCATGTTGCATCTGTGTGAGGCTTTGGTCAGGCCACATTTGGAGCATTGTTTG

CAGTTCTGGTCGCCACACTATAGGAAGGATGTGGGAGTTTTGGAGAGGGTGCAGAAGAGGTTTACCAGGA

TGTTGCCGGGATTGGGGCGTATTAGCTACAAGGAGAAGTTGGGCAAACTTGGATTGTTTTCGCTAGAGTG

TCGGAGGCCGAGGGGAGACTTGATAGAAATCTGTCGAATTATGAGGGGCACGGGTAGTCAGAATCTCTTT

CCCAGAGTAGAAATGTCAAATACTAGGGGGCAAAGGTTTCAGGTGAGAGGGGAAAAGTTTACAGGAGATG

TGCGAGGCACAATTTTTTATGGGTAGGTGCCTGGAACGTGTCACCATTAGTGGTGGTAGAAGCTGATACA

ATGGTAATGTTTAAAAGGCAGACACATGAACAGACGGAGGATACAGGGACCATGCGCAGGGAGATGGGAT

TGGTTTAGAATGGGTCAGAACAGACACGGTGGGCCGAAGAGCCTGTTCCTGCACTACACCAAGCTAATGT

TCTATCTGCTCTGGTAGGATTTAAACTCACGTCCTCAGAACGTTAACTGGATTTCTAGTCCAGTGACAAT

ACCATGATACCACCACCTAAAACCAAATAAACTGAGGCATTCTGTCCTGTCCTCATCAGGACCCTTGGCA

AGAGTGCCAAATTTCAAACAATAGCAACAAGTCGATCCGCGGGAGAAACGATCCGGTTGTCCGAGTGTTG

CCATGGAAGAAAAAGAAACATTAGCAGGCCACAGGCTCCCCAGCTGTTTAAATTGTTGCGATGGTCGGAA

ATTTGACACTCTTGCCAAGGGTCCTGAGGAGCGCAGAACGGAAAAACCTCAGCCATCAGTGATATTGTAG

GTAGGTAACATCAGGGATTGAAAGAAACAAAGTGGGCGACGCCAACAAGAAACAAACGCCAAAGCAAACG

GAGGAAGTGATGTACGTACCGTTTCGGCCATGTTGGGTGAGAGGTCGCGCCTTGTTTCTTTCTCAAAGAT

GACCTGCGATTTACTGAGGTTCTGTTCTTTCCTCCTGGACTTATTCGCTCTGTGGTTCTGTTCGTCTTTG

CCTGGGCTCCCATCCATCTTGTGTTCGAGCAGTGGGTCCAAAACGGAACTGCAGAGACGCGACCAAGATT

CTGAGCTCAGGTCCCCGTCTCAAAGTGGGCCCCCGTCCTTCTGAGGAACACGACAGGGAAGCACCCCCAC

CTGCCCTCCCCCCACCACCCCCCAACCTCAGCACTGCACGTGCCTAAAGCCGGACTGGAGCTCTGCCCCT

GAGGCTTCTGGGAGGCAGTTTGTGACCCCGAGAGGTGACTCAGACCTCTTCTGATCCAGGCTCCTCCCTC

CCTATCCCGTAAAGTCTGAACCACCTTCAGATCATGAAGGCACTCAAAAACGAGACCCCTACAAGCCCTC

TTGGTGGGGGAGTGGTGTGGGGGAGAAGGAACCTTCTTCACTTGCCTCTTCATGCCCATCCAAGATGGCC

GACCCCTCAGTCTGCGACCCCTGACCCCTGTGTCCAAGATTCCCCAGCCAAGGGATCGGGTAGCCATCAC

TGTCAGCGCCTCCCCTTGAGAGTTTTAACGAGATCCCTTTCTTATGGGGAGATCCATGAGGGTTGGAGCT

GATGTAGACCATTTGGCCCATGGAGTTCAATCAGGTCTGAGCTGATATTCCCCCACGTTCCCTGCCTTTC

AGTCCTCGATTTCCCCTTCCTGACTGAAAACCCATCTCGGCCTTGAGTAGACTTCACGACCCAGCTCCTA

CAGCCCTCTCTGGTAAAGAATTCCACACATTAGCTCCCCTCGGAGAGAAGAAATTCCTCCTCATCTCCAT

CTTCAATGGGTGACCCCGTATTCTGAGATTACACCCTCTGGTCCTAGACGCTCCCACAAGGGGACACAAC

CTCTCTGCATCTCTCCCACCCTTGACAGGTCCTTCATTAACGGGATGAGGGACGTCGCTGGCCAGGCAGC

ATTTATTGCCCATCCCTAATTGCCCAGAGTTCAGAGTCAACCACATTGCTGTGTGGGTCTGGAGTTACAT

GTAGGCCAGTTTCCATCCCTAAAGGACATTAGTCTCTCTAAGAATCTGTCTGTTTCAATAAGGTCGCCTC

TCATTGTTCTAAACGAGTACAGGCCCCGGCCTACTCAACCTCTCCTCAGTCCCTCCTTACCGGGATCAGC

CGTGTGAACCTACTCCAGGCTGCCTCCGATGGCTGGTCCATCTCTGCTTGGATACGGGGTCCCGAAACTG

TTCACAGTATTCCGGCTGCGGCCTTAGGTGGAGAGTCGGGCCTAATGATGGGGCACTGGGGAGGTGGAGG

GAGGGGGCGGCTGGGGCGAGAGGAGGGCGGGTAGTTAGGGGCCGTGGCCTCCAGTTGGACCCCTTTTGTA

GCCCCCCCCCATCTCAGGGACAATTGGGAGGCAGTCGGGCATTCGATCTGGGCAGCCTCGGGGTCGCTCG

TCCAGTGGAAAGACCCACCCCTGCCCCCCGCCCCCCCCACTCCCACCACCACCTCACTCGCTGGGAGCCA

ATGGGCGAGATGGAGGCCCAGATGGGGTGACTACCACCCCTCCCCACACCCCAATGTCCCCTGCTGACTG

GAGGCCGCACCTACCCGTCAGATGTGGGGC…

B. Whale shark GnRH2 gene

**NW_018034174**

…TAAGTCATTCATCATCTCTGCACAAACTCTCACAGGCACTGAGTTCCAAGTCATTACCGCTCACCGTAAT

AATAGGCTCTTTCTTCCATCTTCTACGTCTCTCACCCAAAACTTTAAATCTGTGTCCCCCCAGCCTTCAT

ACCATTAACTAATGGGAGCAGTTTATTCTTGTCTACCTTATTTAAACCTGTCATAATCTTGTACATGTCT

TTCAAACCTCCCCTCAATCTCCTTTTCTCTGAGAAAAATTACTCCATCTTCTTCAACCTAATTTTATATC

TGAAATCTCTCATATAAGCCCTCATCCCTGTAACTGATCTGGTCAAAGTGTGGGGAAGGTGATACCTGGT

TGGACTGCACCTGAACAGGGCCAGGATAAATATCCTTGCAGGGACACTTGTTAATGCTGCTGGGAAGAGT

TTAAGCTAGGTTTGCCAGGAGAAGGAGAAGCAGGGGCCAAATTCCAAGCAAGGAGGAGGAGGCAGAAACT

TAACAAGTGACTCTGAAAAACAGAGGAAGTAGAGGTTAAAAATATCTGAAAGGACAATGGATATGGCTTC

AATACTAACCTTCAAAAGGCAATTGGACAAATGCTTGAAAGAGGAAAAATTGCAGGGTTATTGGGGAAGA

TGGCGATCTAATAGGAATTCTCTAGGAAAGGACCAGCACAGACTTGAACGGCCAAAATTGCCTTCTTCTA

CACTATTATATTTGATCATCTTAGGTATGGCCTCTTAAAATTGTCGGTGTAACTGGAGATAATCTTTCAG

TTTCTTATCAATTACAGAACCTGGCTTATTTTTTTCACCAGTTACGCAAATCAGATATAGGAGGCTCATC

CACAGCAACATA**ATG**GCTTTCCAGAGAAACCTGCATTTCCTGGTATTCCTGCTGCTGATTGTTAACACTG

**M A F Q R N L H F L V F L L L I V N T**

AGTTTTCCACAGCCCAACACTGGTCCCATGGTTGGTATCCAGGAGGGAAGAGAGAAGTGAGTCTATCTCA

**E F S T A Q H W S H G W Y P G G K R** E V S L S Q

ATCTCCAGATGTGAGTCCTACTCTCTCAGCATTCTTTAATCTTTGCTAAAATCCTAACTGCTTCCCGTGA

S P D

ATGTAACAACCAAAAAGGAATTGACAACTCTTTACTTATTTATATTTATTAGAATATGCAACATAGTGAT

ATTCTCAAGGGATCTTTACTTCCAGTTGAAAGTCCTGGAATATAAATACAAAATTCTGGAATTGCCCATT

AGGGTCAGATAATGTCTGTGGAGACAGAACGGTTAACATTTCGAACCAATGACTTTTCATCAGAACTGGA

ATAAGTCAGAGATGTGATGGTTTTAACCAAATGCAGAGGCACAGAAATGTGAGAGGGGTTAACAGCTCTG

AACTTTAAAAATTGGTCATATCATCAACTTGCTTAAATTTAAAATTTATTATGATCGGTCAACAAGAGGG

ACTAGAAAGAAATTGGTAGACCCCATAACTTCAATTGAAACGGTATGACCACAGTTCTGTTAGGAAAGAA

GTTCTAGGATAGTGACATTGAAGGAATGGTAATATATTTCCAAGTCAGGATGGGGAATGGCTTGGAGGGG

AACTCCCATGTGTCTTGATCCAAACATGGACAAGGGAGCTGAACTCAAAAGGCAAAGTGGGTTTGATTAG

TCTTGACATTAAGGTAGCACTTGCCCAAGTGTGGTATCATCAAGGAACAAAACTAAAGTCAATACAAATC

AGAGGGAAAACACTCTACCCATTAGAGTCATGCCTAGTGGTTGTCGGAGGTCAGTCATCTCAGCTCCAGG

ACATCTCTGCAGGCATTCCTAAGGGTAATGTCCTATGCCCAACCATCTTCAGCTTCTTGATCAATGACCT

TCACTCCGTCATAAGGTCAAAGGTGGGAAATTCACCAATGATTGCACAATGTTCAGCACCATTTGCAACT

CCTCGGACACTGAAGCAGTCCATGTCTAAATGCAGCAGGACCTGGAGAACATTTAGGTTTGGCTGTAAAA

TGGCAGTTAAAATTTGTACCACACCAGTGTCAGGCAATGGCCATCTTTATCGAAAGAGAGTCGAACCATC

ATCCTTTGATTTTCAATGATGCACTATCACTGAATCCACAACTATTGACATTTTAAAGTTACCATTGGCC

AGACTAGCCAGGTACATGTTATAGCAGGTCAGAAGCTGCGAACTCCATGGACAGTAACTCATCTCCTGAC

TCCACAAAGGCCTGTCCACCACATACAAGGCACAACTCAGGAGTGCGATGGAATATTTTCCATTTGCCTG

GAAGGGTGCAGCTCTAGCAACACAAAAATCTCAATGCCTCCCAGGACATAGTGGCCCATTTGATTGATAC

GCTGGCTGTCAGCTTCAGTATTCACTCCCATCATGACCAATGCACAGTGGCAGCAGTATGGCCATCTACA

AGGTGCACTGCAACAACTCCCCAAAGTTCCTTAGACAGAAATGTCCAAATGCATGATTTCTACCACCTGT

AAGGAGAAAGGCAGACCTGCCCTCCCTCCTCCCTGATTTGTTTTGCCTCTCACCCACGTCCGTTTCTGCT

CTGTCAGCTCTGTAAAATTCTCCGGAGCTGAGATGACTGACCTCCAATAACTACTAGGCATGACTCTAAC

GGGTAGAGTGTTTCCCCTCTGATTTGAATTGACTTTAGTTTTGTCTGAATTCTGCAGACAGGGGCTAGTC

AAGACTCAATATAACTGAAGTAGTAAGTCAGGCTTTTTTACTCCAAACCTCTTAAGGTAAAGAAACAGGG

ATAATTACTTCTACAGAGGAGGTTAAGGGGTTGAAGGAGGACTAAACGGTGGTCGAAGATAACTGTATTG

CACAAGTTCAATTGAATGCTCCTTGTCATTTCCTAGGCTTCAGAAGAAATCAAGTTATGTCAAGGTGAGG

A S E E I K L C Q G E

GTTGCCTGTTGCTGCGAAGTCCACGTAGAGGGATCATAAGGAGCATTGTGGTAATTAGAACATTCTTATT

G C L L L R S P R R G I I R S I V

TTTTTCAGGAAATACGAATAGTGGCTTGAATAAACAATATAAAAATTCCACCTGGTTTTGCAAATAAGGG

AAGACATTGTGAAAATACTGCATAATCCCACAAGGGTTACAGTACCATTTTGTTAAATTTCAGAGGCATA

TTTGTCAATTATCAGATTTTGTTTTTGTTGAGTTTCTGCTGTTCAGGAATTAAGTACAAGGGGCTGCAAT

TACATGGAAGGACTTGAGAAATTGAGATTCTTAGAGCTGAGAAAGTTAAGTAGTGATTTAACGCACATAT

TTAAAGTTGTGAGAGTTTTTGAGAGAGTATGGCAAAACTAGTTCCAGTGGTAGTCTGATTGGTGACAAGA

GGATGCAGATTAAGGTAATTGGCCAAAGAATAACAGGGGAGATTCACCAGCATTTTATCATCTGCCTGAA

AGGACAGTGGAAGTAGATTCATTGGTAACTTTTAAAAGTGAATTGGATAAATACTTAAAAAGGGAAACTT

TTCAAAGTCATTTGTGAGGCAACATGGAGGTGAGATTAATGGGATCGATCTTTCAAAGTGGGCCAGACAT

GAAGGGCTGCATCACTTCCTTTTGTGCTACATCATTTAGTGAGTGTGTACAGTTAGGCCAAAATCAGTGC

TCATTTCCTGTGATTCTGGATTTAAAGGTTATAGCCAGAACAAGAGTTCTATTATTTAGAACTGTGGGTG

TGCAGGGTTTGCAACTGTCCTTAGGAAGATTATTAATGATTTTTATCTTGAAAGTGTAATGTTACATCCT

CAAAGGACTATAGAATAACTCTCTCCATTTGAGAGAGAGAGATAGATGCTTATGTAGGGCTTGAGGTGAC

CATTCCACAAGGTGATAAGGCAGGTGTCTTTGCTGTATTATTGCTGGTGCAATGAATGAATCCATGCTGT

TGCCTTACCACACTGTAGTGATCTAATCAACTGAGCTTTACATTGAAAGTAGATCTTAAGCATATCAAGA

AAGCAGCACTATTCTGGATAAGTATGAAATTATAATGAGTTTGTTCATTCTGCTCTTTTATATTCCTGTC

TCTGTCGTAAAATCAGATGGATATGTTGGTGCAACAGATTCAGAAGAAGAAA**TGA**AGTCTGCCTTGTTTT

M D M L V Q Q I Q K K K -

CAGATTGCCCCCAGTGTAAATTATAAGCTCATGAAGCTGATGTGCCTGAAGATATTTTCCTCAGAAATAT

GATTTTAAATTCTGCAATGAGTTTCTGTCAAAGGAAAGCTCATCTCATGTACTTGGCTTGAAGACTATTA

AATGGTGCTGTGAGGCTGTTTGAGTGACATCTGTGAAAACACTGCAGATAGACTTCCCTTTCTGGCATTT

TAATTTCAACCTTGTGAAATGAAGGACTTTATTTTGCCATGTATCGATAACACCAACTCTCAAATGCCAT

TGCTTGAATATGAGAAATACTCAAGAAAAGAGATATAAACAGTTGGTGTGTTTTATTTAATAACTGTTTT

TCCAAGCTTTGGCATTATTTTGCCTTAAGCTAAAGCAACTGTACATGTCTGAAAATCTTTACAAATTATC

ATTTCTCTCTATTTTGCATTAAAAGATGATTGTATTCAATGTGTTTCAGCGTCAAACTTCAGGTAACATT

TCATGGTTTTGGGTTCAGGGTTCCTCCGAATGGACACATTAATCGGATGGGAAAATGGTCCTGGTGTCAC

ACGTGGGATCCATGGCACAGCTCTCTACAACAGGGGAACAGAATAATAATCGATGTGTACTAAAATAACC

ATCTCCTTTAATGATTACAAAGGATCTATGAAAAGCACCTAGAATACATTTGCTCCAAATGAGCTCACAT

AAGGAAACAATAATAAATAGGTTCATGCAAATCATTAAAGCTGGGAAAAAGGACAAGCTCATGAAAATTA

TTCCCATTTCAGCATTAAATTCCAGGCTGATTGACAAGGGATTTCACTGCTGTAGTGTGGAATGTTGTAC

ATTCAGTGACACTCAAGAAAGGTCTAGCAGAGCTTCCTTTTCTGTGTCCAACAATGTCTTTTTAAAATTC

TTTCATACAATGGGTGGTGTTAATGCCAACATTTGTTTCTCATTCTTCAATACCCTTGAGAAAGTGATGG

TGAGCCACTTTTTTGAACCATTGCTGTCTTTCTGTGGTGAGAAAACTTGAATAGGTCACTGAGTCAGCTC

AGTGAGAAGCTAAGAGTTAGCCATATTACCTCGGGTTTAGAGTAGGTCAGACCAGGTAAGGGTGGAAAAT

TTCTTCTCTACATTTGTGAACGACATGAATTTTACAGCAATCGTTTAGTTTCATGGCTGCAATAATAAAA

TTAGCTTTCAATTCCAGACTTTATTAATTCATTGAATTCAAATTCACCAACTATTAGTGGTACCTGTATT…

C. Whale shark GnRH3 gene

**NW_018056427**

gcattgttgttaggaatgatttaattcgccaatggtttagacagagagctgcatgtttggttgtttgttg

ataattggttgcattaatctgtcctgtgttttgtgtaaatcacagtgcaaaatctttattttgttgaaga

atgccttgaagacaatccattgataatcaatatacttttaacacagaaaacagatattcgaaaaacgtgg

ctactggtcaaaagaataaccttccacattgacatttggaattttttatatttttaaatgagatttatca

tttattttagggccttaaaggaacaaaattctttggatgccagagaatatgcccagctacctcaaaagaa

tccaccactgttacacaatctttctggcattatccccagagttgtctcacgtattcccagaggtacacat

accacagtttaggaaccactaatatgcacatgcaaatgtgaaaatttcaagaaaacaaattggtgagtgt

ggcaggtacctaatattggagccctttggataatgttgttccataataaagaccaattttactaaataaa

aataataccttcgacttttgacgatgtgaagattgaacaaaattgtagtaagtgcccagtaaagtggtta

agtgaattgtaagctttaaagctcactttagtgttttaacttaaagtgcctttttatacaccaagggatt

tattcaggattatatattggttttaggattagattttaaaaaaatgaaatccctctgttgttgattaacc

ataccacgttaatgtgctatatatttaagtgccacaactgaaaattctattttcaagctgacgagtaaga

actctgaatgaagagttctcttgcctagaacatttttttttggtactaaataaggaatagatttgttttg

cataatttacttttcttcacttattttatagatatacctctactttacctgaaatatttttctcttcatt

tca**atg**gaagttaccaaaacaatttccatccattttctgatagcagtgatgtttattgctcatggctgta

**M E V T K T I S I H F L I A V M F I A H G C**

tctcccagcactggtctcatggttggctgcctggaggaaaaaggagtgcagtgagtatggatgcttattt

I S **Q H W S H G W L P G G K R** S A V S M D A Y L

agaggtaagacccagattttatttcattgttatttgttatattgcttacaaacacaaagattgattcagc

E

taagttgttgcatagcagttagcttgttaacagttaagccctaacctgatgaggttgttggcttgctcgc

cgagctgactggttatcgtacagacatgttgtcaccatgctaggtaacatcatcagtgcggcctccgaag

aagcgatgttgttctactccacttggtatttatatgatccggtctgttaagttggattgtgtcatttctg

gttcggttctgcatgggtttgcatatggggtctgattccacatgtttgttggttgcattatggattgaaa

accaggcctcaaggaattcccatgtgtatctgtggttggcttgagctaggatggtcacgttgtcccagtt

aaaatgatggccttcattgtccgagtgcactatgatgaaggaaagttcatcatgtcattttgctgctagc

tggtatttgtagctggaagaggcctggttttcaatccataatgcaatcaacaaacatgtggaattagacc

ccatatacaaacccatgcagaacagtagcggaaatgacacaacccaacttgacaggcaggatcatataaa

taccaagcagagtagaacaacatcacttcatcggaggctgcactgatgatgttacctagcatggtgacaa

aacgtctgtacgataaccagccaactcggcaagcaagccaacaacccaagctacaaatcttcgcaagaac

taacttgatgttaacacactcaactcaaaaaagtgaattgtttactagtcagtgaaccatgggagatgat

ctcagccaagtttctgtctgacatcctcagatgtacatttgcaagtaccaatcatagaaatcatagtcag

ttgcacaaacccaaaccacaaatgattgtttgacagtgagtggtgctgaaaggaaacacgttttgttgaa

acatttcatcttgcactcatcagagtaattcacaaaaatactagtttaagggaaaacccaccaacaaccc

tgtatgaggcgagaatgtttgattttgaagtagggtgcagtaaagccatccagtaggctaaatggctact

cggatagatcatttcttaccatatatcatacaatttgatgattggccctaaaggctgtcacatttggtcc

tgacaagcacccagtctactcgagctcatgctcaagagtttgagcaataagcgaagatagcggtttcata

tattttgcaatagcaacgtgaggatcactccccactaacaggatgcggtgtcaaaccaaaatgatgttct

gcctattataccaatagttgtgcagtggatgactttcagtgcagttgtgatgccaggtacgttctttttt

attcattcatgggacaagggcatcactgcttggccagcatttattgcccgtctctagttgctcatgaaaa

gatgttggtgaacagcctccttgaagcattgcagtccatatcctgtaagttgacccacaatgccattagg

gagggaattccaggattttgacccagtgacaatgaatgaatggcgatatatttccaagtcagaatagtga

atggctcgcaggtcctggtgttcccatgtatctgctgcccttgtccttctagatggaagtggtcgtgagt

ttggaaggtaatatctaaggatgtttggtgtatttacatccaaagattagcatattgtaccaaacagcgt

atccattcagcagttaacaataagcaagctatggaccatactcaaccagcccatktgtccaaaatacaaa

acatagcatctaacatgattcagcaatcggacattatctagataatcaaatataattataacccaagtgt

tgaactccctacatcatcaccacatcttcacatccctcacaggtaaagcagagtataaggtcttgctrat

ggtgggagaatgataactaaattgaggaggaggcaagtgaggtggatttcttccgggttctcctttttgg

acagcaaaactttggcaaaaagtctgttttaatgttggtttatattatggacacagtgactacaatttgg

agaatctgctgaggaaattccgagcaatgttttatgttgtgagatgatgcaggtggtctgtagcctgcag

aacagcttttgattattattgtggtcctatggaagcctaagcacaacaacagtttgatttttgctgtaat

ttagtaaatatgcaacagtgtactatataactgatttgaaggacaaattaggacttggattttgacttaa

gaataatgaaaatgctctaagtgctcaaagttacaacaggcagcaaattttagtgattgcagttttgcag

ctgaaatcaagatgtttctgcctttctattcttcaagtactgctataatgttatcccactgaaatacatg

tcagaaaatttataaattgcctagtgtaatggtagccttagtgataagccttagtttgtgccgcatagtc

actctggtgctgaaaattagctttacaagtgtggcttctcatttcttcagatgttaaccatagttgaagg

tcttaaacaatgaataaattatttttaaacttttctttctgcttatattttctcctttgcttggtctgat

ttttctgtccttctcttcattatacttcacgcacatcatttgacattgaactaggatattctaattttac

atcctgatttagacttcatgtcacttacacgtgtggcacaaatctttcaagtataatatttgaaatgtta

tttgtctgtcacttcatgaaacttggtttgagatgcaattttgttccttcctccccagtgcatgttattg

aaaattactgatgtgaaacatattttaattaatttgccataaaggttttatagggcagattcttctgtgt

ttagaatgtgtctcatcattatctggaaaagtgctcatatacacacacacacacacacagataaggccta

ccatatcttttatccatcaggtacttacaaggacagcagggtgcctgcatcctgacagaggattccagga

tatgatgccccaccacctagagatgttatctaatcaaagactgacagctctcgcactcagcaatgccgcg

aggtggccattgttactgccagtgcaacatatcccaaaattgcaggactggggagcaaaccagggaaaag

gtgtatggttggaaggatctaatagtgtgggagttgtggagagacattcccatgtccttgaccatagaga

aattagtgcagatcaagtgaccacatctaagacctgatgtgccgtcatgcagttttaaacagccaaatgg

gccacatgttagcagacctatctgcccagacttactaccagcagatggcagtaatgcataccagtcccaa

tgccaacccatgtcattttcaaaccctcctgcctccttctcttccattaataatatcagcttaagaagtc

agacttcttgaaaatgctttattaatccatcctccatataaaggagcttttgcaacttttgctgaagctt

tatgatgttattattcaccttgaacagatagccaaccacccaagtccaggcctgtcttactgacttctgc

tacaatgtctgaggtcaacatcaacacacttcaaatactccagatatccccaaaaaaatttcatttcctt

tagctcaaaaattttatttgagatcaaattgctgaataaaacaatatttaattatttgggttaaagctac

ctgccttgcagtcccagtgtgtagccaaacttctgaaggtagacggcaggaaacaccataatattctaag

tcctgttcaatgcgtggtctctttgagtggaatctctcactggagcttgggactggtgaaatcctacaat

gatattctttctgattactttggccttgcagctgtcaagttatcatttctttcttgcctggttgtttagt

gatgtaggtcatgagtcccacaggagagagatgaaatctgctgcttttatacacatcacacttaggcagc

acacatacatacacagactgatgttaatccgcagtagaaattaggggtgctgtatgaagtgaggtgcaac

acctccaggttgcagcagtgtttttctgggggaatctaaccctttgtcccaactccttgcctttggctaa

ttggctgcctaaaacataaattgaacggaacatcaacaggttaggcttcaacgggataggcagagttaag

atcctattcacacagaccttatatatacacacaatttgtcactggtttgtgacttcttaaaattaagtat

aaagcttgcacagttaaataagacattattgcaaacttattgaaatatttttaacagaattatccatttc

ctatgtgtttatgtcataaatgttaattttatctgaatgtactgacctgagtgagagaggaaaattctag

tttaaaggttgaaatattgtatgtatattacttggtgtgacacggaatactattcctaatagcttttttt

ctctttgtcgaatagtttttcttacctggattgtaactaattaatatttcagctaatctgcatggctaat

taaactgattatttcaaactaattaaagtgataaaaaatacattgttaaggtatgatggacgttgtactc

cagacatacacaagaccatcaattaaacagaaaaacaaaataatgtatttgtagagcacctgatcatctc

acaaaaatgttaaaaagaatttaaaatataactaattactgttaaatgtagtttttgttattaagatata

agtggcagccattttgttgcaatcatgtatctcaatagttatacccttaaagttatcagagaaacagtat

atatgttttaaacctcaataagactctgtttgcactcctctcctgaaggcatgggaaccttcctgatgtc

taatttcacactcccaggttatgaaggtgtaactagtagagttgccagtcgatgtggtcaatttggactt

tcaaaaagcatttgataaagtcccgcataagagattattgtgcaagattaaagcgcatgagattggggga

agtgtattgagatggatagaaaactgtttggcagagaggaaacaaagaatatgaattaatgggtcctttt

caaatggcagacagtaactagtggggtgccacaggcatcggtgctgggacaccagctattcacaatgtat

atcaatgatttggatgagggaacaaaatgtaacacctcaaagtttgcagatgatatcaagttgggtggga

gggtgaactgtgacaaggatgcagagatccttcagcatgatctcaacaggttggatgactgggcaaatca

atggcagatgcagcataatttggataaatgtgaggttatccactttagaagcaaaaacaagaaggcagat

tactacctgaaaggctgtaaattgggagatgggagtgtgcagtgggacccgggtgtccttttgcactgaa

ggtaagcatgcaggtgcggcaggcggtaaagaaggcaaatggtatgttggccttcattgcaagaggtttt

cagtactgaagcagagatgtgttgttgcagttatacagagccttgttgaggtcacacctagaatattgtg

tgcagttttggtctctttttctgaagaaggatgctcttgctctcaagggagtgcagtgaaggtttaccag

gttgattccagggagggcaggactgacatatgaggagagattgactaggttatgattgttttcactggag

ttcagacgaatgaaaggggatctcataggaacttataaaattctaacaggactggacaggatagatgcag

ggaggatgttcccaatggtgggtgtgtccagaaccaggggtcacagtctgaggatttggggtagatgatt

taggacggagatgaggagacatttcttcactcaaagagtggtcagcatctggaattcgttaccacaggaa

gtagttaatgccaaaacattgaatcttttcaagaggtggctagatatagcacttggggcaaatgggatca

gaggttatggggagaaagtaggattaggctattgagttggatgactagccatgatcgtgatgaatggcag

agtaggcttgaagggctgaatggcctcctcctgttctatgttaccctctgctaattcagctgaaaatttg

cagtttttagctgtaggacttcaggaaaataaaaatgcacgggtttggatgcagaagcgtgccatttggt

tctttgagcctgttccgcaataaaatctagttgtggtctcaactccaatttcttgtctgcccttcgtaac

ctttggatctcttagctatcagcaatctaccttatcctgccttgaataaattcaatgattcagcctccac

taatttctgggtcagaaaattccactaatgtggctttgagcaaaaatatttctcctcatctccatcttac

aagggacacctcttaaatttaaactgtgttccttagttctaacctcactgcaaaagaaaacatcctctca

ggatccaccccatcaggaccactatggatcatgtatgtttcaataagagcacttctcattcttctaaact

ccaatgggtaaagttccaataatttcaacctttccttataaaataagcccttcatcccatgaatgagaca

ggtgaatcctgtctgaagtgcttccaatacaattgtatcacttttttttcaataaggagaccaacaccat

actccaaatgaggtctcaatgaggctgcagtacagctgcagtaaaatttcccctcttttatattccattc

cctgggcattaaacataaacattccatttgccttctttgtcattcgccatacttggaactaatcttttgt

gattcatgtgctagcagatctagatctttaattccttcattctgccccattaaaataaaatactccattt

atttcttaaagattttcagtatttcaattcctggaaatgggagtttgataattttgtaaaattttctttt

tttatttcagatgataaatgatgaagatgtaataactgactttgagatacccagatatcaatatttatat

M I N D E D V I T D F E I P R Y Q Y L Y

cagagggcaaataatcctcaagctattatagtaagtaactgataaaatgtatactgatgctaacagaaac

Q R A N N P Q A I I

tgaggaatgtgggaagtttccaaccctatcaaaatgggcaagtttgtgtcaaatgggaagttacattgct

aaaagtctgaatcccaattccaaatcaaccacttctggttataaaggaataggcgaccaacctcatcctg

ggtcagtaagtattataagactttatgcctcatttttttttactggcgatgttcaatttaactctggttg

gtcaggccttggaccagcagctaaataaaagcttttctacttacctcttggatttgacatgcctacctgg

ccaagtctccatgatcagacattcctccccctaccccaatcctgctgatttccccatgatcttaccttat

caagcctttaatacccttcatttccccatcaagacctcccattacttcccttccaccaggcctcagatct

ccaatctgtctccagaagcctccctatcaccttgtcttgccatttaatctccaacccttctccaatctga

tattgggttgagaaaggaaggaatcttgtgtcaacccaataccagattgctctcccaccagcagagcctg

cagttaccttgaattgactgtaccttgcccaactggaaacgaggcttgttaattgaattagcttatgggc

agggaatctgttggcaatgccttctggttttctcaagtacactgaacaatcatatccctattgctcaatg

tgcaaatatcatcttttagcgttagcattataggatttcttttcttattaaaaatgccaataattaaaag

caatgggggagttaatgtgagacaacaaggtgcagggctggatgaacacagcaggccaagcagcatcata

ggagcagagaggctgacgtttcggacctagacccttcatcagaaaaaggtgtctctgttcttttgttgtg

caattcatattttagtcatccacataaaaatacaaattgatttgcttttgttaataattgttccatgttt

tgcttgtgttccagccagacctcaatgacagaaaaattccaaaaaagaggaagctccaatcaaacttg**ta**

P D L N D R K I P K K R K L Q S N L -

**g**caaagtattgactgactcttcatcacaattgcatttttatgttcaaatatacttcccaagcagagagaa

tttgttcgatattttgatgttaagatttatgtgagatgtattgatatgtaggaaactatataaaatactt

cacagtagctaagttatatcactactacaaagaatagttcaagtatagataaatattagactgtttccat

tgcctgaaagatgggatgtttccttttcatttttgacaagctgttacattatgtaattatttcgccaaac

aaacatatctgtaagattaggaatgagatgtgtcaccctcttaagtgaccatttaacattctaccacaga

ataacttttctggtagttgccatttttataacataatttactcaaaaatgtcttgacaatcaagtgaaaa

tatgggtaaataaacttcccgtaatagttcgataattacttacgttgtatagtgaaacatattataaatc

aaggaaatagaaaccaccacactcaaaccaaatttacatcccttccaaaagcagcagcatatttatatat

aataaaattgtgtcagatatgaacttagaatctgtcatttacattctaaccactctcctgtcaacttggc

catctatattgggctcttcatcagttttctacttcctcatttattagatctctgtgggtttctactttcg

aactggctattttctattcttcgctaattatccaaaaatttttccacatcataatcaacaaaacaaaaat

aacttctgagttctaatcactttatatcaatgaaaaaatataatttaacaatatatgttatttttaagaa

aaactgattgtgttggttttaacatgtttattttcttttayaaccaatgggttaataatacaacagagaa

ccaaaatgataatagagggtgcagtggaaatcttaaaaaaaattaggcagaaatagaaagaataaaaata

gattagtatcttacataaaaggcaaccggaatgttctgtttcttataaaggcgtaaacactgacaatgtt

gcaatggaaagttggaggtaattggggaagataaagtaagtactgatattccagcagcagaagaggtcct

ggctgaaggaatagtggaagcatattcaattataaattcaaaagagaatttaataaacatatgaaggata

tacgtagaaacagcaaagctctcgggttaatttgatagctttaccaatgtgttgacataggcacaataag

ctaaatggcctacttttacaatcatagacaacaaggtgcagagctggatgaacacagcaggccaagcagc

atcataggagcaggaaggctgacgttttgggcccagacccttcatcagaaatgggggaggggaagggggt

tctgaaataaatagggagagagggggaggcggatagaagatggatagaggagaggataggtggagaggag

acagacaggtcgaagaggcgggaatggagccagtaaaggtgagtgtaggtggggagttagggaggggata

ggtcagtccagggaggacggacaggtcaagggggcgggatgaggttagtaggtaggagatgggtgtgcgg

cttgaggtgggaggaggggagaggtgggaggaagaacaggttagggaggcggggacgagctgggctggtt

ttgggatgcggtaggggaggggagattttgaagcttgtgaaatccacattgataccattgggctgcaggg

ttcccaagcggaatatgagttgctgttcc
