# Supplementary material for: Characterization of Gonadotropin-Releasing Hormone (GnRH) Genes From Cartilaginous Fish: Evolutionary Perspectives
Source: Front Neurosci. 2018 Sep 6;12:607. doi: 10.3389/fnins.2018.00607 (PMC6135963; doi:10.3389/fnins.2018.00607)
Supplement: DATA SHEET S6 — Nucleotide sequence and deduced amino acid sequence of whale shark predicted GnRH1 (A), GnRH2 (B), and GnRH3 (C) coding sequences. Legends are the same as in the Figure 1. [file Data_Sheet_6.DOCX]

A. Whale shark GnRH1 deduced cDNA (partial)

**atg**aagtcacttgtttacctcctgctggcctctgccatcttggcgaatctatcattggcg

**M K S L V Y L L L A S A I L A N L S L A** 20

61 caacactggtccttcgatctgcgtcctggtgggaagcgagcggccggtgacactgtcgtt

**Q H W S F D L R P G G K R** A A G D T V V 40

121 ggagccttccaggat…

G A F Q D

B. Whale shark GnRH2 deduced cDNA

**atg**gctttccagagaaacctgcatttcctggtattcctgctgctgattgttaacactgag

**M A F Q R N L H F L V F L L L I V N T E**  20

61 ttttccacagcccaacactggtcccatggttggtatccaggagggaagagagaagtgagt

**F S T A Q H W S H G W Y P G G K R** E V S 40

121 ctatctcaatctccagatgcttcagaagaaatcaagttatgtcaaggtgagggttgcctg

L S Q S P D A S E E I K L C Q G E G C L 60

181 ttgctgcgaagtccacgtagagggatcataaggagcattgtgatggatatgttggtgcaa

L L R S P R R G I I R S I V M D M L V Q 80

241 cagattcagaagaagaaa**tga**

Q I Q K K K - 86

C. Whale shark GnRH3 deduced cDNA

**atg**gaagttaccaaaacaatttccatccattttctgatagcagtgatgtttattgctcat

**M E V T K T I S I H F L I A V M F I A H 20**

61 ggctgtatctcccagcactggtctcatggttggctgcctggaggaaaaaggagtgcagtg

**G C I S Q H W S H G W L P G G K R**  S A V 40

121 agtatggatgcttatttagagatgataaatgatgaagatgtaataactgactttgagata

S M D A Y L E M I N D E D V I T D F E I 60

181 cccagatatcaatatttatatcagagggcaaataatcctcaagctattataccagacctc

P R Y Q Y L Y Q R A N N P Q A I I P D L 80

241 aatgacagaaaaattccaaaaaagaggaagctccaatcaaacttg**tag**

N D R K I P K K R K L Q S N L - 95
