# Supplementary material for: Characterization of Gonadotropin-Releasing Hormone (GnRH) Genes From Cartilaginous Fish: Evolutionary Perspectives
Source: Front Neurosci. 2018 Sep 6;12:607. doi: 10.3389/fnins.2018.00607 (PMC6135963; doi:10.3389/fnins.2018.00607)
Supplement: DATA SHEET S7 — Nucleotide sequence and deduced amino acid sequence of elephant shark GnRH1a cDNA (A), predicted GnRH1b coding sequence (B), and GnRH2 cDNA (C). Legends are the same as in the Figure 1. [file Data_Sheet_7.DOCX]

A. Elephant shark GnRH1a cDNA

aaccac

7 agccccagacgagggtgatcctcacactaatatttttcccagctgaattgatttgatcat

67 tctcttcttccactgttcgtggggacgtagccggtcacaaaccgacccaagagacgcaat

127 caccgtgtgcgccaacaattcgctgtgaagtgcttttcgacgtgagatccctgcgttaag

187 tacatagattattataacgcaacttataaccttttagccgcggacgggacttaatctatc

247 cggcaattaactggattggactctattctgaggcgacgacggtttgttgagtgtgtgcct

307 gcggtgcgttccctgccccgtggggttctcgctgataccctgctgcgagcctaatgcagc

367 cattatcgcgccgtataaaaaccccaccagaataagccgtcaatcgcaaacccaggagcc

427 acagcagagacgagagctgaacctgccccgaagccccgtgagccgcaagcgtctgcgaga

487 **atg**tccgccctcggcaaaaggctgttgtggctgtctctgaccctggccgtcctcaccgcc

**M S A L G K R L L W L S L T L A V L T A** 20

547 ctgacctcggcacagcattggtccatcgataaccgtcctggaaagaagcgtggcacagag

**L T S A Q H W S I D N R P G K K R** G T E 40

607 cacatgattgaattcctacagggggtcgcaggcgaagtcgaggagctgattcagagcaga

H M I E F L Q G V A G E V E E L I Q S R 60

667 ggaagagctaccgttgagctcccagagtgttcgggagacaacccgggaaaaatggtgttg

G R A T V E L P E C S G D N P G K M V L 80

727 agaaaaaacata**tag**cagtgaaaaaaacagttgagccgctgaatggaatggatcgctgac

R K N I - 84

787 ctgctgcaatcactcacgagcgcgcgagcgcactctctgtgacgtgttaaatctttacca

847 tcagatttgaatgcaggaaataaaccctttccgctctcacgcacgcggctttgcgattta

907 ataacccga**aataaa**tgattcatttgcagctgagaaagttgctcctcggctaaaaagcaa

967 tcctaag

B. Elephant shark GnRH1b deduced cDNA

**atg**tctgtcctcggcaaaaggctgttatggctggttctgatcctggccgtcctcaccgcc

**M S V L G K R L L W L V L I L A V L T A** 20

61 ctgacctcagcacagcattggtccatcgataaccgtcctggaaggaagcgtggcacagag

**L T S A Q H W S I D N R P G R K R** G T E 40

121 cacatgattgaattcctacagggggtcacaggcgaagttgaggagctgttgcagagcaga

H M I E F L Q G V T G E V E E L L Q S R 60

181 ggaagagctaccgttgagcttccagagtgtccaggagacaaaccaagaaaaatggtgtta

G R A T V E L P E C P G D K P R K M V L 80

241 agaaaaaacatg**tag**

R K N M - 84

C. Elephant shark GnRH2 cDNA

ttccaaaccaggca

15  **atg**gctctccagagaaaccttctgctcctgctgctggtgctgctggctattaacactcag

**M A L Q R N L L L L L L V L L A I N T Q** 20

75 gtctcccgagcccagcactggtctcacggttggtacccaggtggaaagagggaactgggc

**V S R A** **Q H W S H G W Y P G** **G K R** E L G 40

135 caagctcagaccccagaggtttcagaagtattccagctgtgtgagggcgatgattgtgcc

Q A Q T P E V S E V F Q L C E G D D C A 60

195 ttcgtgcgaagcccaaggacaaacctgtttagaagcattttggctgacctagtggctgga

F V R S P R T N L F R S I L A D L V A G 80

255 cgatttcagaagaagaag**tga**ggacagattgttggggaagtgactggccacagaggtgac

R F Q K K K - 86

315 gggaacagggggaaaagctcatcagaaacaccattctcaaaacctgcaaagtgtgtgttt

375 gtgcccagcacatgtgggcagtctgtataaaggggaaagttagctccaaggcatgcgact

435 tttgaccggatatcaattcaaatcaagagtgtttcttttggcactgcagttacacctagt

495 aa**aataaa**gactttattttgctacaaacataccctggctccttgtgtgtcattaaccatt

555 attctacccta
