# Supplementary material for: Characterization of Gonadotropin-Releasing Hormone (GnRH) Genes From Cartilaginous Fish: Evolutionary Perspectives
Source: Front Neurosci. 2018 Sep 6;12:607. doi: 10.3389/fnins.2018.00607 (PMC6135963; doi:10.3389/fnins.2018.00607)
Supplement: DATA SHEET S8 — Nucleotide sequences of elephant shark GnRH1a (A), GnRH1b (B), and GnRH2 (C) genes. GnRH1a and GnRH2 are inferred from the cDNA sequences given in Supplementary Data Sheet S7. GnRH1b is inferred from the predicted GnRH1b coding sequence given in the same figure. Legends are the same as in the Supplementary Data Sheet S4. [file Data_Sheet_8.DOCX]

A. Elephant shark GnRH1a gene

**Sc. 563**

…AACAACCACTTGCCTTTGTATGGCATCCTTCAAGTGGTGTAGCGTTCCGGAGCGCTTAACATTCTTCAT

GATAATAATAATAATCCTTTCATTTGATACGGCGCGTCACAGGATCCACTGCAAAGTAAATTGACCGTAT

GTTTTTGAGAGAAACGCGGCAGCCAATTGCGCACAGCAGCGTCCCACAAACAGTCGTAGGTTGAGTGACC

AATTTCTTGGGGGGAAGGAATTATTACCCCCAGAAAAGCGATCCACCAATCAATTTAACAGACGGTTTCC

TGCTTACTGCTAGGGGGGGTAAGGAAACTTGCCTGAGATTTTGCCCTGTCCCGCCCGGGAATCAAACCTA

GGTCCTCTCGCTTGCAAGGCGAGTGCTCTCACCACTGAGCCACAGGACTCCACTGGAGGACTTTGTACTG

AAATGAAAGGCGGGGCTGATCTTGGCCTATCCGCTTGATGGGGATTAAGTGACACGAGCTGCTCTGGGGG

CTTGCCATTGATCTCCGCACCTTGTGTCTCAGGGGCCAATCTGAAAGGAGTGGACATGGAGGGCAGTCAG

ATGACTGGCATCAATCTGCGTGTGGCCACATTAAAGAACGCCAAGCTGAAAAACTGTAATCTCCGAGGGG

CAACGCTGGCGGGCACAGACCTGGAGGTGCGAACAACACATTACATTTACACAGTGCCTTTCACGTCAGG

AGGACGTCCCAAAATGCTGGACAGTCAGGGATCCGCACCCAAGTTGGGGTTGGGATCAGGAGGGGTGGAG

GGCAGAGTAGGAGGGTGACTAATGGCACGGTCGAAGAGGAGGGTCTTGAGAAAAGAGTCTTCCTCATTGT

TATTAGATGAGTGGTGGGGGGTCAACCTTTATAAAACCTTTATAAGTAGAAAGTTGGTGTGGTTTGATGC

TGAGCTGGAGAGGGAGATGGGGAGATGACCGAAGGCTCAGTTGAAGAGGGAAGTCTTCGGGTGGGATTTG

AAGACGAGCAAATGGGGGAGCGGCAAGACTGAGGGATTGAGGCAGTTCCAGTGATTTGATTCCACCCACT

CTTGATTGGTAATGTTATCCCATCCTTTTCCAGAACTGCGACCTGTCCGGTTGTGACCTGCAGGAAGCCA

ACTTGAGGGGCTCAAACGTGAAGGGAGCCATCTTCGAGGAGATGCTGACCGCCTTGCACATGTCTCAGAG

CGTCAGATAAGATCCCGCAGGCGGGAGGGAGGGCGACGTGACCCCCATCAACGAGGAAGAGGAGACGGAA

GTACCAAGACCTCCATAACCCGTGGAGAACAAGTTCATTCAATCCCGTTCTACACACACGCACCTTGCGC

TCACCTATTACAGACACTGCTTCAAGCTGCTCTGGTTCATTTCACACCATACGCTTGGGAGATAGAATCA

GCTGCCCTCCCGTTCTGACTTTACTCCAGATAATTGTTAGCCGGACTCCTTGGGGACATTTTATAACCGT

CTGTGTGTGTGTGTGTGTACACACGCACACAAAGTGGCGCTGGTTAATGTAGAATTTCAGGGAACGTCTT

CCTCCCACAGAATTCTGTCAGGGAGTTTTTGCACGAAAAGAGTTCTCGTTATAAACCGTTGGTGGGGCGG

GTTGGAGGCCGGATCGTGTCCTGTGCCCAAAAGGTAGTTCTCAACAACCGTCGGTGTTAGAGACGGGAGA

GGCGAGTGAGACAACACAGGAACGGGCGACAAAGGGGTTCAAAAGTGAAGTTAATAGGGCAAGGTTGGGG

CAAGAGCAACTTTGGTTGACGTAGTGTGTGGGTGACCTTGACCTTTGCTAGGCCAGGGATTGGGGGGAAT

GGGGGTAAAGAGGGGGAAATGCAATGGTTGCAAAAAGGAGCAAACACGTTTCTCAAGCGCCAAATGAAGC

CGTGTACTTACTGAGGGGGAGCGGTTTCGGTGAGGCTCCGGTACAATGCTTTTAGTCTCGTTCGGGGGCT

GCCAACTCGCTGTGTCTGAACGCGCACACCGAGGGCCACAGCAACCAACGACACTCGTATCGCGAGCGCC

CTTCACGCCGGAAAGACGTCCCAGGGTGCTGGACAATCAGGGGTTTGGGACTCGAGCTGTAAAGCTTAAG

TTTCAGCACTTTGCCGAGCTCCAGGACAAGACTTAACCCCTCTATCTCACCGAGGAGATGCTCTCTTCGT

TTACCTGCAGACGGAAACTATTTGGGCCACAAACTATATACGGTCGATCCGCTTTACAGTATATTCTGCG

AAACTCTTTGAGACGTCTCGAAGGTGTGACGAGGCGCTGTATCAAATGCAACGATTATTATTTACTCAAC

GCAGGACTGGTTTGAATAAACTATTCTCTCAGCATCGCTTGCGGGTTTCGCCATCGGTTCCTTCCAGGAG

TTGCGCTCAGGCGAGAGCGCTGCCTCACCCACGTCACAGCCTTTTAACGTACATCCGTAGCCCAGCGCCA

CATCATTAAACCTGCCTCAGTCAGGCCGTGAAACCCAAAGCTGGTCAGGATTCGCGTAGAAAGGGGATTA

ATTAGTCGGCCGTTTGAACGGGGGAAGCAATTATTGCATTAGTGTTATTATTATTGCTGTAGCTCTGGTT

GGAGCACTTACAAACGAGAGAGCCTGGGTTCGATTCCCCGGCAGTGCGAAACGCTGGGCAAGTTTCCTTA

CTCCACACCCACCCCCTATTTACCTGATATTAAGCAGGAACCTGTTGGATTGCTTTTCCGGGGGTAAATA

GTGCCTTCAAATAAATAAATAATACTTTGGTCACTCAATCCACGACTGTTTGTGGGACATTTCACCCACC

AAATCTACTCGCATACGTGATGAGGCTCTATATCAAACGCAAGGATGATTATCATTATTATTCTCCTGCG

TTTACCCTTTCCAAATATCTATCCCAAGTTCCCCTTCGAAGCTAATCAGGATCTTCCTGAACCACAGCCC

CAGAATAGGGTGGTCCTCACACTAATATTTTTCCCAGCTAAATTGATTTGATCATTCTCTTCTTCCACTG

TTCGTGGGGACGTAGCCGGACACAAACCGACCCAAGAGACGCAATCACCGTGTGCGCCAACAATTCGCTG

TGAAGTGCTTTTCGACGTGAGATCCCTGCGTTAAGTGCAGAGATTATTATAACGCAACTTACAGTGGTAC

CTTTTAGCCGCGGACGGGACTTAATCTATCCGGCAATTAACTGGATTGGACTCTATTCTGAGGCGACGAC

GGTTTGTTGAGTGTGTGCCTGCGGTGCGTTCCCTGCCCCGTGGGGTTCTCGCTGATACCCTGCTGCGAGC

CTAATGCAGCCATTATCGCGCCGTATAAAACCCCACCAGAATAAGCCGTCAATCGCAAACCCAGGAGCCA

CAGCAGAGACGAGAGCTGAACCTGCCTCGAAGCCCCGTAAGCCGCAAGCGTCTGCGAGGTAAGGACAGTT

ACTCGGAGCTTTGCGCCCGTGTTGTAAATCACTCGGGCGCTTTAACTGGAGTCTTGTAGCGCAGTGTTTA

GAGCGCTCGCCTCGGGGTTCGATTCCCGGGCGGGTCAGGGCGAAACACTGGGCAAGTTTCCTTACTCCAC

ACGCACGCCCTCCGTGTACCTAGCAATAAGCAGGAAACTCAGCCGGTTGTTAGTTGATCGGCGGATCACT

TTTCCGAGGGTAAGAATCCCTTCGGGGAAAAAAATAATTCCCGAACGACGTGGTCACTCAACCCGCGGCT

GTTTGTGGGCTGTGCGCAATCGGCCGCCGCGTTTCACCCGCAAAACACACAGTCATTACACTTTACAGTG

TGTCCTGTGAAGCGCTTTGAGACGTCTCGAAGACGTGATGAGGCGCCGGGTCAAATGCGAGGATTATTAT

TATTATTAAGGGCCGGCCGGCGCTCGCTCGGGGCTTTGCGCCTTGTGTCGCACATACCCGGTTTAAATTG

AAGCGCACGGAGAGTAACAGCCGCGTCTTCTTACAGA**ATG**TCCGCCCTCGGCAAAAGGCTGTTGTGGCTG

**M S A L G K R L L W L**

TCTCTGACCCTGGCCGTCCTCACCGCCCTGACCTCGGCACAGCATTGGTCCATCGATAACCGTCCTGGAA

**S L T L A V L T A L T S A Q H W S I D N R P G**

AGAAGCGTGGCACAGAGCACATGATTGAATTCCTACAGGGGGTTTGTATCCTCAGCATTTTCACCCCCTC

**K K R** G T E H M I E F L Q G

GCACCCCCCCCAATCCTCCCATCCCACCCCTCCGCCCCACTCCCTCCTCTTGAAACTGATCCCAATCCCT

TTCCATCTCTCTCCAATCCTCTCGATGCCATTCGCATTGTTGTCACCATTTCTTTTAACGGGACCCACAA

ACCACACAGTCACTTCGCTTTGCAGCACGCCCTGCGAAGCGCTTTGAGACGTCTCGAATGATAATATGAT

GATGATAATAATAATAATAACCTCTGCATTTGATACAGAAATAATTAGTATTATAATAAAAGTGAGTTGG

CCAGAGAGGTACTCAGCGAAATAGAATGGGGAGAGCGCCACTACACCCGTTCTATCGCACTGTGGGTCTC

TCCAGCAGCAGACACTAAACATAATAATCCTGGCATTTGATGTTAGCGCCTTACAGCGCCTTCGACACGT

CTCAAAGCGCTTCACAGGAGGCACCGTAAGGCGGATTGACTGTCAGTGAAGCGGAGCTCGCTCTCGCTCA

AACCCAGAGAGGGAGGAAACGCATTCGTGCAAGTTTTAAGCCGCAGGTGAGGTTGAGGTTGGCGAGAATT

TTTTTTCCCCCCAGTTGCAATCAAAAACCCTGTTCACCGCAGGTCGCAGGCGAAGTCGAGGAGCTGATTC

V A G E V E E L I

AGAGCAGAGGAAGAGCTACCGTTGAGCTCCCAGAGTGTTCGGGAGACAACCCGGTAAGTGCGATAAGCGC

Q S R G R A T V E L P E C S G D N P

AGTGGGCGAGCGGCTGAGACGTAAGCTGCACGTGGCAGCAAATCCCAGCGGAGCGTGTCGAGACTAAATT

CCATCACCGCCCCCATCGCGCCGGTTCCCACTGTTTCCCTATCGCCGAATGGATCGATTTCGAGGCCGTC

ACACTCTTTATTTGAAATCCCTTCACACCCTCCCTACCACTCCCACCTGCTTACCCCCTAAATCACCAAC

CTGCTGACCCCCNNNNNNNNNNNNNNNNNNNNNNNNNNNNNNNNNNNNNNNNNNNNNNNNNNNNNNNNNN

NNNNNNNNNNNNNNNNNNNNNNNNNNNNNNNNNNNNNNNNNNTTTCAGCCACTACGCCCCGAAGCTCTGG

GAACACTCATCCCCTATCCCTCCATCTCGCCCCTTTCCTTCCCCATCTTCAGGGTCCTCTGCGACAGCTC

CTACGGTCACCAAGTCGTTGTTGAGGCGGTCGATATACAGGGCTTGATCACCTTTTGTGTTCCAGGGAAA

G K

AATGGTGTTGAGAAAAAACATA**TAG**CAGTGAAAAAAACAGTTGAGCCGCTGAATGGAATGGATCGCTGAC

M V L R K N I -

CTGCTGCAATCACTCACTCACGAGCGCGCGAGCGCACTCTCTGTGACGTGTTAAATCTTTACCATCTTAT

TTGAATGCAGGAAATAAACCCTTTCCGCTCTCACGCACGCGGCTTTGCGATTTAATAACCCGA**AATAAA**T

GATTCATTTGCAGCTGAGAAAGTTGCTCCTCGGCTAAAAAGCAATCGTAAGAGATGTGGATAAGGCGCTA

TGTCAAATGCAAGGCTCATTATTATATCAAATGCAAGGATTATTATTATTAAATCAAGTGTAAGGATCAT

TGTGTTGCATTTTGGCCACAGCTCAGCCAAATAGCCCTCCTGCGGCCACCACTGCGCAACGCAGAAGCAC

ACACACACAATGCAGTCACTTGACCAGACACGGTGTGAAACCCTCTGTGGCCACAAGAGGGCCCCCACCA

CCCACCCATTCTGTTTGTAAAGTGTAGTGACTGTGTATTTTGAGGGAGGTGTGGGGGGGGGGGTAGGGAG

GGGACCAGGCGGAGGGATTGAGGGAGAGAGCTCTAGAGTTTCGGGGCGCAGTTCCTGACGAGGGGGTTTT

CTCCGGTCAGGAAGGGGTTCAAGTGCTCCGGTTGAGTGAAGTTTGGGGGTTTCCCCCTTCGAGCAGCTCT

GCTCGCCCTGCAGTGGGGAGGGAGACCATTCGACCATTCAATCAATCCTCCTCCGCCGCCTCTCCTCCCA

CCACAACCTCCAAGGTTCAGCCCTTGACTGGTTCAGGTCCTACCTGTCACACCCCCTTCCACTTCGTCGC

AGCAAACGGCTTCTCCTCCGGCCCCCGCGCCGTCTCGTGCCGTGTCCCTCAGGGCTCTGTCCTCGGTCCC

CTCAGCCTCTCTGCCCTCGCACTCTGGAACTCCATCCCACGGTCCCTTCGCCTCGCTTCCTCCCTTTGCC

TCCTTCAAGGTCGGTCTCAAGCCCCTTCTCTTCGAAGCACCGTGCCTTTGGTCGCCCTCCCACCTCTCTG

CCTTCCCTCACCCCTACCCCACCCCTCCCCGATGCTCGGGCTGCGATACCTGATTATCGTGAGGACACAC

AGAGGTTATCGGGGGGGCTCGACTCTACACCCACGACCACCCGGGTGTCTGACACGCACGGAAACCCATC

ACCGCGCTTCAGAGTTTCCAGATCAGCCAACGTTTCCGAAGATGTGGCCACTTCTCCCGGGTTCAAAATG

CCCCTGGAATGTAGACCTCACTCTCCTCTCCCCTCGCAGAGGGTTGGGGGGTCGCGGGGGGGGGTGCAGA

CCCTGGAGCAATCGATGATGTGATGGCTGGCTTTCAACCAGCTCTCTCTTTCTCGCTCTCTCTCCCTTTC

CGAGGTCGGGGAGGAAGGCTTAACGTGTGCTGTGTCCCCGTGCCTGGCGTGTGGTGTGGACATTAGGTTA

AGAGACGGCTTTCCTCGTGACTTGATAAACGATGGGCATTTGTTTTCGCTTGTGACTGCGTCTGTCCGTG

AATCGGGAGCTGTCGGTGAGCGCAGTCTCCCCATTGCCCGTCCCCATTGCGCTGTTTTGAGTGCTTCTTC

ACGTATCGTGTTCCCTTTAAAGAGGGGTAAGCAGCTGATGTACAGCAGCAATCGCCCCCCCCCCCCCCCA

ACTGGGGGACGGTGAGAGAGCAATCACACATCGTCTGACCCCCGAGGCGTGTCTGTTGTGTGCGAGCGTG

TGGGGTGTGCGTGCGCTTAGCTTGTTACGAGACATCAGAAATAAACCAAATGGTGTGATCTGGTGTGATC

CAGCCCGGTGAGCCGGCGTGCGGCTGGGGGTACGGGGGTGGGTGTGGTGGGGGGGTGTGGGGGGGGGGGA

ACCGCTGGCAAACAGCCTCTCGACACCTTTGGGGTTTTGTTTTTGCTCGTTTCCTTTAATTAGTCGCTTC

ACACAACAATCGCCAGAACAGGACGCGGTGACTGGGCCTCTCACTGTGAAACACGGCGATCTCACACGAA

TGTACAAACACCCCCGAGAAAGACGAGAGAGATTTAAAAAAAATATTACACCCATCTAACGCGCAAGTGC

CGTCAGCAACAGCGCGCGCTTCCTAGTCTAGAGGCGGTTTCGAGAGAGCAACTCACCTCCTATTATAATG

CCAGCCGTCAGCAGGGTAAAGCCTTCTCTACGCTATCCTTTTCTCATAATAATTCTAGTAATAACCCCGG

CACTTGATCCGGCGCCTTCCCCTCGCGTCTTCGAGACGTCTCAAAGCGCTTCACAGGATACACTGCGAAG

TGCAGTGACTGTGTATTCCCGTGGGCCAAACTCGGCAGACACTGGCGCCCGGCGGCGAATTGGTTCCTCA

ATCTACGACTGTACGTGGGACAAATTCCCTTTGCGGTGTACCCTGTGGGGTCAACGACGCGATAAGGAGC

TAAACCAAATGCAGGGATGGTTATCATTATTATTATTATTATTGAGTGAAAAATTTAATCTTTTTTTTC…

B. Elephant shark GnRH1b gene

**Sc. 976**

…GGGGTTCGATTCCCGGGCTGATCGGGGCGAAACATTGGGGAAGATTCCTTACTCCACACGCACACCTCT

GTTTACCTAGCGATAAGCATCAACTCAGTCAGTTGTCAGTCGATTGATGGATAGCTTTTCCGGGGGTAAT

AACCCTTCAAAAAAAAACTAATAAATTAATAATGTGGTCACAACAGTTTGTGGGGACGCTGCTGTGCGGA

ATTAACTGCCGCCTTTCACCCACAAAATACTGTCGCTACGCTTTACAGTGTATCCTGTAGAGCGCTTTGA

GACGTCTTGAAGACGTGATAAGGCGCTGTATCAAATGTGAGGATTATTATTATTAAGGACAGGCCAGCGC

TTGCTCTGGGTTTTGCGCCCATGTTGTAAATGCCCGGTTTAACTTTAATTGCGCAGAGAGTAACAGCCGT

GTCTTCTTACAGA**ATG**TCTGTCCTCGGCAAAAGGCTGTTATGGCTGGTTCTGATCCTGGCCGTCCTCACC

**M S V L G K R L L W L V L I L A V L T**

GCCCTGACCTCAGCACAGCATTGGTCCATCGATAACCGTCCTGGAAGGAAGCGTGGCACAGAGCACATGA

**A L T S A Q H W S I D N R P G R K R** G T E H M

TTGAATTCCTACAGGGGGTTTGTATCCCCAGCAGTTTCACACCCAACCCCACCCCCTCCGCCCCATTCTC

I E F L Q G TCCTCTTGAAACTGATCCCAATCGCCATCCTCTCAATGCTATTTACATTGTCATCATTATTTCCTTAATA

AGACATAATGTCCACAAACCACACAGTCACTTCACTTTACAGTATATGCTCTGAAGCGCTTTGAGATGTC

TTGAATAATAATATAATAATAATCCTTGCATTTGATACAGCGCCTTATCACATCTTCGAGACGTCTGAAA

GCGCCTCACAGGAATATACTGTAAAGGGAATTGACTGTATATTCGTGGGCGAAACATGGCAGCCAATTGC

GTACAGCAGCGTCCCACAGACCGTCGTGGATTAAGTGACCGGATTATAATTATTTTGTTTTCTTTCTTGA

GGGATTATTATCAGACGTGCGCAGTAAGGAAAGTTGCCGGGAATCGAACCCAACTCTCGAAGACCCAACT

AAAGGCGCTATATCAAATGCCAGGATTGTTATTATTATTATTATAAAAGACAGTCACTGAGTTGGCCAAA

GAGATACTCAGCGAAATAGAATGGGGAGAGCGCCTTCCTGTGGCTACACCGGGTTTCACATGGTCTGGTC

AAGTGTCTGCGTTGCGTGTTGAGGTGCCACAGAAGGCGCCTCTACACCCGTTCTATTGCACTGAGGATCT

CTCCAGCCACAGAGACAAAATGTAATAGTCCTGGCATTTAATATTAGAGCCTTATCGCGTCTTTGAGACG

TCTCAAAGCGCTTCACAGAATATACTATAAGGTGAATTGACTGTCTGCGAAACAGAGCTCACTCTCGCTC

AAACCCAGAGAGGGAGAAACACATTCATAGAGATTTTAAGCTGCAGGTGAGGTTGGCGAGAATTTTTTTT

AAATTGCAATCAAAACGCTGTTCACCGCAGGTCACAGGCGAAGTTGAGGAGCTGTTGCAGAGCAGAGGAA

V T G E V E E L L Q S R G

GAGCTACCGTTGAGCTTCCAGAGTGTCCAGGAGACAAACCAGTAAGTGCGAAAGCAACAGCGAGCGCCCA

R A T V E L P E C P G D K P

AGATATATGCTACATGTGGCAGCAAATCCCTGTAGAGCGTGTCAAGACTAAACTCTGTCACCCCCCACTC

CATCACACCAGTTCTAGCCAGGGTCCACTGTTTCCCTATCGCCAAATGGATCGATATCAAGATCCTCACA

CTCGTTTTCAAATCCCTTCACACTCAGGCACCTCTCCCACCTGCTCAACCCAATCCCCAACCTGCTGACT

CCGCTCAACTGGCTCCCTCCTCCTGCATGTCCCTCGTGGCATCTGTTCCACCATCGGTGGCCGCGCTTTC

AGCCACTATGCCCTCAACCTGTATTTCCCCATCTCACCGCTTTTCCTTCCCTATCGTCAGGTCGCGCTTG

AAGACCTTCCTCTTCGACCGCACCTACGGTCATCTCCTGTAGTTATTGTTGAGATGGTTGATATACAAGG

CTCGATCATCTTTTGTCTTCCAGAGAAAAATGGTGTTAAGAAAAAACATG**TAG**CAGTGGAAAAACAGTTT

R K M V L R K N M -

AGCCGATGAATGGAGTGACGGGCGCTCATCGATGGGATCGCTGATCCGCTTCAATCACTCACTCAATTAT

GCACTCGCGCACTCTCTGTAATCTGTTAATCTTTGCCGAAAGATTTGAAGCAAAGGAAAATAGGAAATAA

ACTCTTTCCACTCTTACACACGTGCCTTTGCAATTTAATAACAAAAATAAATGAGTATTATTGAATAATA

AATGATTAGTTTACAGCTGAGAAAGTTGCTCTTCAATAAAAAAAGAAATTTGGATCAGGCGCTATATCAA

ATGCCAGGATTATTATTATTAAATCTAATGCCAGGATTATTATATTGTATGTTGCCTACAGCTGGAGAGA

TACTCACCCAAATAGAATGGGGACAGCGCTCTCCTGTCAATGCAACACTGAAAGGCAACACGTTTTTTCT

GACACAAACTCCAAACAGACACAGAGCAGTTCCCTCGTACCAACACTAACAATAATAATAATCCTGGCTT

TCACACAGCGCCTCATCACGTCTTCAAGACGTCTCAATGCGCTTCACAGGATACGCTGTAAAGTGTAGTG

ACCGTGGATTTTGCAGGCGGGGAAGGGAGGGGGCGAGGCGGCAGGATTGAGGGAGAGAGAGAGTGGCGAT

GGTGGAGGCAGCAGGTGAGGCAAAGATTTTCCCCACTTCCATCTCTTTAACCCACATTAGCACATTACAG

CGCTCCCCCCTCCTCCCCCCCCCCACCCTCACCTCCCCCTCCCTCCCCCTCCCACACACACAGAGACACG

AGCACAGATTTGTTTAAAGTTTTATTCCCATTGCCATCCACAAGGTCAATGCACACACTCAGGAACACGT

GCACACACACGTGCAATCGTAGCTCCGCTCGAGAATCCCATTCATTGACATGTTCCGACCTGATATAATT

TAACACAACTTTACAGTGGAGGGTGTGAGGGGGAGGGAGCTAAATAAACCCAAATTTATGTTATTTTCTT

TCTAAAATATATATACAACCTATTATTGATGGAGCCAAAGTGAGTCTGTCTGTGTGTGTGTGTGGGGGAG

GGGGTGACAGACGGCGTGGGGATGGGGAGAAGGGGAGAGAGGGGTCAGGATAGATAACGGGCTCTCTCTC

TCTCTGTTACCTTTTTACTAGTGAATAAAATTTCCCCACTGTGTCGGAGCGATGGACAAACTCCTGCTGA

CACAGGTTAACAAAACATGTAAGACTCCCACTCGCCCCCAAACATCCCCTCGCTCCCCCCCCTCCCTTGC

CCCAATACACCCCACCCTCGACCCCTTTCTCCCGTCATTATTTTGTAGCAGCAAAAACAAACATTGAAAC

CTCCGACACAGTTTTAAATTAACATGTACAGACTCCGCACTCGCTCTCTCTCTCTCTCTCTCGCGCTCAA

CATTGTTCTTCCCTTTTTTTTATGTCAATCAAGCCTTTCCGATTTATTTTCCCCCGTTGGGAAAACTGG…

C. Elephant shark GnRH2 gene

**Sc. 59**

…TTAAAGGTGGGCAAGACTGTCAAATTTGTGCTGGTGGGTTTAGAGTTGATGCTCTCTTCAGTCTCAGGC GACCACCAGCGCAGTCTGATTATGAGTGGTGTCTGTAGGTGTCTGAGTGGTTGGATTAAATCTGCCAGTT

TGACACAAATATTTGGAAAACAAATGTCAGTATTATTCTGTGCTGGGCTGTATCACTCAGTGTTTTGGTA

ATGTTACATTGTCTATAATGTAAAAGTGTGAAACACTATTTCAGTGAGCGAGTGGTAAATCTATGGAGAC

AGTGGAGGCAGATAGTGTCAGTTCATTCAAGTGTAAATTACTTGGATTTATTTTGGAAAATAGTATTTTG

GTTTACAGTATAGGAATAATGTGAGACATGACAAGTGTAGTATGGCTTGGAAGAACCAGGTACCTTTGGA

TCTACGGTTCCCAGAGTTTTCCACTGTGTTGTAAATTAATCCATACAGAATTAATTACTATTAACAGCTC

CATTATTGTAGCAAGAGACTACCAGGATGGTAGAAGGTGAACTAGATGGATCTTAGTCTTTTTTCGTCTA

GCAATTCCTATGCTCCTATGTTTCTATGTAACTCAAACCTATGTTTGATACTGAATCTTACTGCAGACTG

CTGGCCTGAAATTCCATCTTGTCTTAGCTAATAAGACTGACCACGTTGTCTTACAGTTTAAGATGTCACA

GAACTGTGCAAAAATTCTTAAATTCAGCACTAATTTAGAAGTCTTTTTTATGCATTTTTTAAAAATAGAA

GCAGCTTCCAAATTAATAATAATAATAGTAATAATCCTTGGATTTGATATAACGTCTTATCACATTGTTG

AGAAGTTTCAAAGCGCTTCACAGAATATATTGTAAAGTGTATTTTGTGGGCAAAACGTGGCAGCCAATTG

CGCACAGCAGTGTCCCACAAACAGTCGTGGATTGAGTGTTCACGTACAGTGCACATCAGGGCGGCTTCCG

AAAGCGGCAAGTGAGCCTTATCAGGGTGTGCGTGGTGCGCACATGGATGTGGTGCACACAGGGGCGACTC

CCGACTGTGATAAAAGGAGCGTCCCAAAACACGTGTGCACAACCTGTGTGCGGGGAAGGAATGTACCGTA

AACAACGGAATGTGTTTTTGTATCGGGAGAAAGCAAAAAATTGCAACCGACGATGTTGTGTTTGTTTTGG

ATTTCGGTTGTACTAAATATTAGATTTAAAGATAAATAAATATTTTCCAAAAAAAAGTGATTTTATGGAA

AATTATATGAAATAATCCTTGTATTCGATATAGCGCCTTATCACGTTGTTGAGATAATAATAATTATATC

TTTGAATCCCTTTGAGACATAATAATCATTATTATTATGTCTCCAAGGGATTCAAAGATATAATGTAAAA

TGGATTGACTGTGTATTTTGTGGGCGAAATGCGGCAGCCAGTTGTGCACAGCAATATCCCACACACAGTC

ATGGAGTGAGTAACCAATTTTATTTATCTGGGGCATTGTTGGCCAGGACACTGGGAGAGAGAAACTCCCT

TCTCTTTTCGAATAGTGCCACTGTATCTTTAACAGCACCTCCAACAATGCAATAAATCCTCAGTGCTGCA

TTGGATGATGCGATTCCAAATACTTGACTCAGAGGTAAATTATTAATTATTATTAATAATAACATCCCAA

GGTGCTTCTGTCTAGCACCTTGGAACATCTTCCCAATGAGAATGACGCTGTATAAATCTAACTTTGTTCT

TGTTGTGATAAATTGTTGCTGTGAAACATCACAGATTTCCTCCATTACACGCTGCCTAACTGGTACTTGT

TATCAACTCTTAGGATCTCGACAGAAGCCACAGAATTTCCTTCCAAACCAGGCA**ATG**GCTCTCCAGAGAA

**M A L Q R**

ACCTTCTGCTCCTGCTGCTGGTGCTGCTGGCTATTAACACTCAGGTCTCCCGAGCCCAGCACTGGTCTCA

**N L L L L L L V L L A I N T Q V S R A Q H W S H**

CGGTTGGTACCCAGGTGGAAAGAGGGAACTGGGCCAAGCTCAGACCCCAGAGGTCAGTCCTGTTGCCTTT

**G W Y P G G K R** E L G Q A Q T P E

TTCTTTAAATTTGTGGGACTCACAGAGCACTTAATAATAATAATCCTTGCATTTGATATAGAGCTTTATC

TTGTTGGTGAAACATCTCAAAGCGCTTCACAAGCTATATTGTAAAGTGCATTGACTATGTATTTTGTGGG

CAAAACGCAGCAGCCAATTGCGCACAACGGTGTCCCACAAACAGTTTCCTCTCCACGAGTGACATAATAC

AAAAATTACAAAATAATATGAGTAACCCAATTTTTTTTAGGGATTATTACCTCCAGAAAGCAACTAACAC

CCAGGTTCCTACTTACTGTCGTGTAAACCAAGAATGTGAGGCAAGGAAACTTGCCTAACATTTTGCCCCG

ATTGGGAATCAAACTTGGATCCTCTCACTTGCGAGGCGAGTGCACTAACCAATGCGCAACAGGACTCGGG

TTTATTCAGTGAAAGATTCACAAGGGCAGAAATTGAGGCAACAGATTTATAAAGACAGACTTTAAACACA

GCTGATTGTCTCAGTAGGACCACCCTAACCGAGAGGGCTTTGGTTTGCAGGGTAACTAATAAACCTTGCA

TTTGATACAGCACTTTTCATGTCTTCTAGACGTCTCAAAGCTCTTCACAGAATAGACTATTAAGTGAATT

GACTTTATATTTGTGGGCAAAATGCAGCAGCCAATTGCGCACAGCAGTGTCCCATAAACAGTCGTGGATT

GAAGTGACCAATGTATTATTTTTTTCTTGAGGGATTATTATCAGGCGTGTGGTGTAAGGAAACTTGCTCA

AGGTTTCACCCTGCTGGAATCAAACCCAGGTCTCTAGCTTGCGAGGCAAGTGCTCTAACCACTGAGCTAC

AGGACTAAATGGGAGCTGAGTAGGAAGAACCCTTGCTCCCAGTGACTAGTGAGTGCTGTTAAACTAGCAA

AGTTCATAGGAACAAACATTTGCTGTTTTGTTTTAGGTTTCAGAAGTATTCCAGCTGTGTGAGGGCGATG

V S E V F Q L C E G D

ATTGTGCCTTCGTGCGAAGCCCAAGGACAAACCTGTTTAGAAGCATTTTGGTAACTGGGCGTATGCACAC

D C A F V R S P R T N L F R S I L

ACACTGCTGACAGTGCACATATGCCGCACTCTCTCAATCTCTCAATCTCTTTTACTCGCTCTCACTCACT

TTCCCTCCTTGTGTCACTTTCTCTCTCTCTCACACTCTCACTCTCATTTTCTATCTCTCACTCACTCAAA

TTCACTGTCATTCAGTTTCTCTCACTCTTACACTCTCTTACTTTCCCTCACTCTCTCATTCTCTCACTCA

TGCTCATGCTCTCATTCTCTATCTCACTCACTCTCACTCAATCCCTGACTCTTACACACACTCTCTCCCA

AACTTACACTTTCTCTCTCTTTCGCACTCTCTCTCTCTCTTACACACACTCTCACTCTGTCTCTCACACA

CTCTGTCTCTCATATACACACACTCTCTCACACTCTGTCTCTCTCTCACTCTCTCTCACACTGTCTCTCA

CTCTCTCGCACTCTCTCTTGCACACACTCTCTCTCTTAAACTCACAATTGCAGGCTGACCTAGTGGCTGG

A D L V A G

ACGATTTCAGAAGAAGAAG**TGA**GGACAGATTGTTGGGGAAGTGACTGGCCACAGAGGTGACGGGAACAAG

R F Q K K K -

GGGAAAAGCTCATCAGAAACACCATTCTCAAAACCTACAAAGTGTGTGTTTGTGCCCAGCACATGTGGGC

AGTCTGTATAAAGGGGAAAGTTAGCTCCAAGGCATGCGACTTTTGACCGGATATCAATTCAAATCAAGAG

TGTTTCTTTTGGCACTGCAGTTACACCTAGTAA**AATAAA**GACTTTATTTTGCTACAAACATACACTGGCT

CCTTGTGTGTCATTAACCATTATTCTGCCCTTCGGATGAGACATTAAACTGTGGCTTCAGTCGCTACCAC

AGATTGTAGATAGTGGAGCACGGAGACCGGTGAGGAGGGTATTTGAGTATATCCTGTAAAGCACTTTGGG

ACATTCTTCCGACATGAAAGGCGCTGCATCAAATGATTGATGAAAGATTAATATTATTATGTTTATTACG

TTATTATAATTTAAATTGCGCTGTAGAAGTCATTTCTAAGTGGTTTTCAGTGATGGGTCAGCCAGACCAA

TACCACTGCCACTTTATATATGAATGGATGTTACTTTTCTTTGTGTGTTTGAACTTCCTAAAATAGCCTT

ATCACGTTGTTGAGACGTCTCAAAGTCCTGCACGGAATATATTGTAAAGTGGATTGACTATGTATTTCGT

GGACAAAACGTAGCAGCCAATTTTTGCAGGAGGTTCATATTTTGTTCACAAGGTATTTTGTCAAACCATT

TATTATTATTATTATCAGCATTATTATTACCATTATTATTCACAAACAGTAACCACAGGACTCAGGTAAA

GGACAGCACTGGTGTTTATTGTATATTGTTGTGCCAAAGTTTGACATTTATTGCACATTCATCTCAAATG

CATCTGTGTACACATTGGGCCAACTTCAGACAATATCTGCTTAACATTAAAAAGATCAACCCCCACACCC

CTCTCGGTCTCAATTTACTCTAATTTCTCAGCATTTTAGAGCGTCCAGAATTTGACAAATCATTGGCTGA

CATTGTGTTCGACTGAAAACTTTGCAGCCGTTTTTCTTAAAATGTTCCTAAAAAGAAGAGAAACAGAGTG

AATCTTGATCCCAAGTGAGATTTCTCCCTCCCACCTCCACCCAAACCCATCAGTCTGGTCCAGTGAGTCT

ACAGCGTATTGGATGGACATTGAGCATCACTTAGTGGAAAGAATTATTGAGCGTTCAAGAATTTCACACC

AGGGGTCAGATCAGGTCTTGTGTGTGGGGGGGGTTAGTACTGAGCCACGTTAGTCTGATGAGAAGGTGGT

CCTCACGTTACACATGGGGTCCACGGCACAGCTTTCTGTAACACAAACACAGGCATCGCCAATGTCAGAC

AATATCAATGCACAACATGGGATCAGCCATTCTCTCATCCCCGGTATCAACCCAGGGAATCTCCTCTCAG

CCCCTCGATCACCTCGACATCCTTCCTAAACTGGGGTGACCAATACTGGACCTAGCATTCTAACTGAGAG

CCAACCTCCCCCCTCTCTCCAGCTCAGCGTCAAGCTCCACCACTGTTAACATTCTGAGACCTTGCCACAT

GCTATATAAATGCAACTCCCGTCTGCCTGTTTAGGTGGACTTTAAAGATCCCAGGGCACTATTCAATAAG

AGAAGGGAGTTTCTGTTTCCTGTGAACTGGCCAACAATCCCCCAAATAAAATTGGTGACTCAATATTTGC

AGGACTTTGCGGTGCGCAAATTGGCTGCCACGTTCACCCACAAGATGCATAGTCAATGAACTTTACAGTA

TATCCTATGAAGCACTTTGAGACATCTCGATGATGTGATAAGGCGCTATATCAATTGTATGAAAAATACC

ATTTGTTGTTGTTTATTTAGGTTTAGCATTACCTCTTTGGTTTTATAATCTACACCCCGATAAAACTGAG

GATTCCATTTGCTTTTTTGACAGCCTTGCACACCCGGCTTCCCACCTTTAAGGATTTACGTTCGTTAACC

CTCTAAGTCCCGCAGCTGTCATTCTTTTTACTGCTTTAGAAAGGAACCTATGTGGAAATTCACACACGTC

CTGAAAGGGACACCGCCTCACGTTTAACACAAGGCGTAGACCATCCAGAACCCTGCAACTTGCGTCTTCT

CCCGCACCAGGTTCCGTGAGTTCCATCACCTGCATCCTCGCCCGGCTTCACTGGCTCCGCATCACCCAAT

GGATCGTACTCGCATTTAAACCTGCTTCACAACTCGCCCCCTCCCTTTCAAAGCTCACCTCCTCTTTGAC

GATGCTTTTGGTCATCTCCCCCATCCCCCACCACTCCCCCCACCCCATTATTATTTGGACAAACTAACTC

ATACTGACTAGTTTGGCCAAATATTAATAATAATAATGAATATTGTTAATAATATAACGCATAATAATAT

CCTTTCACTTGATAACCGCGCCTTTCAGATCAGAAGGATTCTCAAAGAGCTTCACAGGATGTACTGTAAA

GTGAAGTGACTGTATGTTTGTTAACAAATGCGGCAGCAAATTTGTGTAGCGTGACATCCCAGAAACCACC

ACTCCTAACAAATCTGTGCATGAAAAACATTCTCCTCACGTCCCTATTTACTATCTTAAATTGATCTTC…
