# Supplementary material for: Characterization of Gonadotropin-Releasing Hormone (GnRH) Genes From Cartilaginous Fish: Evolutionary Perspectives
Source: Front Neurosci. 2018 Sep 6;12:607. doi: 10.3389/fnins.2018.00607 (PMC6135963; doi:10.3389/fnins.2018.00607)
Supplement: DATA SHEET S9 — Alignment of the amino acid sequences of chondrichthyan prepro-GnRHs. This figure includes the sequences already displayed in Figure 2 plus the predicted skate GnRHs and elephant shark GnRH1a sequences previously published in (Roch et al., 2014a). Legends are the same as in the Figure 2. [file Data_Sheet_9.DOCX]

**GnRH1**

Catshark ----MKLLVCFALGSAIFVNFLSA**QHWSFDLRPGGKR**EADDDLVESFQEDAGNVDGLT--

Whale shark (p) ----MKSLVYLLLASAILANLSLA**QHWSFDLRPGGKR**AAGDTVVGAFQD-----------

Skate_Roch (p) ------------------------**QHWSYGLRPGGKR**ETNDVMDSTHHVSSLPPHHSLLY

Elephant shark_Roch ------------------------**QHWSIDNRPGRKR**GTEH-MIEFLQGVPQKAPLHPFY

Elephant shark1a MSALGKRLLWLSLTLAVLTALTSA**QHWSIDNRPGKKR**GTEH-MIEFLQGVAGEVEELI--

Elephant sharklb MSVLGKRLLWLVLILAVLTALTSA**QHWSIDNRPGRKR**GTEH-MIEFLQGVTGEVEELL--

**** *** **

Catshark -------HNSR-MGCPFPDCLRGTLAKFTPRRRKL

Whale shark (p) -----------------------------------

Skate1_Roch (p) CRDTRSPHKLALTHT--------------------

Elephant shark_Roch CTEDLSSHRDKMSQAKLRSCCRAVQETNQSR----

Elephant shark1a ------QSRGR-ATVELPECSGDNPGKMVLRKNI-

Elephant sharklb ------QSRGR-ATVELPECPGDKPRKMVLRKNM-

**GnRH2**

Catshark MAFQRNALFLIFLLLIVNTQFSRA**QHWSHGWYPGGKR**ELSLSQSPEVSEEIKLCRGDGCL

Whaleshark MAFQRNLHFLVFLLLIVNTEFSTA**QHWSHGWYPGGKR**EVSLSQSPDASEEIKLCQGEGCL

Elephant Shark ------------------------**QHWSHGWYPGGKR**ELGQAQTPEVSEVFQLCEGDDCA

************** * * ** ** * *

Catshark FLGSPRKDVIRSIVTDMLMQQIQKKK

Whale shark LLRSPRRGIIRSIVMDMLVQQIQKKK

Elephant shark FVRSPRTNLFRSILADLVAGRFQKKK

*** *** * ****

**GnRH3**

Catshark MEVTKIVSVHFLIAIVFTAHGCIS**QHWSHGWLPGGKR**NAVSMDAYLEMVNDEDIITDFEI

Whale shark MEVTKTISIHFLIAVMFIAHGCIS**QHWSHGWLPGGKR**SAVSMDAYLEMINDEDVITDFEI

Skate_Roch (p) ------------------------**QHWSHGWLPGGKR**NAVSMDAYVESPPRRLVSSFLSC

************* ******* *

Catshark PKYQYLYQKMNSPPAYIPDISDRKFQEKRKLQSNLQQNTD

Whale shark PRYQYLYQRANNPQAIIPDLNDRKIPKKRKLQSNL-----

Skate_Roch (p) HSHGRIRCQSASPSAQ------RAW---------------

* * *
